# Design, Synthesis, and Biological Evaluation of New Potential Unusual Modified Anticancer Immunomodulators for Possible Non-Teratogenic Quinazoline-Based Thalidomide Analogs

**DOI:** 10.3390/ijms241512416

**Published:** 2023-08-04

**Authors:** Reda R. Mabrouk, Abdallah E. Abdallah, Hazem A. Mahdy, Samar A. El-Kalyoubi, Omar Jamal Kamal, Tamer M. Abdelghany, Mohamed F. Zayed, Heba K. Alshaeri, Moudi M. Alasmari, Mohamed Ayman El-Zahabi

**Affiliations:** 1 Pharmaceutical Medicinal Chemistry & Drug Design Department, Faculty of Pharmacy (Boys), Al-Azhar University, Cairo 11884, Egypt; redaessa422@yahoo.com (R.R.M.); abdulla_emara@azhar.edu.eg (A.E.A.); hazem_hady2001@azhar.edu.eg (H.A.M.); 2 Directorate of Health Affairs in Buhaira-Clinical Research Department, Ministry of Health and Population, Damanhour 22511, Egypt; 3 Department of Pharmaceutical Organic Chemistry, Port Said University, Port Said 42511, Egypt; s.elkalyoubi@hotmail.com; 4 King Abdulaziz University Hospital, King Abdulaziz University, Jeddah 21461, Saudi Arabia; omarjskamal@gmail.com; 5 Pharmacology & Toxicology Department, Faculty of Pharmacy (Boys), Al-Azhar University, Cairo 11884, Egypt; tamer.abdelghany@azhar.edu.edu.eg; 6 Pharmacology & Toxicology Department, Faculty of Pharmacy, Heliopolis University for Sustainable Development, Cairo 11785, Egypt; 7 Pharmaceutical Sciences Department, Fakeeh College for Medical Sciences, Jeddah 21461, Saudi Arabia; hkalshaeri@fcms.edu.sa; 8 College of Medicine, King Saud Bin Abdulaziz University for Health Sciences (KSAU-HS), Jeddah 21461, Saudi Arabia; asmarim@ksau-hs.edu.sa; 9 King Abdullah International Medical Research Center (KAIMRC), Jeddah 21423, Saudi Arabia

**Keywords:** CRBN, immunomodulatory, quinazoline, thalidomide

## Abstract

Sixteen new thalidomide analogs were synthesized. The new candidates showed potent in vitro antiproliferative activities against three human cancer cell lines, namely hepatocellular carcinoma (HepG-2), prostate cancer (PC3), and breast cancer (MCF-7). It was found that compounds XII, XIIIa, XIIIb, XIIIc, XIIId, XIVa, XIVb, and XIVc showed IC_50_ values ranging from 2.03 to 13.39 µg/mL, exhibiting higher activities than thalidomide against all tested cancer cell lines. Compound XIIIa was the most potent candidate, with an IC_50_ of 2.03 ± 0.11, 2.51 ± 0.2, and 0.82 ± 0.02 µg/mL compared to 11.26 ± 0.54, 14.58 ± 0.57, and 16.87 ± 0.7 µg/mL for thalidomide against HepG-2, PC3, and MCF-7 cells, respectively. Furthermore, compound XIVc reduced the expression of NFκB P65 levels in HepG-2 cells from 278.1 pg/mL to 63.1 pg/mL compared to 110.5 pg/mL for thalidomide. Moreover, compound XIVc induced an eightfold increase in caspase-8 levels with a simultaneous decrease in TNF-α and VEGF levels in HepG-2 cells. Additionally, compound XIVc induced apoptosis and cell cycle arrest. Our results reveal that the new candidates are potential anticancer candidates, particularly XIIIa and XIVc. Consequently, they should be considered for further evaluation for the development of new anticancer drugs.

## 1. Introduction

Cancer is a life-threatening disease [1] and is considered a primary cause of death with growing rates all over the world [2,3]. Cancer cells show a characteristic metabolic profile [4,5]. One of the main problems experienced by the currently used anticancer agents is severe toxicity [3,4]. However, many other anticancer agents, such as thalidomide, are reported to have fewer side effects [5]. Lenalidomide and pomalidomide are thalidomide analogs that are known as immunomodulatory drugs (IMiDs). Thalidomide and its analogs are broadly utilized in cancer treatment [6,7,8]. They have been found to exhibit their significant anticancer effect through different mechanisms, which involve immunomodulatory activities such as co-stimulation of T-cells, inhibition of interleukin-6 (IL-6), IL-1, IL-12, tumor necrosis factor alpha (TNF-α), and nuclear factor kappa B (NFκB), and increasing the production of interferon gamma (IFN-γ), IL10, and IL-2, in addition to inhibition of vascular endothelial growth factor (VEGF), elevation of the levels of caspase-8, and stimulation of apoptosis and cell cycle arrest [6,9,10,11,12,13]. The various different targets of thalidomide may account for its adequacy against numerous types of cancer as well as various autoimmune and inflammatory diseases [3,14,15]. According to these data, thalidomide was selected as a significant lead for the development of new potential anticancer agents.

Thalidomide is a synthetic glutamate derivative that was initially synthesized and introduced in 1957 as a sedative and antiemetic drug for treatment of morning sickness in pregnant women [6,10]. In 1961, various birth problems were reported, which have been attributed to the use of thalidomide in early pregnancy, including phocomelia (lack of arms or hypoplasia), hearing loss, femur and tibia deformities, heart defects, and abnormalities in the gastrointestinal tract, urinary tract, and other internal organs [6,16,17]. This led to the withdrawal of thalidomide from the global market [18]. Later, Sheskin and co-workers observed that thalidomide exhibited immediate and dramatic beneficial effects in the treatment of erythema nodosum (ENL), an acute inflammatory condition associated with erythema nodosum that results in painful skin lesions [19]. This finding generated a lot of interest in further clinical research into the potential immunomodulatory properties of thalidomide.

In 1994, thalidomide-induced teratogenicity appeared to be linked to several significant pharmacological effects including anti-inflammatory effects, inhibition of tumor necrosis factor TNF-α production, and angiogenesis suppression [20]. This led to a new wave of clinical research expanding the utilize of thalidomide for the treatment of different malignancies. Consequently, thalidomide was approved in 1998 by the US Food and Drug Administration for treatment of acute ENL. It was approved in 2006 for treatment of multiple myeloma (MM) as well [21,22,23].

Thalidomide structural modification has resulted in many effective anticancer analogs [24,25]. Avadomide was one of these significant analogs [26]. It was obtained through a structural modification that involved the replacement of the phthalimido moiety of thalidomide by a quinazoline nucleus. Avadomide showed significant immunomodulatory, anti-proliferative, and anti-angiogenic properties [27]. It was found to be active against MM, diffuse large B-cell lymphoma (DLBCL), and solid tumors [26]. Other quinazoline derivatives showed TNF-α, IL-6, and IL1β inhibition [28,29,30], NF-κB inhibition [31], and immunomodulatory [32] and antiproliferative activities [5,18,33,34,35,36], along with induction of apoptosis and elevation of caspase levels [37,38].

Extensive studies showed that cereblon (CRBN) was a primary target of thalidomide and was the critical cause of thalidomide teratogenicity [38]. Cereblon forms an E3 ubiquitin ligase complex with damaged DNA binding protein 1 (DDB1), Cullin-4A (CUL4A), and regulator of cullins 1 (ROC1) [39]. Through a mechanism that has not been completely elucidated, this ubiquitination results in reduced levels of fibroblast growth factor 8 (FGF8) and fibroblast growth factor 10 (FGF10). FGF8, in turn, regulates several developmental processes such as limb and auditory vesicle formation. The net result is that this ubiquitin ligase complex is important for limb outgrowth in embryos [40].

In the absence of cereblon, DDB1 forms a complex with DDB2 that functions as a DNA damage binding protein. Furthermore, cereblon and DDB2 bind to DDB1 in a competitive manner [40].

### Rationale of Molecular Design

**Figure 1:**
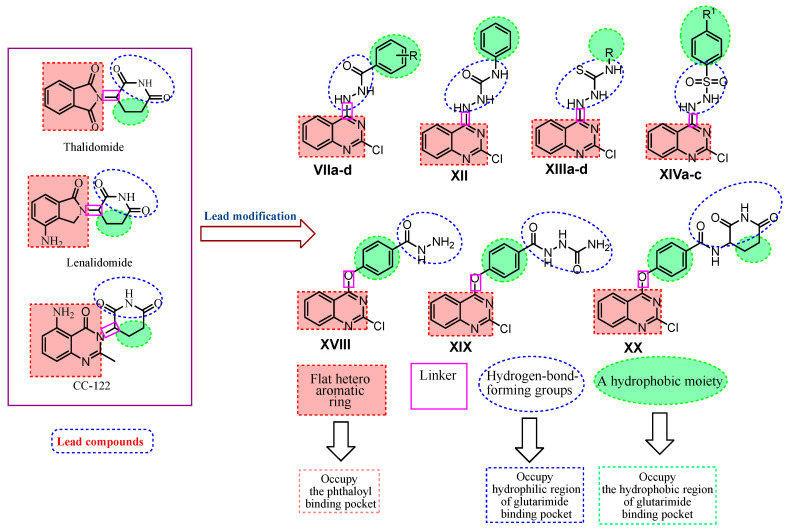
The design of the new candidates according to pharmacophoric features of the lead compounds.

Analyzing the binding interactions of thalidomide and its analogs, lenalidomide, pomalidomide, and avadomide (CC-122), as lead compounds revealed that they shared four common pharmacophoric features for binding, as illustrated in Figure 1. These features include:

1. A flat hetero aromatic ring system that contains at least one H-bond acceptor. It occupies the phthaloyl binding pocket and can form hydrogen bonding interactions with His359 and hydrophobic stacking interactions with Pro354.
2. A hydrophobic moiety interacting with the hydrophobic region of the glutarimide binding pocket forming Van der Waals interactions with Trp382, Trp388, Trp402, and Phe 404.
3. A linker occupying the space region between the glutarimide binding pocket and the phthaloyl binding pocket.
4. A hydrophilic head (three adjacent hydrogen-bond-forming groups) that occupies the hydrophilic region of the glutarimide binding pocket forming hydrogen bonding interactions with His380 and Trp382.

Herein, the rationale of molecular design was dependent on the lead modification of thalidomide at four positions, as illustrated in Figure 1. The first position was the phthalimido moiety (hetero aromatic system), which was replaced with a quinazoline structure as a biological isostere. This is because the quinazoline nucleus was effective in avadomide as an alternative to the phthalimide moiety and it was not suspected before as a cause of teratogenicity. Since the limited flexibility of the thalidomide structure is due to the direct attachment of the glutarimide ring to the phthalimide core, the second fundamental modification was the use of open hydrophilic pharmacophores with different groups of hydrogen bond acceptors and donors to occupy the glutarimide binding pocket. Several effective hydrogen bond acceptors and donors are important to achieve the best binding, including urea, thiourea, sulfamoyl, carbamate, semicarbazide, thiosemicarbazide, and hydrazide moieties. The fourth modification was carried out using different hydrophobic groups to see which one would best accommodate the pocket (see Figure 1).

The aim of this work was to prepare some proposed analogs devoid of the teratogenic effects by adopting some unusual modifications. These modifications were based on some literature findings, such as the following:

- The binding of immunomodulatory drugs (IMiDs), e.g., thalidomide, with CRBN has been associated with teratogenicity. CRBN has a mediating role in helping immunomodulatory drugs to exert their immunomodulatory and tumoricidal effects. However, the idea that cereblon modulation is responsible for the teratogenic activity of thalidomide in chicks and zebrafish was brought into doubt due to a 2013 report that pomalidomide does not cause teratogenic effects in the same animal models of thalidomide, even though it binds with CRBN more strongly than thalidomide [41,42].
- With regard to the cytotoxicity of IMiDs, CRBN likely plays an important role in the binding, ubiquitination, and degradation of factors involved in maintaining the regular function of a cell [43].
- The molecular requirement for ‘thalidomide-type’ teratogenicity is highly structure-dependent. Both the phthalimide and glutarimide groups are essential for embryopathic activity. It is also evident that the glutarimide moiety contributes to the teratogenic effect of many thalidomide analogs because they simply have R and S stereoisomers, where S is a potential teratogen [44].
- Different studies revealed that phthalimide analogs devoid of the glutarimide moiety could be involved in teratogenic effect. Other results indicate that phthalimide analogs devoid of this functional group could represent a new class of analgesic and anti-inflammatory candidates with potentially greater safety [45].

Based on the aforementioned reports, it was decided to investigate several more dramatic unusual modifications of thalidomide. Such modifications are summarized as the following:

Substitution of the phthalimido ring with quinazoline derivatives; hence, the quinazoline ring did not show any reports of embryopathic activity. But, since some of our analogs still have the glutarimide moiety, we decided to open the glutarimide ring as shown below.

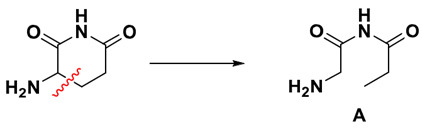



The reason for such ring opening is to deprive the molecule of its chiral center. Therefore, the molecule will lose its chiral center. The non-chiral open-structure product will likely not bind to cereblon in the same manner as thalidomide does and perhaps will no longer be teratogenic. Therefore, it was noted that such an open structure would be like the structures of semicarbazides and thiosemicarbazides simply by changing the methylene group between the NH_2_ and removing the carbonyl group in structure A and substituting it with an R group. We might achieve better binding results by adding a new hydrogen bonding site as shown below:

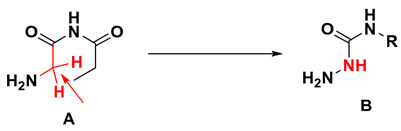



By changing the open structure into a semicarbazide (structure B), where R is an alkyl or phenyl semicarbazide, we thought that these compounds may bind to cereblon in a different manner, through which they can have antiproliferative activity without having embryopathic activity.

Further modification in which we substituted the carbonyl group of structure B with a sulfonamide group as in structure C could add another dimension to our endeavors to improve the biological activity toward a dimension in which the teratogenic effects would almost reach zero probability or at least be dramatically minimized.

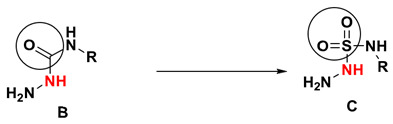



We believe that our modified target compounds could be potential cereblon binders with no or minimum embryopathic activity since they have no chiral centers and may have the different binding mood of cereblon binders.

Finally, adding a phenoxy spacer between these hydrazide groups and the quinazoline ring as in compounds XVII, XIX, and XX could be another choice to evaluate the impact of a phenoxy spacer on the biological activity.

## 2. Results

### 2.1. Chemistry

**Scheme 1:**
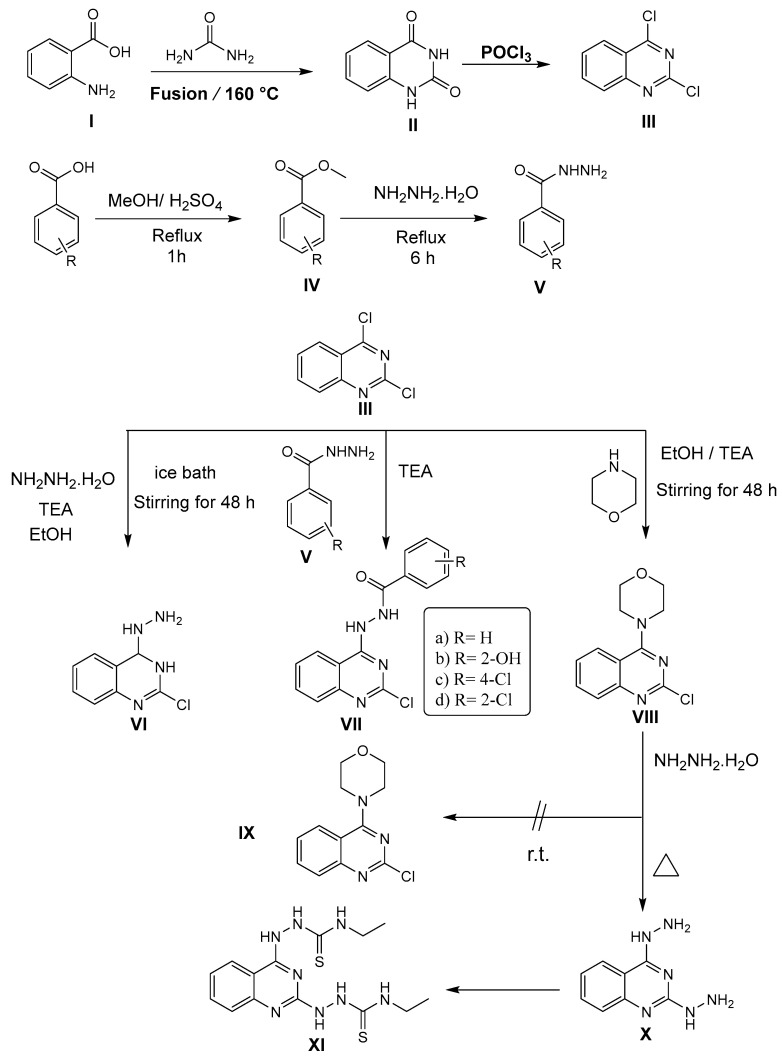
General procedure for synthesis of target compounds III, VIIa-d, X, and XI.

**Scheme 2:**
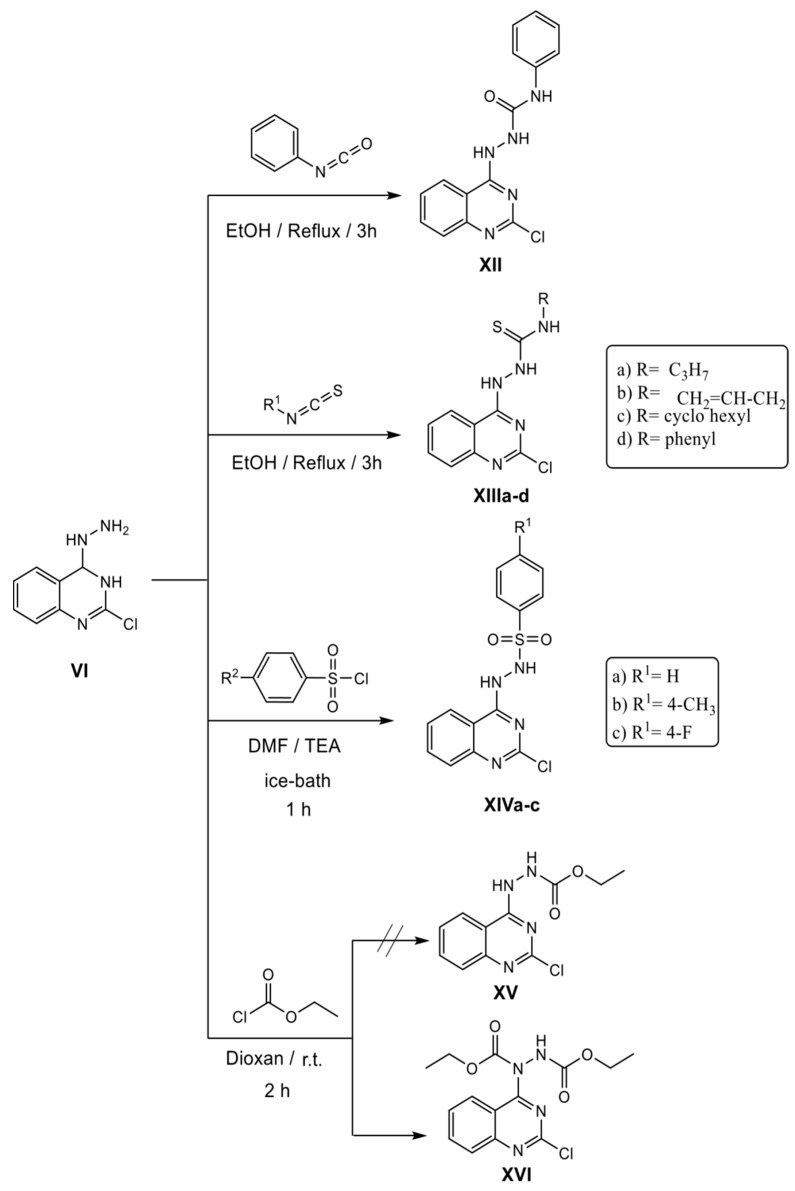
General procedure for synthesis of target compounds XII, XIIIa-d, XIVa-c, XV, and XVI.

**Scheme 3:**
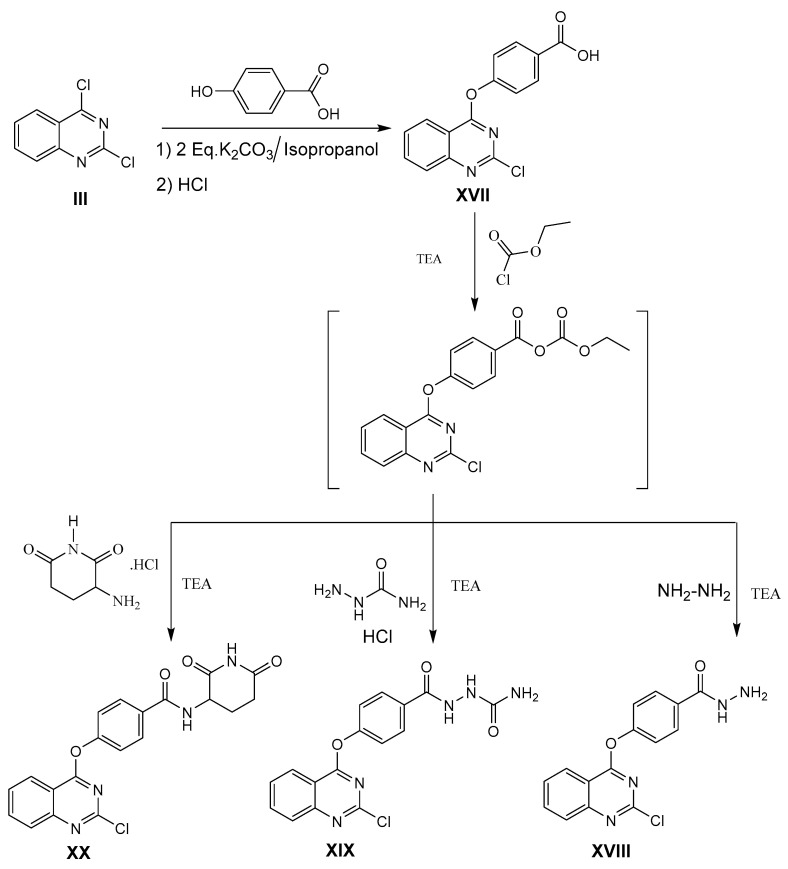
General procedure for synthesis of target compounds XVIII, XIX, and XX.

The sequence of the reactions for the synthesis of our target compounds is described in Scheme 1, Scheme 2 and Scheme 3. Firstly, the starting material quinazoline-2,4(1H, 3H)-dione I was synthesized according to the reported procedures [46,47] through the fusion of anthranilic acid with urea. The chlorination of compound I was achieved using phosphorous oxychloride in the presence of TEA, affording 2,4dichloroquinazoline II [48]. The final compound III was obtained by refluxing II with 3aminopiperidine-2,6-dione in isopropanol. The ^1^H NMR spectra of compound III showed the disappearance of the signals assigned to the free NH_2_ protons of 3-aminopiperidine-2,6-dione. Instead, the spectra exhibited a single proton signal at 8.95 ppm, which indicated the attachment of the amino group to the quinazoline nucleus. An imide proton singlet signal appeared at about 11.0 ppm, which indicated the presence of an intact glutarimide ring.

Ester derivatives IVa-d were prepared by refluxing the appropriate benzoic acid derivatives, namely benzoic acid, 2-hydroxybenzoic acid, 2-chlorobenzoic acid, and 4-chlorobenzoic, in pure methanol containing sulfuric acid as a catalyst. Refluxing the obtained ester derivatives IVa-d with hydrazine hydrate afforded the corresponding acid hydrazides Va-d [49,50], as shown in Scheme 1. The reaction of hydrazine hydrate with 2,4-dichloroquinazoline III in ethanol and in the presence of TEA at 0–5 °C afforded the target compound 2-chloro-4-hydrazinyl3,4-dihydroquinazoline VI [51,52]. The appropriate benzohydrazide derivatives Va-d were stirred with 2,4-dichloroquinazoline III in isopropanol at room temperature for 48 h and in the presence of TEA to afford the corresponding final compounds VIIa-d, as illustrated in Scheme 1. The IR spectra of compounds VIIa-d revealed the presence of characteristic bands in the ranges of 3366–3228 and 1712–1667 cm^−1^, corresponding to NH and C=O groups, respectively. Meanwhile, ^1^H NMR spectra showed the presence of down-field singlet signals in the δ range of 11.18–10.72 ppm, corresponding to NH protons.

The synthesis of 4-(2-chloroquinazolin-4-yl)morpholine VIII was carried out through nucleophilic displacement of the 4-chloro group of 2,4-dichloroquinazoline III with morpholine under basic conditions, as reported in [53]. Refluxing of the latter intermediate with hydrazine hydrate afforded the 2,4-dihydrazinyl derivative X [21,54], as presented in Scheme 1.

When compound 2,4-dihydrazinylquinazoline X was allowed to react with ethyl isothiocyanate, the amino group of the hydrazinyl compound X attacked the electron-deficient carbon of isothiocyanate and afforded the corresponding thiosemicarbazides XI. The ^1^H NMR spectrum of this compound revealed the absence of an NH_2_ signal and appearance of highly de-shielded signals for NH protons. In addition, the ^1^H NMR spectrum showed the appearance of new two triplet signals at δ 1.10 and 1.16 ppm and two quartet signals at δ 3.52 and 3.58 ppm, corresponding to the two ethyl groups attached to the thiosemicarbazide groups.

The semicarbazide compound XII was prepared by refluxing (2-chloroquinazolin4yl)hydrazine VI with phenyl isocyanate in absolute ethanol (Scheme 2). The IR spectrum of this compound demonstrated the presence of a carbonyl absorption band at 1661 cm^−1^. On the other hand, the appearance de-shielded peaks for the three NH protons in the range of δ 10.86–10.00 ppm as well as the absence of the NH_2_ hydrazine peak were noticed in the ^1^H NMR spectrum.

The treatment of compound VI with different isothiocyanates in absolute ethanol yielded the corresponding thiosemicarbazide derivatives XIIIa-d (Scheme 2). The IR spectra of these compounds revealed the presence of NH absorption bands in the range of 3348–3263 cm^−1^. The ^1^H NMR spectra of the compounds revealed the disappearance of the NH_2_ signal and appearance of new singlet signals for the two new NH protons in addition to the alkyl or aryl group protons. The ^1^H NMR spectrum of compound XIIIa, as a representative example, revealed the appearance of a new triplet signal at δ 0.84 ppm, multiplet signal at δ 1.53 ppm, and quartet signal at δ 3.53 ppm, corresponding to the propyl group attached to the thiosemicarbazide group.

The target compounds XIVa-c were synthesized by stirring the hydrazinyl compound VI with the appropriate benzene sulphonyl chloride in DMF containing a catalytic amount of TEA. The IR spectra of these compounds were characterized by the appearance of characteristic bands of the two NH groups in the range of 3727–3295 cm^−1^. Moreover, the ^1^H NMR spectra revealed the appearance singlet signals of NH protons in the δ range of 10.87–10.94 and 10.26–10.64 ppm.

The ethyl hydrazodicarboxylate compound XVI was prepared by stirring the hydrazinyl compound VI with ethyl chloroformate in dioxane. The IR spectrum showed the presence of a characteristic ester carbonyl absorption band at 1778 cm^−1^ and presence of an NH band at 3306 cm^−1^. On the other hand, the ^1^H NMR spectrum showed triplet and quartet signals of the aliphatic ethyl protons at δ 1.16 and 4.24 ppm, respectively. Moreover, the ^1^H NMR spectrum showed a deshielded NH-amide proton at δ 11.41 ppm.

The target intermediate XVII (in Scheme 3) was synthesized through the reaction of 2,4-dichloroquinazoline III with 4-hydroxybenzoic acid under basic conditions.

Compounds XVIII, XIX, and XX were synthesized according to the reported mixed anhydride method [55], which involved treatment of the intermediate XVII with ethyl chloroformate in DCM in the presence of TEA followed by the addition of hydrazine hydrate, semicarbazide HCl, and 3-aminopiperidine-2,6-dione, respectively. The IR spectra of these derivatives showed the absence of absorption bands for a carboxylic group and displayed strong bands for the amidic NH groups at 9.11 ppm, corresponding to NH protons, while the ^1^H NMR spectrum of compound XX showed two signals at about 8.90 and 10.85 ppm, attributed to amide and imide protons, respectively, and lacked the signal assigned for a carboxylic proton.

### 2.2. Biological Evaluation

#### 2.2.1. In Vitro Anti-Proliferative Activity

The MTT assay [56,57,58] was used to assess the in vitro antiproliferative activity of the synthesized compounds against three human tumor cell lines, namely hepatocellular carcinoma (HepG-2), prostate cancer (PC3), and breast cancer (MCF-7), using thalidomide as the standard anticancer drug. The results are summarized in Table 1 as growth inhibitory concentration (IC_50_) values. From the obtained results, it was obvious that the tested compounds displayed excellent, marked, moderate, or weak anti-proliferative activities against the three tested cell lines, as shown in Table 1.

In general, compounds III, XII, XIIIa, XIIIb, XIIIc, XIIId, XIVa, XIVb, and XIVc were the most potent derivatives, with excellent growth inhibitory activity (IC_50_) values ranging from 2.03 to 13.39 µg/mL, exhibiting higher activities than thalidomide against the tested cancer cell lines. Compound XIIIa (IC_50_ = 2.03 ± 0.11, 2.51 ± 0.2, 0.82 ± 0.02 µg/mL) was found to be the most potent counterpart as it was 5.55, 5.81, and 20.82 times more active than thalidomide (IC_50_ = 11.26 ± 0.54, 14.58 ± 0.57, 16.87 ± 0.7 µg/mL) against HepG-2, PC3, and MCF-7 cells, respectively.

In addition, compounds VIIb and VIId had better anti-proliferative activities than thalidomide against MCF-7 cells, but slightly less than the reference against HepG-2 and PC3 cells. Moreover, compounds VIIa and XI showed higher activities than thalidomide against the PC3 and MCF-7 cell lines. Furthermore, compounds VIIc, XVI, and XIX had moderate antiproliferative activities, showing IC_50_ values ranging from 20.35 to 36.49 µg/mL.

It may be beneficial to compare these data to what we published earlier on related compounds. 2-Chloroquinazoline-based XIVb,c showed much better activity than 2,5-dichloroquinazoline-based compounds of similar structures examined in a previous study [7]. This clearly reveals the negative impact of the chlorine atom at position 5 of quinazoline in such a scaffold on the anticancer activity. Moreover, the previous results suggested that phthalazine is a significant alternative to the quinazoline of XIVa-c with respect to antiproliferative effects. However, the immunomodulatory properties of quinazoline derivatives XIVa-c were better to a greater extent than similar phthalazine-based derivatives [7].

For further evaluation, we selected three potent candidates carrying three different chemical groups: semicarbazide XII, thiosemicarbazide XIIIb, and sulfonhydrazide XIVc.

#### 2.2.2. In Vitro Protein Expression Assay

Three compounds, XII, XIIIb, and XIVc, which showed significant antiproliferative results against HepG-2 cells, were selected along with thalidomide as a reference drug for further evaluation of their effect on VEGF, caspase-8, NF-κB P65, and TNF-α expression levels in HepG-2 cells.

##### Estimation of Human Vascular Endothelial Growth Factor (VEGF) in HepG-2 Supernatant

**Figure 2:**
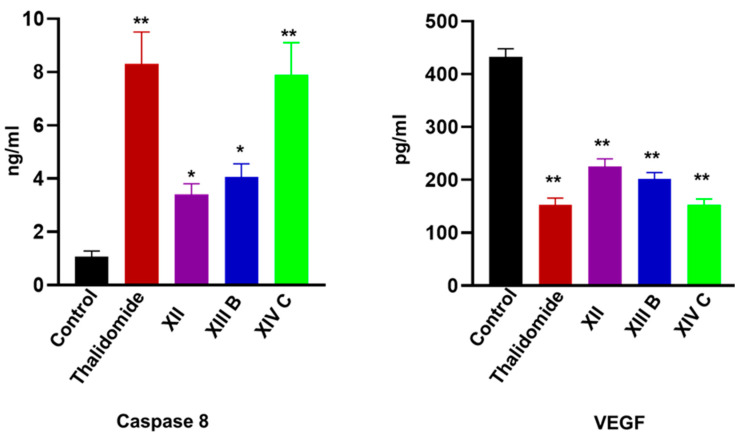
The effect of the selected candidates and thalidomide on the levels of caspase-8 and VEGF. Data are presented as mean ± SD, * denotes significant at *p* ≤ 0.05, and ** denotes significant at *p* ≤0.01. Experiments were repeated three independent times.

The effect of thalidomide and the synthesized compounds XII, XIIIb, and XIVc on VEGF was assessed. The statistical analysis indicated that the effect of XIVc on VEGF levels was comparable to thalidomide. Both significantly decreased VEGF levels to about 153 pg/mL compared to 432.5 pg/mL for the control. Meanwhile, compounds XII and XIIIb decreased VEGF levels by about half (see Table 2 and Figure 2).

##### Caspase-8 Activity Assay in HepG-2 Cell Lysate

The effect of thalidomide and the synthesized compounds XII, XIIIb, and XIVc on the level of human caspase-8 in HepG-2 cells was determined and compared to the control. The data showed that the thalidomide and XIVc results were comparable as they increased caspase-8 levels about 8-fold. Compounds XII and XIIIb increased caspase-8 levels approximately 3- and 4-fold, respectively (see Table 2 and Figure 2).

##### Estimation of Nuclear Factor Kappa-B P65 (NF-κB P65) in HepG-2 Cell Lysate

**Figure 3:**
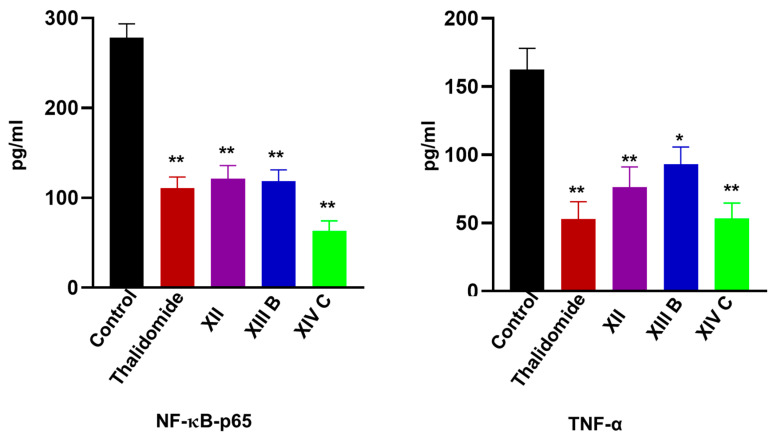
The effect of the selected candidates and thalidomide on the levels of NFκB and TNF-α. Data are presented as mean ± SD, * denotes significant at *p* ≤ 0.05, and ** denotes significant at *p* ≤ 0.01. Experiments were repeated three independent times.

The expression of NF-κB P65 in the cell lysate was determined after the exposure of HepG-2 cells to thalidomide and compounds XII, XIIIb, and XIVc. Then, the expression of NF-κB P65 was compared to that of the control cells. The results showed that there is a significant decrease in NFκB P65 levels after the exposure of HepG-2 cells to thalidomide and the synthesized compounds. Compound XIVc was found to be highly effective and far better than thalidomide and other derivatives. It decreased the levels of NF-κB P65 from 278.1 pg/mL to 63.1 pg/mL compared to 110.5 pg/mL for thalidomide. The two other candidates showed significant results that were slightly lower than that of thalidomide, as shown in Table 2 and Figure 3.

##### The Effects on TNF-α Levels in HepG-2 Cells

The data presented in Table 2 and Figure 3 show that compound XIVc was able to inhibit TNF-α and greatly reduce its levels from 162.5 pg/mL to 53.4 pg/mL. This result was almost equal to that of thalidomide, which decreased TNF-α levels to 53.1 pg/mL. the TNF-α levels in HepG-2 cells treated with XII and XIIIb were 76.4 pg/mL and 93.2 pg/mL, respectively, compared to 162.5 pg/mL in the control.

##### Annexin V-FITC Apoptosis Assay

**Figure 4:**
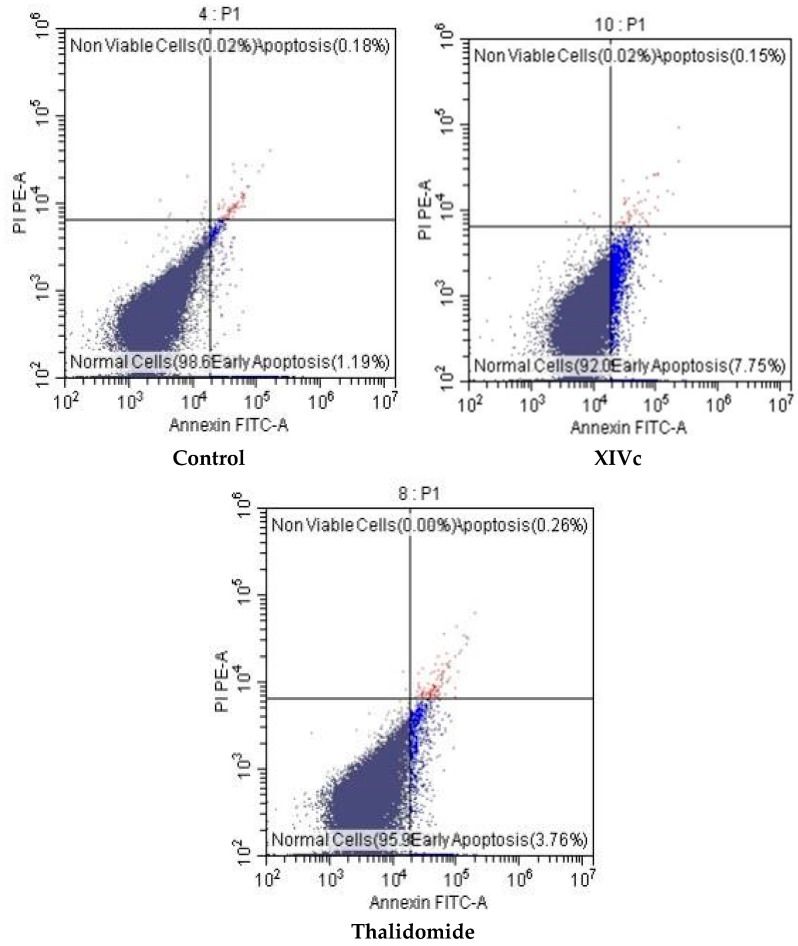
The effect of XIVc and thalidomide on annexin V-FITC-positive staining in comparison with control.

The proapoptotic effect of compound XIVc on HepG-2 cells was evaluated in comparison with thalidomide. Table 3 and Figure 4 show that compound XIVc induced a significant increase in the apoptotic cell population from 1.37% to 7.90%, while thalidomide raised the apoptosis rate to 4.02%. It was clear that the percentage of apoptosis in cells treated with XIVc was two-fold higher than that treated with thalidomide. This indicates the strength of the new derivative as an apoptosis inducer. Of note, the new candidate has no effect on cell necrosis.

##### Cell Cycle Analysis

**Figure 5:**
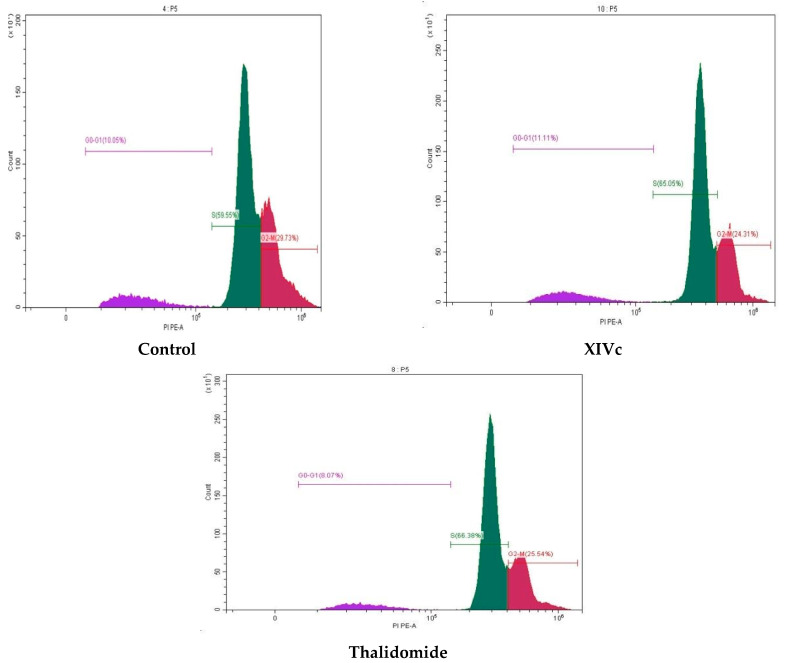
Cell cycle analysis of HepG-2 cells treated with XIVc and thalidomide compared with control.

Cell cycle phases were analyzed for HepG-2 cells treated with XIVc and thalidomide in comparison with control cells. As can be seen from Table 4 and Figure 5, both XIVc and thalidomide caused an increase in the cell population in the S phase, indicating cell cycle arrest in the S phase of the cell cycle. At the same time, they caused a decrease in the percentage of cells in the G2/M phase.

#### 2.2.3. Structure–Activity Relationships (SAR)

By analyzing the results obtained from the antiproliferative assay and the relevant chemical structures, we can deduce the following structure–activity relationships for the new quinazoline-based derivatives:

Quinazolines carrying the sulfonhydrazide moiety were the most potent derivatives, indicating the significance of the sulfonhydrazide group as an open analog to the glutarimide moiety. Similarly, the semicarbazide and thiosemicarbazide groups were found to be highly effective for the same purpose. Small alkyl groups were more effective than bulkier ones with regard to substitution on thiosemicarbazide. With respect to quinazolinylbenzohydrazide derivatives, it was found that unsubstituted phenyl was better than substituted ones, and 2-substituted phenyl was more potent than the 4-substituted one.

## 3. Materials and Methods

### 3.1. Chemistry and Analysis

All melting points were measured using the open capillary method on Gallen lamp melting point apparatus and were uncorrected. The infrared spectra were recorded on a Bruker FT/IR spectrophotometer at the Micro analytical Unit, Faculty of Pharmacy, Ain Shams University, Cairo, Egypt and expressed in wave number (cm^−1^). The ^1^H-NMR spectra were recorded at 400 MHz while the ^13^C NMR spectra were recorded at 100 MHz on a Bruker 400 MHZ-NMR spectrophotometer at the Micro analytical Unit, Faculty of Pharmacy, Ain Shams University, Cairo, Egypt. TMS was used as the internal standard and chemical shifts were measured in ppm. Chemical shifts were expressed in *δ* (ppm) and recorded relative to (DMSO-*d_6_*) solvent, using TMS as the internal reference standard. The progress of reactions was monitored through thin-layer chromatography (TLC) using TLC sheets coated with UV-fluorescent silica gel (Kiesel gel 0.25 mm, 60 F254, Merck Germany) and was visualized using a UV lamp and different developing solvent systems of DCM/methanol and hexane/ethyl acetate mixtures as mobile phases. Microanalyses were performed using a CHN analyzer at the Regional Center for Mycology and Biotechnology, Faculty of Science, Al-Azhar University, Cairo, Egypt. They were within ± 0.4 of the theoretical values. Compounds I, II, III, IVa-d, Va-d, VI, VIII, X, and XVII were synthesized according to previously reported procedures [21,46,47,49,52,53,54,59,60,61,62,63].

#### 3.1.1. General Method for Synthesis of Compound III

To a solution of the appropriate 2,4-dichloroquinazoline derivative II (25.12 mmol) in isopropanol (40 mL), 3-aminopiperidine-2,6-dione hydrochloride (4.96 g, 30.15 mmol) and Et_3_N (5.59 g, 7.99 mL, 55.27 mmol) were added. The reaction mixture was stirred at reflux temperature for 1 h then cooled to room temperature. The obtained precipitates were filtered, washed with water, dried, and crystallized from methanol to afford the target compound.

##### **3-((2-Chloroquinazolin-4-yl)amino)piperidine-2,6-dione III** 

Solid (yield: 61.62%); m.p. = 203–205 °C; IR (KBr, cm^−1^): 3362, 3236 (2NH), 3093 (C-H aromatic), 2878 (C-H aliphatic) and 1707 (CO imide); ^1^H NMR (DMSO-*d6*) *δ* ppm: 2.12–2.26 (m, 1H, CH_2_CH-piperidine), 2.28–2.36 (m, 1H, CH_2_CH-piperidine), 2.59–2.63 (m, 1H, CH_2_COpiperidine), 2.86–2.96 (m, 1H, CH_2_CO-piperidine), 5.16–5.23 (m, 1H, CHCO), 7.59 (dd, 1H, *J* = 7.2 & 8.0 *Hz*, Ar-H), 7.66 (d, 1H, *J* = 8.0 *Hz*, Ar-H), 7.85 (dd, 1H, *J* = 7.2 & 8.0 *Hz*, ArH), 8.29 (d, 1H, *J* = 8.0 *Hz*, Ar-H), 8.95 (d, 1H, *J* = 8.2 *Hz*, NHCH), 10.98 (s, 1H, CONHCO); mass (*m/z*): 293 (M^+^+2, 7.53%), 291 (M^+^, 22.58%), 290 (100%, base peak), 289 (10.28%), 204 (12.96%), 181 (10.51%), 179 (28.27%), 144 (23.41%), 129 (16.15%), 102 (26.38%), 75 (19.08%), 55 (10.81%), 42 913.44); anal. calcd. (found) for C_13_H_11_ClN_4_O_2_ (290.71): **C,** 53.71 (54.04); **H,** 3.81 (3.94); **N,** 19.27 (19.45).

#### 3.1.2. General Procedure for Synthesis of Compounds VIIa-d

To a solution of 2,4-dichloroquinazoline III (0.398 g, 0.002 mol) in isopropyl alcohol (40 mL), the appropriate benzohydrazide derivatives Va-d, namely benzohydrazide, 2-chlorobenzo- hydrazide, 2-hydroxybenzohydrazide, and 4-chlorobenzohydrazide (0.002 mol), were added. The reaction mixture was stirred at room temperature for 48 h. After completion of the reaction, the crude solid was filtered, washed with isopropyl alcohol, and crystallized from ethanol to afford the corresponding derivatives VIIa-d, respectively.

##### ***N′*-(2-Chloroquinazolin-4-yl)benzohydrazide VIIa** 

Yellow-green powder (yield 0.42 g, 70%); m.p. = 210–211 °C. IR (KBr, cm^−1^): 3272 (NH), 1669, (C=O); ^1^H NMR (400 MHz, DMSO-*d*_6_) *δ* ppm; 10.85 (s, 1H, N**H**CO), 10.79 (s, 1H, N**H**- NHCO), 8.40 (d, 1H, *J* = 11.2 Hz, Ar-H, H-8 of quinazoline), 7.99 (d, 2H, *J* = 10.0 Hz, Ar-H, H2 & H-6 of -C_6_H_5_), 7.93 (dd, 1H, *J* = 9.6, 8.0 Hz, Ar-H, H-7 of quinazoline ), 7.75 (d, 1H, *J* = 7.6 Hz, Ar-H, H-5 of quinazoline), 7.62–7.65 (m, 2H, Ar-H, H-6 of quinazoline and H-4 of -C_6_H_5_), 7.56 (dd, 2H, *J* = 11.2, 7.6 Hz, Ar-H, H-3 & H-5 of -C_6_H_5_); anal. calcd. for C_15_H_11_ClN_4_O (298.73): C, 60.31; H, 3.71; N, 18.76; found: C, 60.54; H, 3.44; N, 18.96.

##### ***N′*-(2-Chloroquinazolin-4-yl)-2-hydroxybenzohydrazide VIIb** 

Brown powder (yield 0.42 g, 70%); m.p. = 214–215 °C. IR (KBr, cm^−1^): 3421 (OH), 3366, 3271 (2NH), 1712, (C=O), 1571 (C=N); ^1^H NMR (400 MHz, DMSO-*d*_6_) *δ* ppm; 11.18 (s, 1H, NHCO), 10.80 (s, 1H, NH-NHCO), 8.37 (d, 1H, *J* = 7.9 Hz, Ar-H, H-8 of quinazoline), 8.25 (s, 1H, OH), 7.97 (d, 1H, *J* = 7.7 Hz, Ar-H, H-6 of -C_6_H_4_), 7.86 (d, 1H, *J* = 7.9 Hz, Ar-H, H-5 of quinazoline), 7.72 (d, 1H, *J* = 8.0 Hz, Ar-H, H-7 of quinazoline), 7.63 (m, 1H, Ar-H, H-6 of quinazoline), 7.48 (m, 1H, Ar-H, H-4 of -C_6_H_4_ ), 7.01 (dd, 2H, *J* = 11.2, 7.0 Hz, Ar-H, H-3 & H5 of -C_6_H_4_); anal. calcd. for C_15_H_11_ClN_4_O_2_ (314.73): C, 57.24; H, 3.52; N, 17.80; found: C, 57.58; H, 3.63; N, 17.97.

##### **4-Chloro-*N′*-(2-chloroquinazolin-4-yl)benzohydrazide VIIc** 

Yellow powder (yield 0.43 g, 70%); m.p. = 201–202 °C. IR (KBr, cm^−1^): 3367, 3271 (2NH), 1667 (C=O), 1572 (C=N); ^1^H NMR (400 MHz, DMSO-*d*_6_) *δ* ppm; 10.95 (s, 1H, N**H**CO), 10.82 (s, 1H, N**H**-NHCO), 8.39 (d, 1H, *J* = 14.4 Hz, Ar-H, H-8 of quinazoline), 8.01 (d, 2H, *J* = 11.2 Hz, Ar-H, H-2 & H-6 of -C_6_H_4_), 7.94 (dd, 1H, *J* = 10.8, 9.6 Hz, Ar-H, H-7 of quinazoline.), 7.76 (d, 1H, *J* = 13.2 Hz, Ar-H, H-5 of quinazoline.), 7.64–7.67 (m, 3H, Ar-H, H-6 of quinazoline and H3 & H-5 of -C_6_H_4_); ^13^C NMR (101 MHz, DMSO-*d*_6_) *δ* ppm; 172.34 (C=O). 165.66 (C-2), 157.69 (C-4), 150.93 (C-quat), 137.54 (C-quat), 134.93 (C-quat.), 131.48 (C-quat.), 129.94 (2CH), 129.30 (2CH), 127.42 (CH), 123.36 (CH), 112.46 (CH); anal. calcd. for C_15_H_10_Cl_2_N_4_O (333.17): C, 54.08; H, 3.03; N, 16.82; found: C, 54.31; H, 3.98; N, 16.98.

##### **2-Chloro-*N′*-(2-chloroquinazolin-4-yl)benzohydrazide VIId** 

Yellow powder (yield 0.43 g, 70%); m.p. = 198–299 °C. IR (KBr, cm^−1^): 3228 (NH), 1682 (C=O), 1613 (C=N); ^1^H NMR (400 MHz, DMSO-*d*_6_) *δ* ppm; 10.92 (s, 1H, N**H**CO), 10.72 (s, 1H, N**H**-NHCO), 8.39 (d, 1H, *J* = 8.2 Hz, Ar-H, H-8 of quinazoline), 7.93 (d, 1H, *J* = 8.0 Hz, Ar-H, H-5 of quinazoline), 7.76 (d, 1H, *J* = 8.5 Hz, Ar-H, H-7 of quinazoline), 7.66 (dd, 1H, *J* = 8.0, 8.5 Hz, Ar-H, H-6 of quinazoline), 7.60–7.55 (m, 4H, Ar-H, -C_6_H_4_); anal. calcd. for C_15_H_10_Cl_2_N_4_O (333.17): C, 54.08; H, 3.03; N, 16.82; found: C, 53.95; H, 3.16; N, 16.54.

#### 3.1.3. **2,2′-(Quinazoline-2,4-diyl) bis(N-ethylhydrazine-1-carbothioamide) XI**

Ethyl isothiocyanate (0.175 mL, 0.174 g, 0.002 mol) was added to a solution of compound **X** (0.190 g, 0.001 mol) in absolute ethanol (30 mL). The mixture was heated under reflux for 2 h. After cooling, the precipitate was collected, dried, and recrystallized from ethanol to afford the compound **XI**. White powder (yield 0.255 g, 65%); m.p. = 198–199 °C. IR (KBr, cm^−1^): 3345, 3199 (NH), 2971 (CH aliphatic); ^1^H NMR (400 MHz, DMSO-*d*_6_) *δ* ppm: 10.56 (s, 1H, NH-N**H**-CS), 9.54 (s, 1H, NH-N**H**-CS), 9.30 (s, 1H, N**H**-NH-), 9.23 (s, 1H, N**H**-NH), 8.43 (d, 1H, *J* = 8.2 Hz, Ar-H, H- 8 of quinazoline), 8.11 (dd, 2H, *J* = 8.8, 8 Hz, Ar-H, H-5&H-7 of quinazoline), 7.44 (m, 1H, ArH, H-6 of quinazoline), 7.37 (s, 1H, N**H**-ethyl), 7.14 (s, 1H, N**H**-ethyl), 3.58 (q, 2H, *J* = 6.8 Hz, CH_2_), 3.52 (q, 2H, C**H**_2_), 1.16 (3, 3H, *J* = 7.0 Hz, CH_3_), 1.10 (t, 3H, C**H**_3_); anal. calcd. for C_14_H_20_N_8_S_2_ (364.49): C, 46.13; H, 5.53; N, 30.74; found: C, 46.41; H, 5.29; N,30.49.

#### 3.1.4. **2-(2-Chloroquinazolin-4-yl)-N-phenylhydrazine-1-carboxamide XII**

A mixture of the hydrazinyl compound VI (0.194 g, 0.001 mol) and phenyl isocyanate (0.109 mL, 0.119 g, 0.001 mol) was refluxed in absolute ethanol (30 mL) for 3 h. The solution was cooled and the solid obtained was filtered and recrystallized from ethanol to produce the title compound XII.

White powder (yield 0.31 g, 80%); m.p. = 214–215 °C. IR (KBr, cm^−1^): 3335 (NH), 1661 (C=O), 1590 (C=N); ^1^H NMR (400 MHz, DMSO-*d*_6_) *δ* ppm: 10.86 (s, 1H, -N**H**C_6_H_5_), 10.00 (s, 2H, NH-N**H**CO & N**H**-NHCO), 8.33 (d, 1H, Ar-H, H-8 of quinazoline), 7.78 (m, 2H, Ar-H, H-5 & H-7 of quinazoline), 7.53 (m, 3H, Ar-H, H-6 of quinazoline and H-2 &H-6 of C_6_H_5_), 7.35 (d, 2H, *J* = 7.9 Hz, Ar-H, H-3 &H-5 of C_6_H_5_), 7.18 (m, 1H, Ar-H, H-4 of C_6_H_5_); anal. calcd. for C_15_H_12_ClN_5_O (313.75): C, 57.42; H, 3.86; N, 22.32; found: C, 57.61; H, 3.96; N, 22.14.

#### 3.1.5. General Procedure for Synthesis of Compounds XIIIa-d

A mixture of compound VI (0.194 g, 0.001 mol) and appropriate isothiocyanates, namely propyl isothiocyanate, allyl isothiocyanate, cyclohexyl isothiocyanate, and phenyl isothiocyanate (0.001 mol), in absolute ethanol (25 mL) was heated under reflux for 3 h. Then, the reaction mixture was cooled and the formed precipitate was collected, dried, and recrystallized from ethanol to afford compounds XIIIa-d, respectively.

##### **2-(2-Chloroquinazolin-4-yl)-*N*-propylhydrazine-1-carbothioamide XIIIa** 

Yellow powder (yield 0.22 g, 75%); m.p. = 200–201 °C. IR (KBr, cm^−1^): 3348 (NH), 2924 (CH aliphatic), 1615 (C=N); ^1^H NMR (400 MHz, DMSO-*d*_6_) *δ* ppm: 10.58 (s, 1H, NHN**H**CS, exchangeable with D_2_O), 9.54 (s, 1H, N**H**NHCS, exchangeable with D_2_O), 8.28 (d, 1H, Ar-H, H8 of quinazoline), 7.88 (d, 1H, Ar-H, H-5 of quinazoline.), 7.73 (dd, 1H, Ar-H, H-7 of quinazoline), 7.59 (m, 1H, Ar-H, H-6 of quinazoline), 7.33 (s, 1H, N**H**-propyl, exchangeable with D_2_O), 3.53 (q, 2H, NHC**H_2_**), 1.53 (m, 2H, C**H_2_**CH_3_), 0.84 (t, 3H, CH_3_); anal. calcd. for C_12_H_14_ClN_5_S (295.79): C, 48.73; H, 4.77; N, 23.68; found: C, 48.68; H, 4.91; N, 23.49.

##### ***N*-Allyl-2-(2-chloroquinazolin-4-yl) hydrazine-1-carbothioamide XIIIb** 

Brown powder (yield 0.25 g, 85%); m.p. = 179–180 °C. IR (KBr, cm^−1^): 3342 (NH), 2914 (CH aliphatic), 1612 (C=N); ^1^H NMR (400 MHz, DMSO-*d*_6_) *δ* ppm: 10.62 (s, 1H, NHN**H**CS), 9.67 (s, 1H, N**H**NHCS), 8.53 (s, 1H N**H**-allyl), 8.26 (d, 1H, *J* = 8.0 Hz, Ar-H, H-8 of quinazoline.), 7.88 (d, 1H, *J* = 12.0 Hz, Ar-H, H-5 of quinazoline.), 7.72 (m, 1H, Ar-H, H-7 of quinazoline.), 7.61 (m, 1H, Ar-H, H-6 of quinazoline.), 5.84 (m, 1H, =C**H**-CH_2_), 5.17 (d, 1H, *J* = 12.0 Hz, NHCH_2_=CH-C**H**_2_), 5.07(d, 1H, *J* = 12.0 Hz, NHCH_2_=CH-C**H**_2_), 4.12 (d, 2H, NHC**H**_2_=CH-CH_2_); ^13^C NMR (101 MHz, DMSO-*d*_6_) *δ* ppm: 179.82, 165.75, 154.96, 152.62, 136.11, 132.93, 124.32 (2C), 123.82, 116.33, 115.60, 45.25; anal. calcd. for C_12_H_12_ClN_5_S (293.77): C, 49.06; H, 4.12; N, 23.84; found: C,49.32; H, 4.23; N, 24.09.

##### **2-(2-Chloroquinazolin-4-yl)-*N*-cyclohexylhydrazine-1-carbothioamide XIIIc** 

Brownish white powder (yield 0.25 g, 75%); m.p. = 179–180 °C. IR (KBr, cm^−1^): 3348 (NH), 2925 (CH aliphatic), 1612 (C=N); ^1^H NMR (400 MHz, DMSO-*d*_6_) *δ* ppm: 10.54 (s, 1H, NHN**H**CS), 9.48 (s, 1H, N**H**NHCS), 8.40 (d, 1H, *J* = 9.6, Ar-H, H-8 of quinazoline), 7.96 (d, 1H, *J* = 8.4 Hz, Ar-H, H-5 of quinazoline), 7.72 (dd, 1H, *J* = 9.8, 8.9 Hz, Ar-H, H-7 of quinazoline), 7.61 (d, 1H, *J* = 8.6 Hz, Ar-H, H-6 of quinazoline), 7.18 (s, 1H, N**H **cyclohexyl,), 4.19 (m, 1H, C**H** of cyclohexyl), 1.72 (m, 4H, 2C**H**_2_ of cyclohexyl), 1.58 (m, 2H, C**H**_2_ of cyclohexyl), 1.22 (m, 4H, 2C**H**_2_ of cyclohexyl,); anal. calcd. for C_15_H_18_ClN_5_O (335.85): C, 53.64; H, 5.40; N, 20.85; found: C, 53.87; H,5.56; N, 20.71.

##### **2-(2-Chloroquinazolin-4-yl)-*N*-phenylhydrazine-1-carbothioamide XIIId** 

White powder (yield 0.28 g, 85%); m.p. = 179–180 °C. IR (KBr, cm^−1^): 3263 (NH), 1574 (C=N); ^1^H NMR (400 MHz, DMSO-*d*_6_) *δ* ppm: 10.54 (s, 1H, N**H**-phenyl,), 9.04 (s, NHN**H**CS, 1H), 8.54 (s, N**H**NHCS, 1H), 8.36 (d, 1H, *J* = 9.2 Hz, Ar-H, H-8 of quinazoline), 7.90 (dd, 1H, *J* = 6.8, 8.8 Hz, Ar-H, H-7 of quinazoline), 7.73 (d, 1H, *J* = 6.0 Hz, Ar-H, H-5 of quinazoline), 7.62 (t, 1H, *J* = 10.8, 6.8 Hz, Ar-H, H-6 of quinazoline), 7.50 (d, 2H, *J* = 8.0 Hz, Ar-H, H-2 & H-6 of phenyl), 7.26 (dd, 2H, *J* = 12.0, 8.8. Hz, Ar-H, H-3 & H-5 of phenyl), 6.97 (t, 1H, *J* = 8.0 Hz, ArH, H-4 of phenyl); anal. calcd. for C_15_H_12_ClN_5_S (329.81): C, 54.63; H, 3.67; N, 21.24; found: C, 54.71; H, 3.85; N, 21.52.

#### 3.1.6. General Procedure for Synthesis of Compounds XIVa-c

To a solution of 2-chloro-4-hydrazinylquinazoline VI (0.398 g, 0.002 mol) in DMF (20 mL) containing a catalytic amount of TEA in an ice bath, the appropriate benzenesulfonyl chloride derivatives, namely benzenesulfonyl chloride, 4-methylbenzenesulfonyl chloride, and 4flurobenzenesulfonyl chloride (0.002 mol), were added. The reaction mixture was stirred at room temperature for 1 h. Then, the mixture was poured into cold water (50 mL) and the solid product was filtered, washed with water, and crystallized from ethanol to afford the corresponding derivatives XIVa-c, respectively.

##### ***N′*-(2-Chloroquinazolin-4-yl)benzenesulfonohydrazide XIVa** 

Yellow-green powder (yield 0.59 g, 89%); m.p. = 185–186 ^o^C. IR (KBr, cm^−1^): 3295 (NH), 3064 (CH aromatic), 1572 (C=N), 1336, 1160 (SO_2_); ^1^H NMR (400 MHz, DMSO-*d*_6_) *δ* ppm: 10.92 (s, 1H, NH-N**H**SO_2_), 10.39 (s, 1H, N**H**-NHSO_2_), 8.28 (d, 1H, *J* = 7.2 Hz, Ar-H, H-8 of quinazoline), 7.84–7.80 (m, 3H, Ar-H, H-2 & H-6 of -C_6_H_5_ & H-5 of quinazoline), 7.62 (dd, 1H, *J* = 9.2, 7.6 Hz, Ar-H, H-7 of quinazoline), 7.59 (m, 1H, Ar-H, H-4 of -C_6_H_5_), 7.57 (m, 1H, Ar-H, H-6 of quinazoline), 7.47 (dd, 2H, *J* = 9.6, 8.4 Hz, Ar-H, H-3 & H-5 of -C_6_H_5_); ^13^C NMR (101 MHz, DMSO-*d*_6_) *δ* ppm: 166.69, 154.68, 149.13, 138.40, 133.42, 132.71, 129.49 (2C), 128.05 (2C), 124.51, 122.92, 115.58, 113.46; anal. calcd. for C_14_H_11_ClN_4_O_2_S (334.78): C, 50.23; H, 3.31; N, 16.74; found: C, 50.46; H, 3.19; N, 16.83.

##### ***N*′-(2-Chloroquinazolin-4-yl)-4-methylbenzenesulfonohydrazide XIVb** 

Yellow powder (yield 0.6 g, 85%); m.p. = 180–182 °C. IR (KBr, cm^−1^): 3272 (NH), 1570 (C=N), 1342, 1162 (SO_2_); ^1^H NMR (400 MHz, DMSO-*d*_6_) *δ* ppm: 10.87 (s, 1H, NH-N**H**SO_2_), 10.26 (s, 1H N**H**-NHSO_2_), 8.26 (d, 1H, *J* = 8.1 Hz, Ar-H, H-8 of quinazoline), 7.85–7.81 (m, 2H, Ar-H, H-5 & H-7 of quinazoline), 7.68 (d, 2H, *J* = 7.9 Hz, Ar-H, H-2 & H-6 of -C_6_H_4_), 7.57 (m, 1H, Ar-H, H-6 of quinazoline), 7.27 (d, 2H, *J* = 7.9 Hz, Ar-H, H-3 & H-5 of -C_6_H_4_), 2.33 (s, 3H, CH_3_); anal. calcd. for C_15_H_13_ClN_4_O_2_S (348.81): C, 51.65; H, 3.76; N, 16.06; found: C, 51.76; H, 3.95; N, 16.03.

##### ***N′*-(2-Chloroquinazolin-4-yl)-4-fluorobenzenesulfonohydrazide XIVc** 

Yellow powder (yield 0.6 g, 84.88%); m.p. = 183–185 °C. IR (KBr, cm^−1^): 3272 (NH), 1585 (C=N), 1348, 1168 (SO_2_); ^1^H NMR (400 MHz, DMSO-*d*_6_) *δ* ppm: 10.94 (s, 1H, NHN**H**SO_2_), 10.46 (s, 1H, N**H**-NHSO_2_), 8.28 (d, 1H, *J* = 8.2 Hz, Ar-H, H-8 of quinazoline), 7.87 (d, 2H, Ar-H, H-2 & H-6 of -C_6_H_4_), 7.84 (m, 2H, Ar-H, H-5 & H-7 of quinazoline), 7.57 (dd, 1H, *J* = 8.0, 12 Hz, Ar-H, H-6 of quinazoline), 7.32 (d, 2H, *J* = 5.1 Hz, Ar-H, H-3 & H-5 of -C_6_H_4_); anal. calcd. for C_14_H_10_ClFN_4_O_2_S (352.77): C, 47.67; H, 2.86; N, 15.88; found: C, 47.71; H, 3.04; N, 16.05.

#### 3.1.7. **Diethyl 1-(2-chloroquinazolin-4-yl)hydrazine-1,2-dicarboxylate XVI**

A mixture of hydrazine compound **IV** (0.398 g, 0.002 mol) and ethyl chloroformate (0.142 mL, 0.162 g, 0.0015 mol) in dioxan (20 mL) was stirred at room temperature for 2 h. After the reaction was completed, the precipitate produced was filtered, washed with water, dried, and crystallized from ethanol to afford the corresponding diester derivative XVI.

Brown powder (yield 0.25g, 73%); m.p. = 159–160 °C. IR (KBr, cm^−1^): 3306 (NH), 2980 (CH aliphatic), 1778 (2C=O), 1568 (C=N); ^1^H NMR (400 MHz, DMSO-*d*_6_) *δ* ppm: 11.41 (s, 1H, N**H**), 8.8 (d, 1H, *J* = 6.8 Hz, Ar-H, H-8 of quinazoline), 7.94 (dd, 1H, *J* = 9.2, 7.6 Hz, Ar-H, H-7 of quinazoline), 7.81 (d, 1H, *J* = 6.8 Hz, Ar-H, H-5 of quinazoline), 7.69 (dd, 1H, *J* = 7.6, 6 Hz, Ar-H, H-6 of quinazoline), 4.24 (q, 4H, *J* = 7.3 Hz, 2C**H**_2_), 1.16 (t, 6H, *J* = 7.2 Hz, 2C**H_3_**); anal. calcd. for C_14_H_15_ClN_4_O_4_ (338.75): C, 49.64; H, 4.46; N, 16.54; found: C, 49.82; H, 4.67; N, 16.63.

#### 3.1.8. General Procedure for Synthesis of Compound **4-[(2-Chloroquinazolin-4-yl)oxy]benzohydrazide XVIII**

A mixture of triethylamine (TEA) (0.20 g, 0.274 mL, 0.0022 mol) and 4[(2chloroquinazolin-4-yl)oxy]benzoic acid XVII (0.60 g, 0.002 mol) in DCM (10 mL) was stirred in an ice bath for 15 min. Then, ethyl chloroformate (0.326 g, 0.286 mL, 0.0022 mol) was diluted with DCM and added drop-wise to the previously stirred mixture over a period of 30 min. The reaction mixture was further stirred for 1 h in the ice bath. Then, a solution of hydrazine hydrate, semicarbazide HCl, 3-aminopiperidine-2,6-dione HCl (0.002 mol), and Et_3_N (0.20 g, 0.274 mL, 0.0022 mol) in DCM (15 mL) was added to the reaction mixture. The whole mixture was stirred at r.t. for 24 h and the obtained precipitate was filtered, washed with water, and crystallized from methanol to afford the target compound XVIII.

##### **4-[(2-Chloroquinazolin-4-yl)oxy]benzohydrazide XVIII** 

White powder (yield, 80%); m.p.= 220–221 °C. IR (KBr, cm^−1^): 3395 (NH), 3260 (NH_2_), 1665 (C=O); ^1^H NMR (400 MHz, DMSO-*d*_6_) *δ* ppm: 9.11 (s, 1H, N**H**), 7.89 (d, 1H, *J* = 8.2 Hz, Ar-H, H-8 of quinazoline), 7.63 (m, 2H, Ar-H, H-5 & H-7 of quinazoline), 7.45 (d, 2H, *J* = 7.7 Hz, Ar-H, H-3 & H-5 of -C_6_H_4_), 7.30 (dd, 1H, *J* = 8.2, 7.0 Hz, Ar-H, H-6 of quinazoline), 7.02 (d, 2H, *J* = 7.5 Hz, Ar-H, H-2 & H-6 of -C_6_H_4_), 4.33 (brs, 2H, N**H_2_**); anal. calcd. for C_15_H_11_ClN_4_O_2_ (314.73): C, 57.24; H, 3.52; N, 17.80; found: C, 57.55; H, 3.22; N, 18.05.

##### **2-(4-((2-Chloroquinazolin-4-yl)oxy)benzoyl)hydrazine-1-carboxamide XIX** 

White powder (yield, 80%); m.p. = 254–255 °C. IR (KBr, cm^−1^): 3473 (NH), 3277 (NH_2_), 1651 (C=O); ^1^H NMR (400 MHz, DMSO-*d*_6_) *δ* ppm: 10.04 (s, 1H, NH-N**H**CO), 8.43 (d, 1H, *J* = 13.2 Hz, Ar-H, H-8 of quinazoline), 8.11 (dd, 1H, *J* = 10.4, 7.2 Hz, Ar-H, H-7 of quinazoline), 8.05 (d, 2H, *J* = 10.0 Hz, Ar-H, H-3 & H-5 of -C_6_H_4_), 7.96 (d, 2H, *J* = 7.2 Hz, Ar-H, H-5 of quinazoline &N**H**-NH-C=O), 7.81 (dd, 1H, *J* = 14.4, 7.2 Hz, Ar-H, H-6 of quinazoline), 7.50 (d, 2H, *J* = 11.6 Hz, Ar-H, H-2 & H-6 of -C_6_H_4_), 6.05 (brs, 2H, N**H**_2_); ^13^C NMR (101 MHz, DMSO*d*_6_) *δ* ppm: 173.25, 168.08, 157.01, 154.91, 154.67, 152.73, 136.47, 131.62, 129.97 (2C), 129.04, 127.17, 124.51, 123.22, 122.30, 115.31; anal. calcd. for C_16_H_12_ClN_5_O_3_ (357.75): C, 53.72; H, 3.38; N, 19.58; found: C, 53.59; H, 3.65; N, 19.76.

##### **4-[(2-Chloroquinazolin-4-yl)oxy]-*N*-(2,6-dioxopiperidin-3-yl)-benzamide XX** 

White powder (yield, 80%); m.p. = 230–232 °C. IR (KBr, cm^−1^): 3316 (NH), 1709, 1643 (C=O), 1564 (C=N); ^1^H NMR (400 MHz, DMSO-*d*_6_) *δ* ppm: 10.89 (s, 1H, N**H** piperdine), 8.90 (d, 1H, *J* = 8.4 Hz, N**H**CO), 8.43 (d, 1H, *J* = 8.3 Hz, Ar-H, H-8 of quinazoline), 8.12 (d, 1H, ArH, H5 of quinazoline), 8.03 (d, 2H, *J* = 8.4 Hz, Ar-H, H-3 & H-5 of -C_6_H_4_), 7.97 (dd, 1H, *J* = 8.1 Hz, Ar-H, H-7 of quinazoline), 7.81 (m, 1H, Ar-H, H-6 of quinazoline), 7.52 (d, 2H, *J* = 8.2 Hz, Ar-H, H-2 & H-6 of -C_6_H_4_), 4.82 (d, 1H, *J* = 12.8 Hz, **CH** piperidine), 2.83 (d, 1H, *J* = 20.8, 13.2 Hz, **CH_2_**-piperidine), 2.55 (d, 1H, *J* = 18.4 Hz, **CH_2_**CO-piperidine), 2.17 (d, 1H, 12.8 Hz, **CH_2_**piperidine), 2.02 (m, 1H, **CH_2_**-piperidine); ^13^C NMR (101 MHz, DMSO-*d*_6_) *δ* ppm: 173.54, 172.63, 168.09, 165.93, 154.91, 154.61, 152.75, 136.47, 132.50, 129.69 (2C), 129.04, 127.19, 124.50, 122.41 (2C), 114.86, 50.10, 31.46, 24.65; anal. calcd. for C_20_H_15_ClN_4_O_4_ (410.81): C, 58.47; H, 3.68; N, 13.64; found: C, 58.69; H, 3.84; N, 13.91.

### 3.2. Biological Evaluation

#### 3.2.1. In Vitro Antiproliferative Activities

The in vitro antiproliferative activities of all the synthesized derivatives had already been evaluated for the HepG_2_, PC3, and MCF-7 cell lines using the MTT assay protocol [64], as described in the Supplementary Materials. Thalidomide was utilized as a reference standard. The results were expressed as half maximal growth inhibitory concentration (IC_50_) values ±SD and summarized in Table 1.

#### 3.2.2. In Vitro Protein Expression Assay

Three compounds, XII, XIIIb, and XIVc, exhibiting potent cytotoxic activities against HepG_2_ cells were selected and assessed in vitro for their inhibitory effect on VEGF, caspase-8, NF-κB P65, and TNF-α. Cell culture supernatants and cell lysate were prepared from HepG_2_ and utilized for the immunoassay using different kits [63,65,66,67]. The protocols for cell culture supernatant and cell lysate preparation are written in detail in the Supplementary Materials.

The levels of TNF-α and VEGF in cell culture supernatants were estimated via the ELISA technique using commercially available matched paired antibodies (R&D Systems Inc., Minneapolis, MN) according to a previously reported procedure [67]. On the other hand, the levels of nuclear factor kappa-B P65 (NF-κB P65) and Caspase-8 in HepG_2_ cell lysate were determined according to a previously reported protocol [49,65] described in detail in the Supplementary Materials.

#### 3.2.3. Apoptosis Assay

The annexin V-FITC method was used for this test according to the reported method [68].

#### 3.2.4. Cell Cycle Analysis

This assay was conducted according to the reported procedure [69], as described in the Supplementary Materials.

## 4. Conclusions

Based on the pharmacophoric features of thalidomide and its analogs, sixteen new compounds were designed and synthesized for anticancer evaluation with the aim to design some new dimensions of potent immunomodulators with non-teratogenic analogs. The new candidates were found to exhibit potent antiproliferative activities against hepatocellular carcinoma (HepG2), prostate cancer (PC3), and breast cancer (MCF-7). Compound XIIIa emerged as the most potent candidate, revealing antiproliferative results better than thalidomide and other quinazolone-based thalidomide analogs (compound III). Furthermore, compound XIVc was superior to thalidomide in terms of the antiproliferative assay, apoptosis induction, and reduction in NF-κB P65 levels in HepG-2 cells. Moreover, it was comparable to thalidomide in terms of lowering TNF- and VEGF levels while increasing caspase-8 levels. Accordingly, this work indicates that compounds XIIIa and XIVc are apparently significant lead compounds for further evaluation and modification in order to develop new immunomodulatory anticancer drugs with possible non-teratogenic effects.

## Figures and Tables

**Table 1:** In vitro anti-proliferative activities of the tested compounds toward HepG-2, PC3, and MCF-7cell lines.

Compound No.	In Vitro Cytotoxicity IC_50_ (µg/mL) ^a,b^		
	HepG-2	PC3	MCF-7
III	9.73 ± 0.66	13.52 ± 0.28	23.14 ± 0.4
VIIa	16.16 ± 0.67	11.23 ± 0.54	8.82 ± 0.45
VIIb	12.72 ± 0.635	15.45 ± 0.69	11.29 ± 0.55
VIIc	20.35 ± 0.73	25.47 ± 0.79	30.61 ± 0.89
VIId	16.31 ± 0.69	19.54 ± 0.72	12.78 ± 0.58
XI	13.90 ± 0.87	12.24 ± 0.83	6.18 ± 0.5
XII	4.57 ± 0.12	5.82 ± 0.15	3.61 ± 0.1
XIIIa	2.03 ± 0.11	2.51 ± 0.2	0.82 ± 0.02
XIIIb	7.83 ± 0.46	9.36 ± 0.47	6.89 ± 0.24
XIIIc	9.72 ± 0.54	10.12 ± 0.51	7.19 ± 0.43
XIIId	11.24 ± 0.55	13.39 ± 0.64	10.24 ± 0.53
XIVa	6.76 ± 0.23	7.67 ± 0.44	4.81 ± 0.13
XIVb	3.41 ± 0.1	6.73 ± 0.23	2.8 ± 0.06
XIVc	2.14 ± 0.05	4.98 ± 0.15	5.57 ± 0.16
XVI	21.64 ± 0.75	31.01 ± 0.81	20.16 ± 0.74
XIX	21.32 ± 0.74	36.49 ± 1.25	21.98 ± 0.78
XX	16.62 ± 0.7	20.23 ± 0.73	13.45 ± 0.67
Thalidomide	11.26 ± 0.54	14.58 ± 0.57	16.87 ± 0.7

^a^ IC_50_ values are the mean ± S.D. of three separate experiments. ^b^ IC_50_ (µg/mL).

**Table 2:** The effect of the selected candidates and thalidomide on the levels of caspase-8, VEGF, NFκB, and TNF-α.

Comp. No.	Caspase-8 (ng/mL)	VEGF (pg/mL)	NFκB P65 (pg/mL)	TNF-α (pg/mL)
XII	3.4 ± 0.4 *	225.1 ± 17.2 **	121.3 ± 14.6 **	76.4 ±14.6 **
XIIIb	4.05 ±0.5 *	201.5 ± 19.5 **	118.5 ± 18.3 **	93.2 ± 12.5 *
XIVc	7.9 ± 1.2 **	152.6 ± 14.5 **	63.1 ± 11.05 **	53.4 ± 11.2 **
Control	1.08 ± 0.2	432.5 ± 25.5	278.1 ± 18.5	162.5 ± 15.5
Thalidomide	8.3 ±1.2 **	153.2 ± 13.4 **	110.5 ± 13.2 **	53.1 ±12.5 **

Data are presented as mean ± SD, * denotes significant at *p* ≤ 0.05, and ** denotes significant at *p* ≤ 0.01. Experiments were repeated three independent times.

**Table 3:** Effect of compound XIVc and thalidomide on the percentages of apoptosis and necrosis in HepG-2 cells compared with control cells.

Compound	Normal Cell	Early Apoptosis	Late Apoptosis	Total Apoptosis	Necrosis
Control	98.61 ± 5.7	1.19 ± 0.7	0.18 ± 0.01	1.37 ± 0.09	0.02 ± 0.0
Thalidomide	95.98 ± 12.3	3.76 ± 0.9	0.26 ± 0.017	4.02 ± 0.1	0.00 ± 0.0
XIVc	92.08 ± 11.23	7.75 ± 1.2 *	0.15 ± 0.03	7.90 ± 0.1	0.02 ± 0.0

Data are presented as mean ± SD and * denotes significant at *p* ≤ 0.05. Experiments were repeated three independent times.

**Table 4:** Percentages of cell cycle stages of HepG-2 cells treated with XIVc and thalidomide compared with control.

Compound	G0/G1	S	G2/M
Control	10.05	59.55	29.73
Thalidomide	8.07	66.38	25.54
XIVc	10.64	65.05	24.31

## Data Availability

Not applicable.

## Supplementary Materials

The following supporting information can be downloaded at: https://www.mdpi.com/article/10.3390/ijms241512416/s1.
