# Supplementary material for: Design, Synthesis, and Biological Evaluation of New Potential Unusual Modified Anticancer Immunomodulators for Possible Non-Teratogenic Quinazoline-Based Thalidomide Analogs
Source: Int J Mol Sci. 2023 Aug 4;24(15):12416. doi: 10.3390/ijms241512416 (PMC10418715; doi:10.3390/ijms241512416)
Supplement: Supplementary file 1 [file ijms-24-12416-s001.zip › ijms-2484070-supplementary.pdf]

Biological testing

1. *In vitro* anti-proliferative activity

MTT assay protocol was applied as described previously to assess the anti-proliferative activity of the synthesized compounds. Three human cancer cell lines (HepG2, PC3 and MCF-7 were used in this test. Briefly, cells were cultured in RPMI-1640 medium supplemented with 10% fetal bovine serum and 100 units/ml penicillin and100 µg/ml streptomycin, at 37°C in a 5% CO_2 incubator. Cells were seeded in a 96-well plate at a density of 5 x 10^3 cells / well and left overnight to attach. Cells were treated with different concentrations of the synthesized compounds and incubated for 72 h. 50 µl of MTT solution at 5mg/ml was then added and incubated for 4 h. 100 µl Dimethyl sulfoxide (DMSO) was added into each well to dissolve the purple formazan crystals. The developed color was measured at 570 nm using a plate reader (EXL 800, USA). The relative cell viability in percentage was calculated as (A570 of treated samples/A570 of untreated sample) X 100. The half maximal inhibitory concentration (IC_{50}) values were calculated using non-linear regression analysis of the data obtained from three independent experiments. Data were presented as mean ±SD.

2.1. Supernatant preparation

Cell culture supernatants were prepared from $HepG_2$ cell line. Cells were cultured in RPMI 1640 medium supplemented with 10% fetal bovine serum (FBS) , 100 U/ml of penicillin and 100 µg/ml of streptomycin at 37 °C in a humidified 5% CO2 incubator. The candidate compounds and thalidomide were dissolved in DMSO as a stock solution at 100 mmol/L and diluted with an FBS-free medium to achieve the designated concentrations (IC_{50}). The same concentration of DMSO without any compounds was used as a control. 0.5x10^6 cells $HepG_2$ cells were cultured into T25 flask, allowed to adhere overnight followed by treatment with the tested compounds. After 72 h, the media was collected and centrifuged for 15 min at 5000 rpm and equal volume of cell culture supernatants were collected and utilized for immunoassay using different kits [1].

2.2. Cell lysate preparation

The tested compounds and thalidomide were incubated for 72 h with $HepG_2$ cells. Then, the cells were collected by trypsinization using 0.25 % trypsin/EDTA solution. Cell palettes then were washed three times with phosphate buffer saline (PBS) and lysed by three repetitive freezing/thawing cycles (thawing at 37 °C for 2 min and freezing at –80 °C for 15 min), followed by homogenization of the cells by passing through a 20G needle [2].

2.3 Estimation of NF-κB P65 and Caspase-8 levels in $HepG_2$ cell lysate.

50 µL/well of cell lysate or standard were applied to the microtiter plates pre-coated with the specific monoclonal antibodies of caspase-8, and NF-κB P65 and incubated at 37 °C for 90 min. All wells were washed three times using the wash buffer to remove any unbound proteins. Biotinylated detection antibody was then added, followed by incubation at 37 °C for 60 min. The solution was aspirated and washed three times with the wash buffer. Horseradish peroxidase (HRP) conjugated streptavidin was added to all wells and incubated for 30 min at 37 °C . At the end of the incubation period, the solution was removed and 90 µl of substrate reagent was added to each well and incubated at 37 °C for 15 min. To stop the reaction, 50 µl of stop solution was added and then the absorbance was measured immediately at 450 nm using the ELISA plate reader (FLUO star OPTIMA. Concentrations of caspase-8 and NF-κB P65 were determined by interpolating the absorbance values with that of the standard curve obtained from the known concentrations of standard proteins. [2].

2.3 Estimation of VEGF and TNF-α in $HepG_2$ levels cell culture supernatant.

In order to quantify the VEGF and TNF-α protein that was secreted extracellularly by HepG2 cells, following drug treatment, the media content was analyzed by ELISA using VEGF and TNF-α ELISA kits (E-EL-H1600, Elabscience and ab181421, abcam) respectively, as per manufacturer’s instructions.

3.Cell cycle analysis

To determine the effect of the synthesized compounds on cell cycle distribution, cell cycle analysis was performed using propidium iodide (PI) staining and flow cytometry analysis for compound **XIVc** Flow Cytometry Kit for Cell Cycle Analysis (ab139418_Propidium Iodide Flow Cytometry Kit/BD) was used in this test. HepG2 cells were treated with the IC_{50} of compound **XIVc** for 72 h. Then, the cell pellets were fixed in 70% ethanol at 4 °C for 12 h. After that, cells were washed with cold PBS, incubated with 100 µl RNase A at 37 °C for 30 min, and stained with 400 µl PI in the dark at room temperature for further 30 min. The

stained cells were measured using Epics XL-MCL™ Flow Cytometer (Beckman Coulter), and the data were analyzed using Flowing software (version 2.5.1, Turku Centre for Biotechnology, Turku, Finland).

4. Apoptosis assay

Flow cytometry was used to investigate the effect of synthesized compounds on cellular apoptosis. HepG2 cells were treated with compound **XIVc** at IC_{50} for 72 h, cells then were collected by trypsinization, centrifuged, washed three successive times with PBS, suspended in 500 µl binding buffer, and double stained with 5 µl Annexin V-FITC and 5 µl PI in the dark at room temperature for 15 min. The stained cells were measured using Epics XL-MCL™ Flow Cytometer and analyzed using the machine provided software.

References

1. Dawoud, N.T.A.; El-Fakharany, E.M.; Abdallah, A.E.; El-Gendi, H.; Lotfy, D.R. Synthesis, and docking studies of novel heterocycles incorporating the indazolylthiazole moiety as antimicrobial and anticancer agents. Sci. Rep. 2022, 12, 3424.
2. Abdallah, A.E.; Mabrouk, R.R.; Elnagar, M.R.; Farrag, A.M.; Kalaba, M.H.; Sharaf, M.H.; El-Fakharany, E.M.; Bakhotmah, D.A.; Elkaeed, E.B.; Al Ward, M.M.S. New Series of VEGFR-2 Inhibitors and Apoptosis Enhancers: Design, Synthesis and Bi-ological Evaluation. Drug Des. Devel. Ther. 2022, 16, 587–606.

1HNMR for comp. VII_a

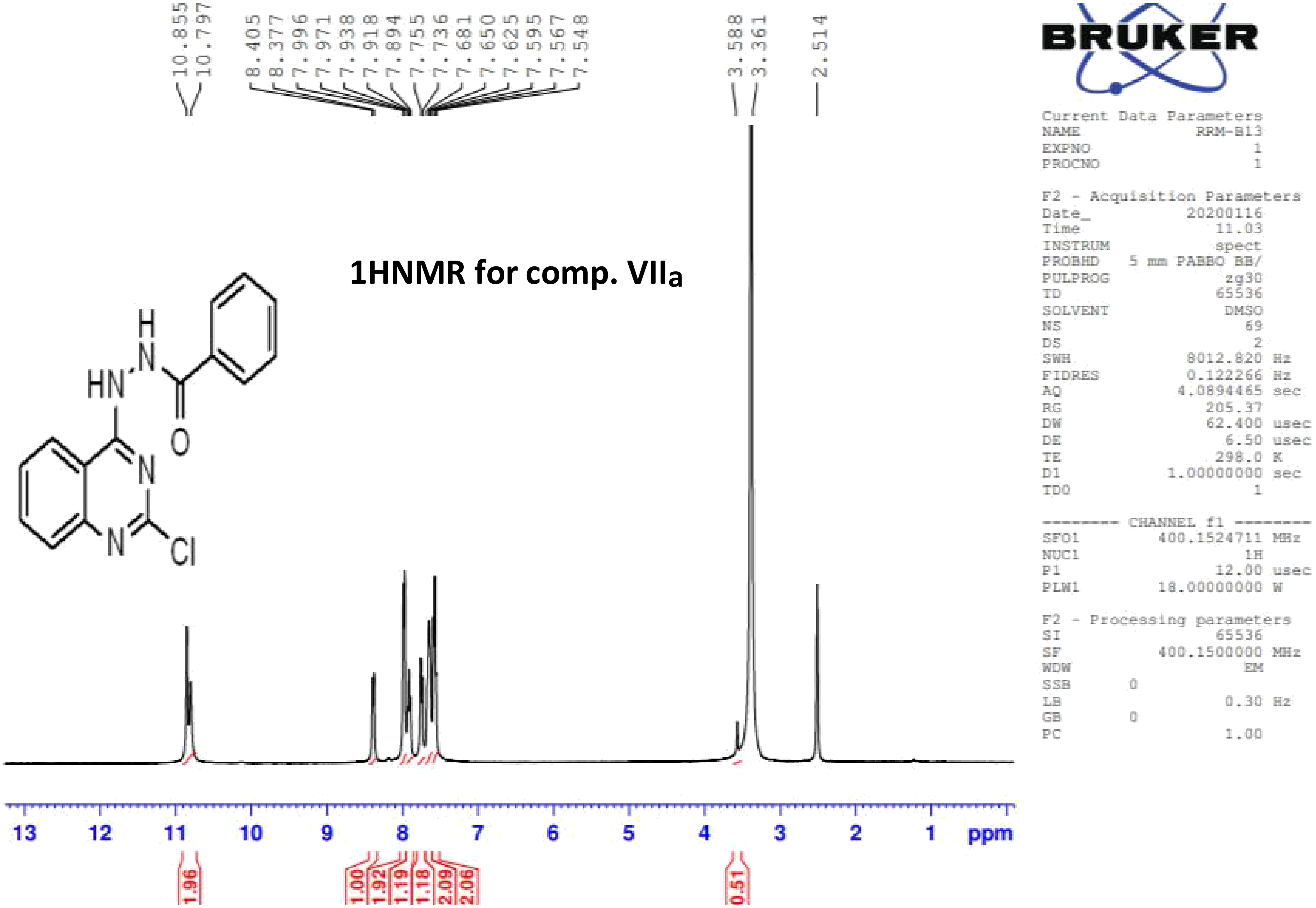

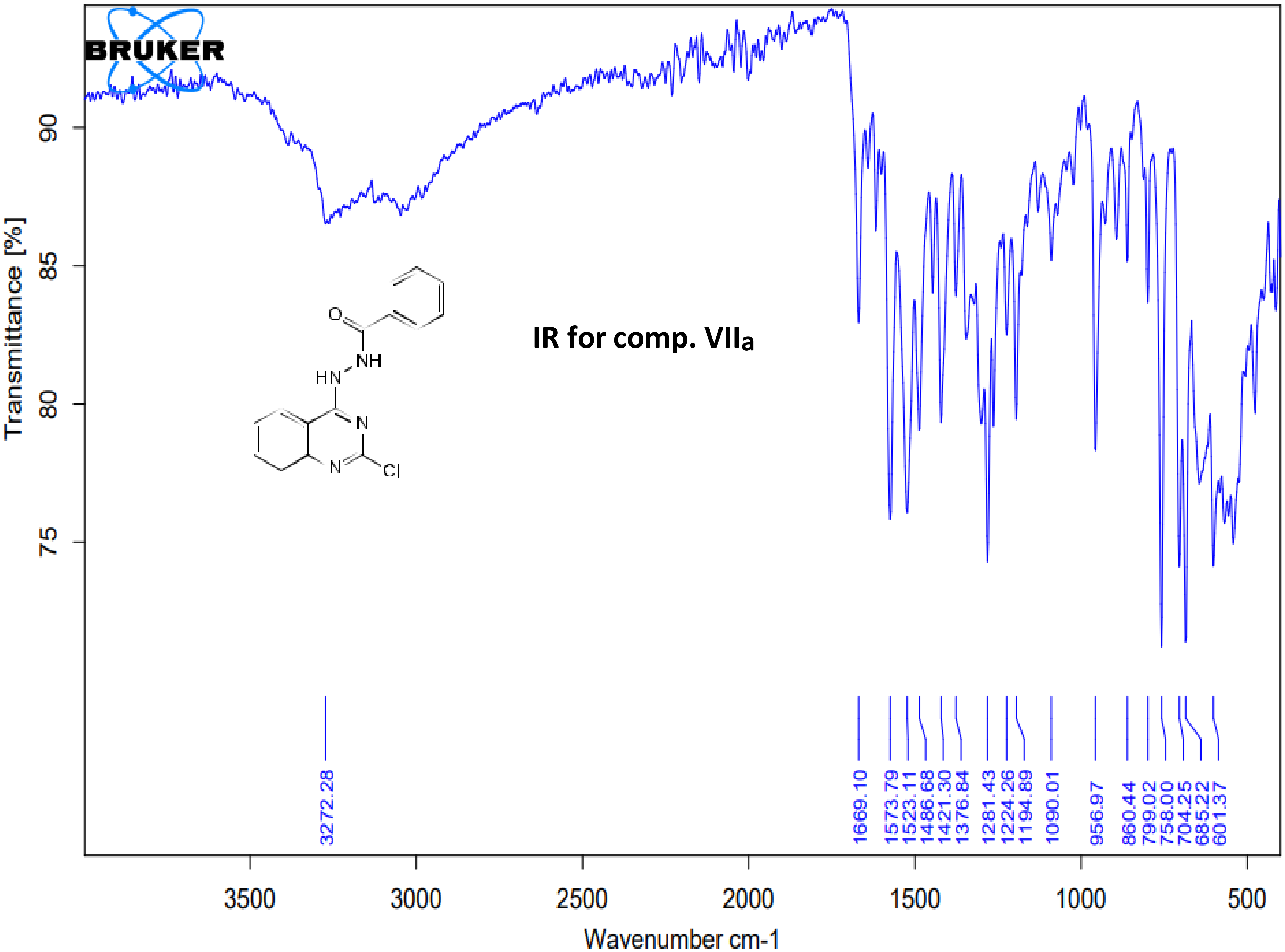
BRUKER
IR for comp. VIIa
O
NH
HN
N
N
Cl
Transmittance [%]
90
85
80
75
3272.28
1669.10
1573.79
1523.11
1486.68
1421.30
1376.84
1281.43
1224.26
1194.89
1090.01
956.97
860.44
799.02
758.00
704.25
685.22
601.37
3500
3000
2500
2000
1500
1000
500
Wavenumber cm-1

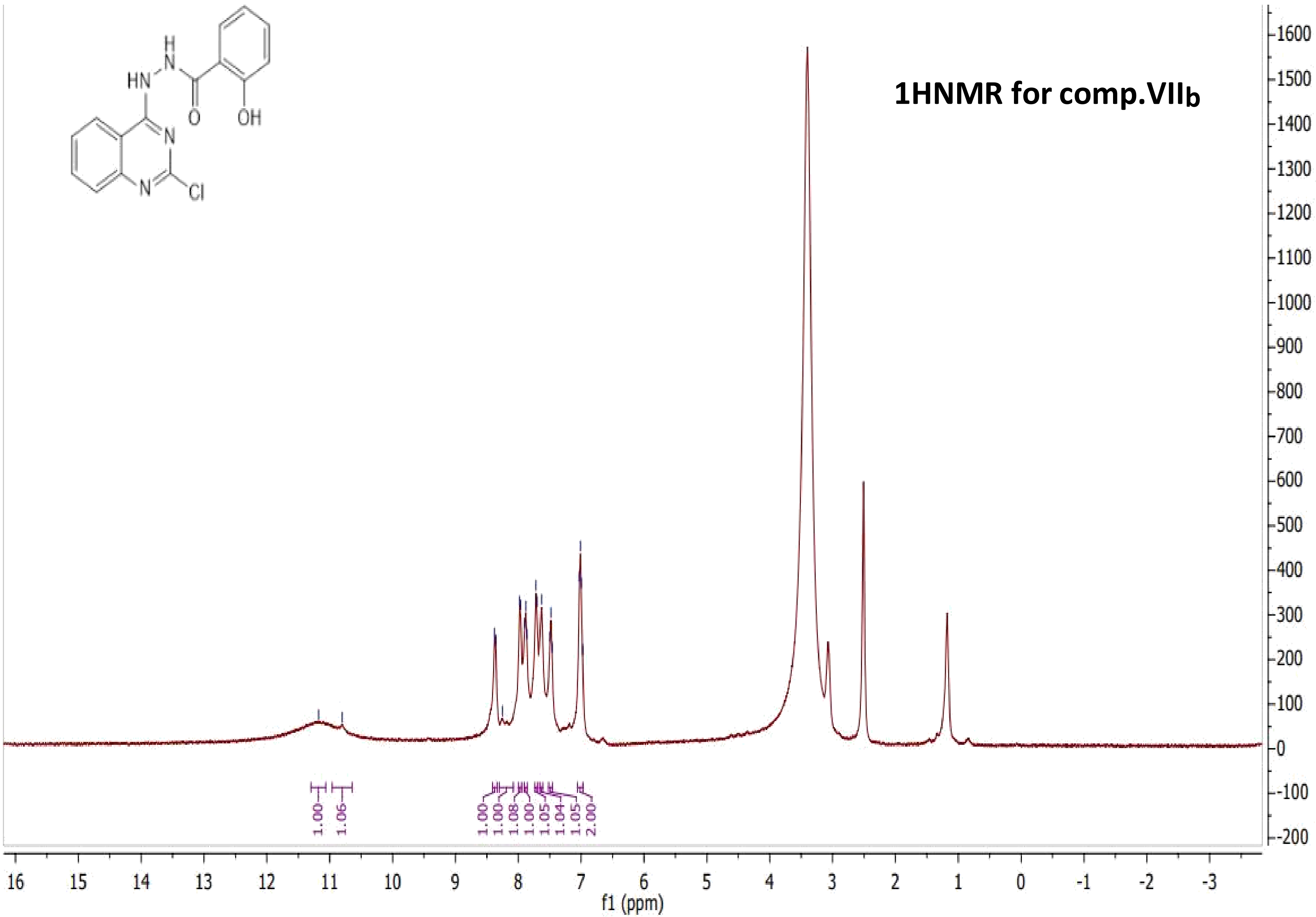

1HNMR for comp.VIIb
HN
N
H
O
OH
N
N
Cl
1.00
1.06
1.00
1.00
1.08
1.00
1.05
1.04
1.05
2.00
16 15 14 13 12 11 10 9 8 7 6 5 4 3 2 1 0 -1 -2 -3
f1 (ppm)
1600 1500 1400 1300 1200 1100 1000 900 800 700 600 500 400 300 200 100 0 -100 -200

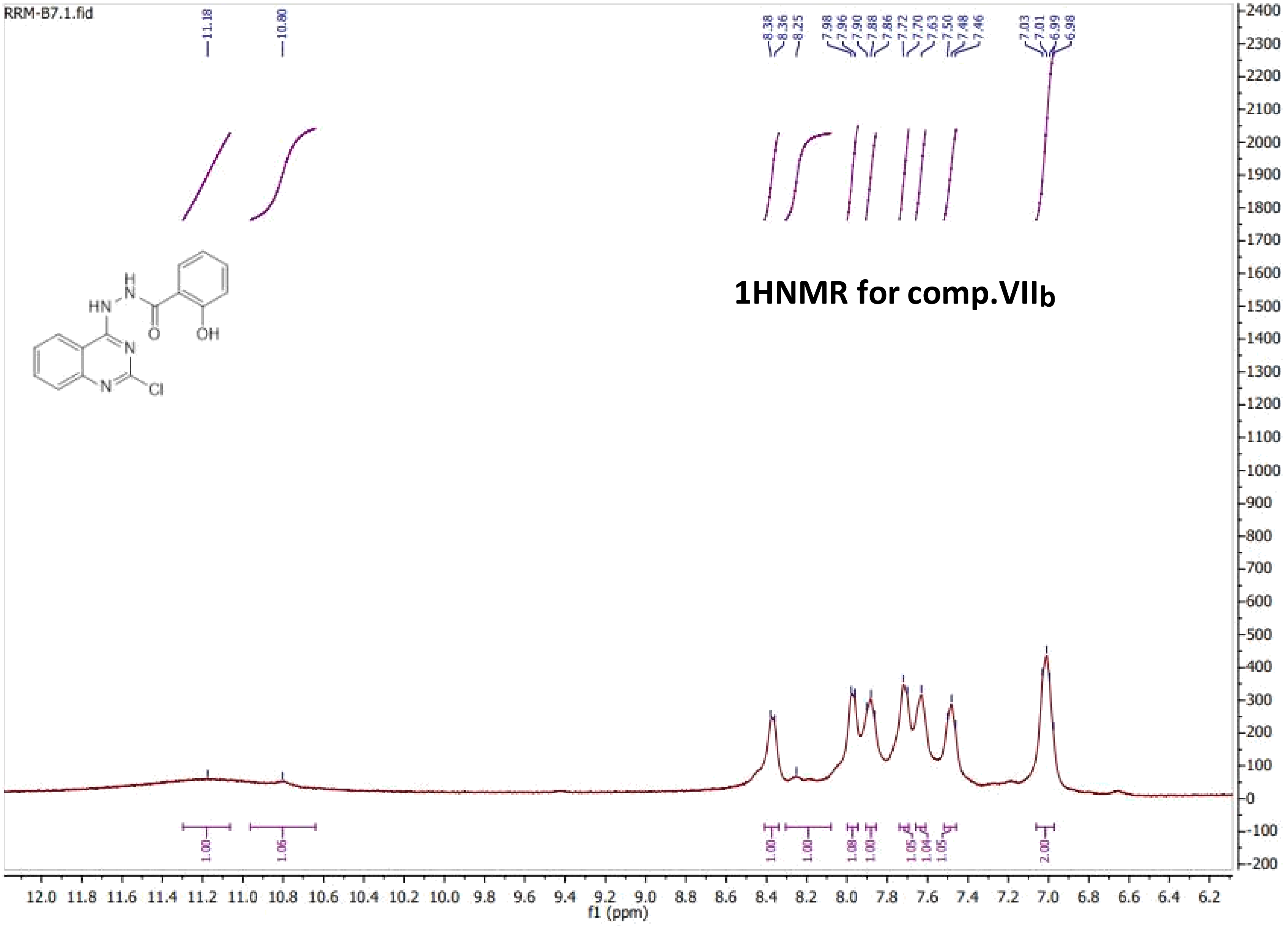
RRM-B7.1.fid
11.18
10.80
8.38
8.36
8.25
7.98
7.96
7.90
7.88
7.86
7.72
7.70
7.63
7.50
7.48
7.46
7.03
7.01
6.99
6.98
1HNMR for comp.VIIb
HN
N
H
O
OH
N
N
Cl
1.00
1.06
1.00
1.00
1.08
1.00
1.05
1.04
1.05
2.00
f1 (ppm)

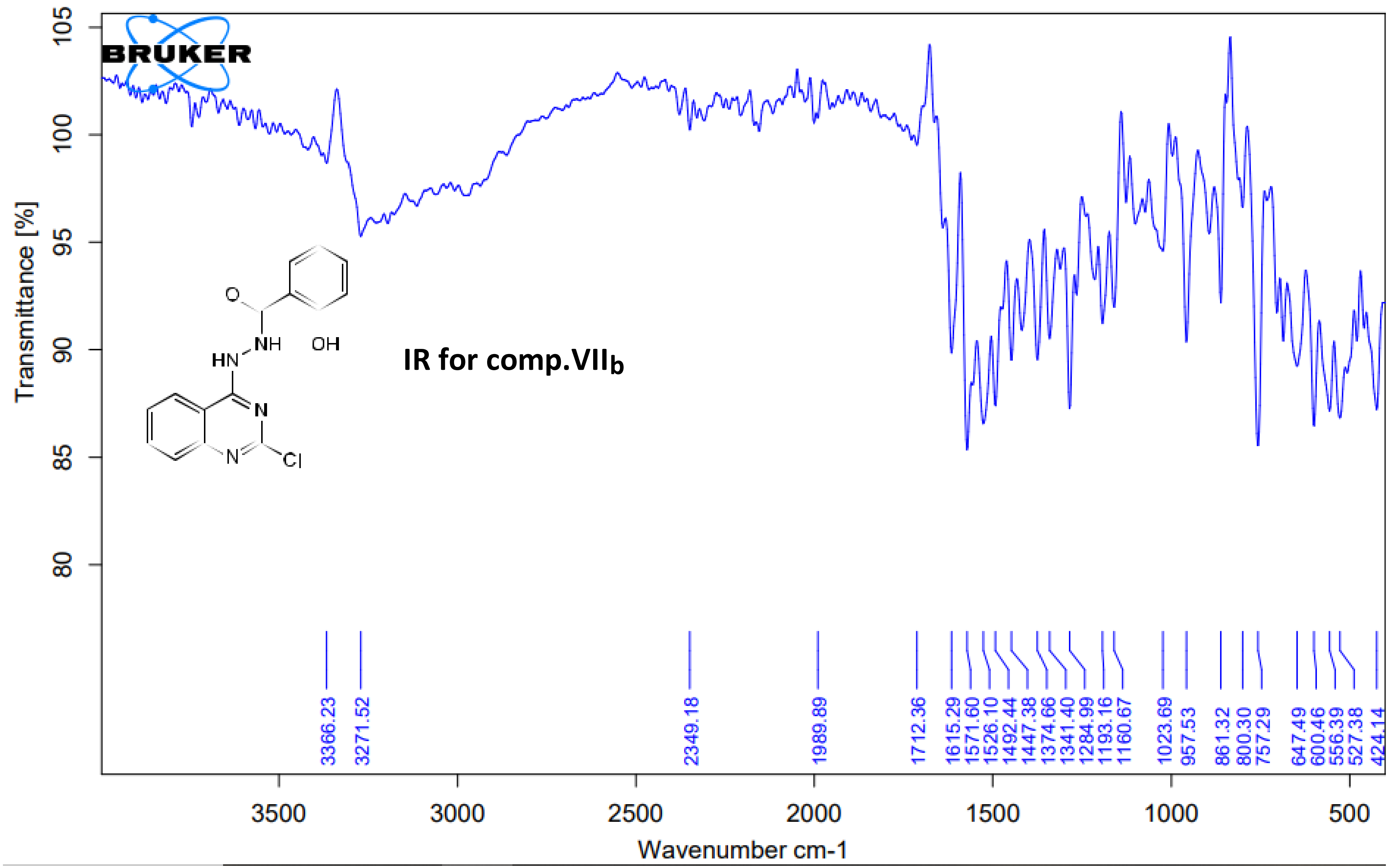
IR for comp.VII$_b$
BRUKER
Transmittance [%]
Wavenumber cm-1
105
100
95
90
85
80
3500
3000
2500
2000
1500
1000
500
3366.23
3271.52
2349.18
1989.89
1712.36
1615.29
1571.60
1526.10
1492.44
1447.38
1374.66
1341.40
1284.99
1193.16
1160.67
1023.69
957.53
861.32
800.30
757.29
647.49
600.46
556.39
527.38
424.14
O
NH
HN
OH
N
N
Cl

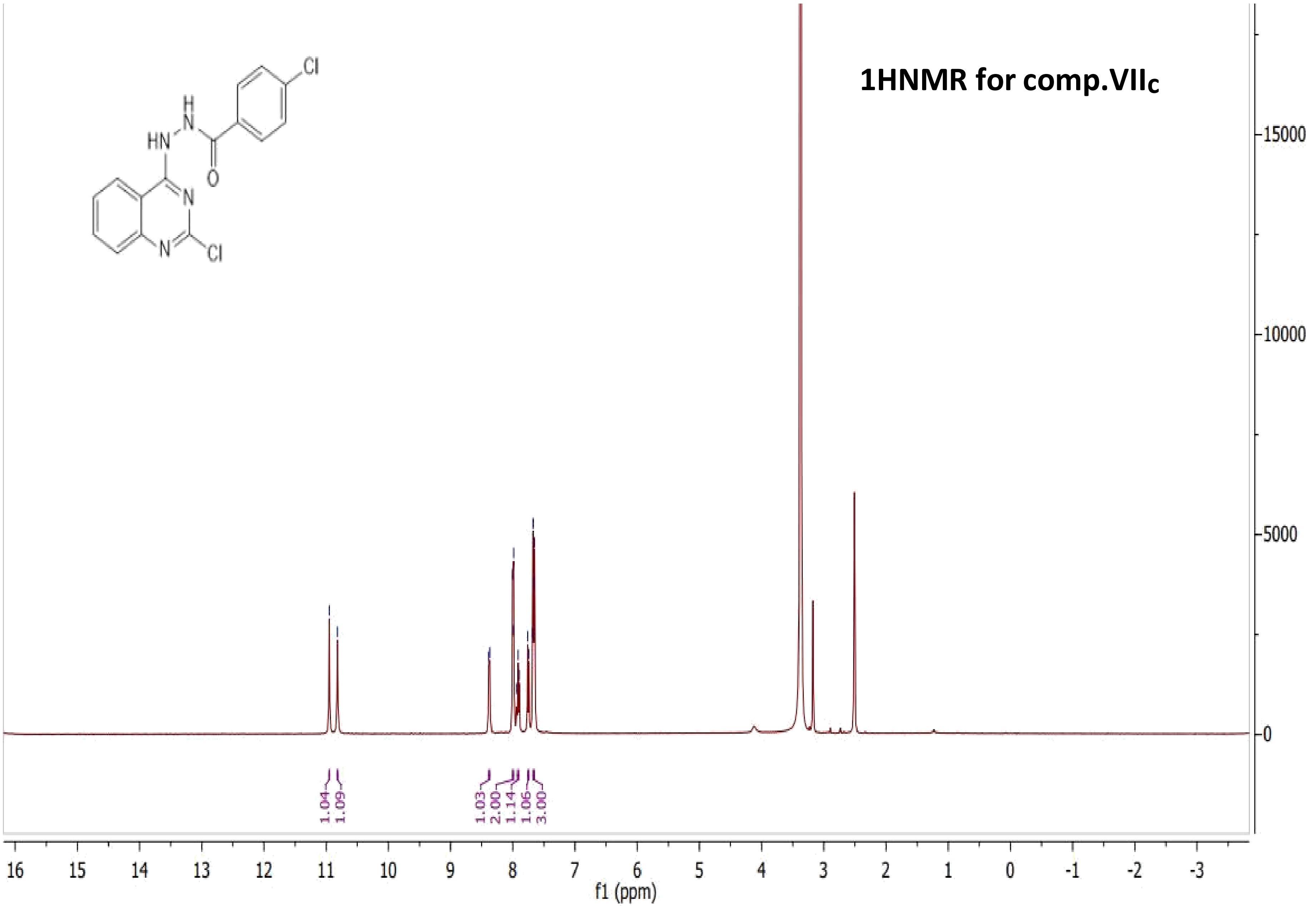
1HNMR for comp.VIIc
Cl
H
N
HN
O
N
N
Cl
15000
10000
5000
0
1.04
1.09
1.03
2.00
1.14
1.06
3.00
16 15 14 13 12 11 10 9 8 7 6 5 4 3 2 1 0 -1 -2 -3
f1 (ppm)

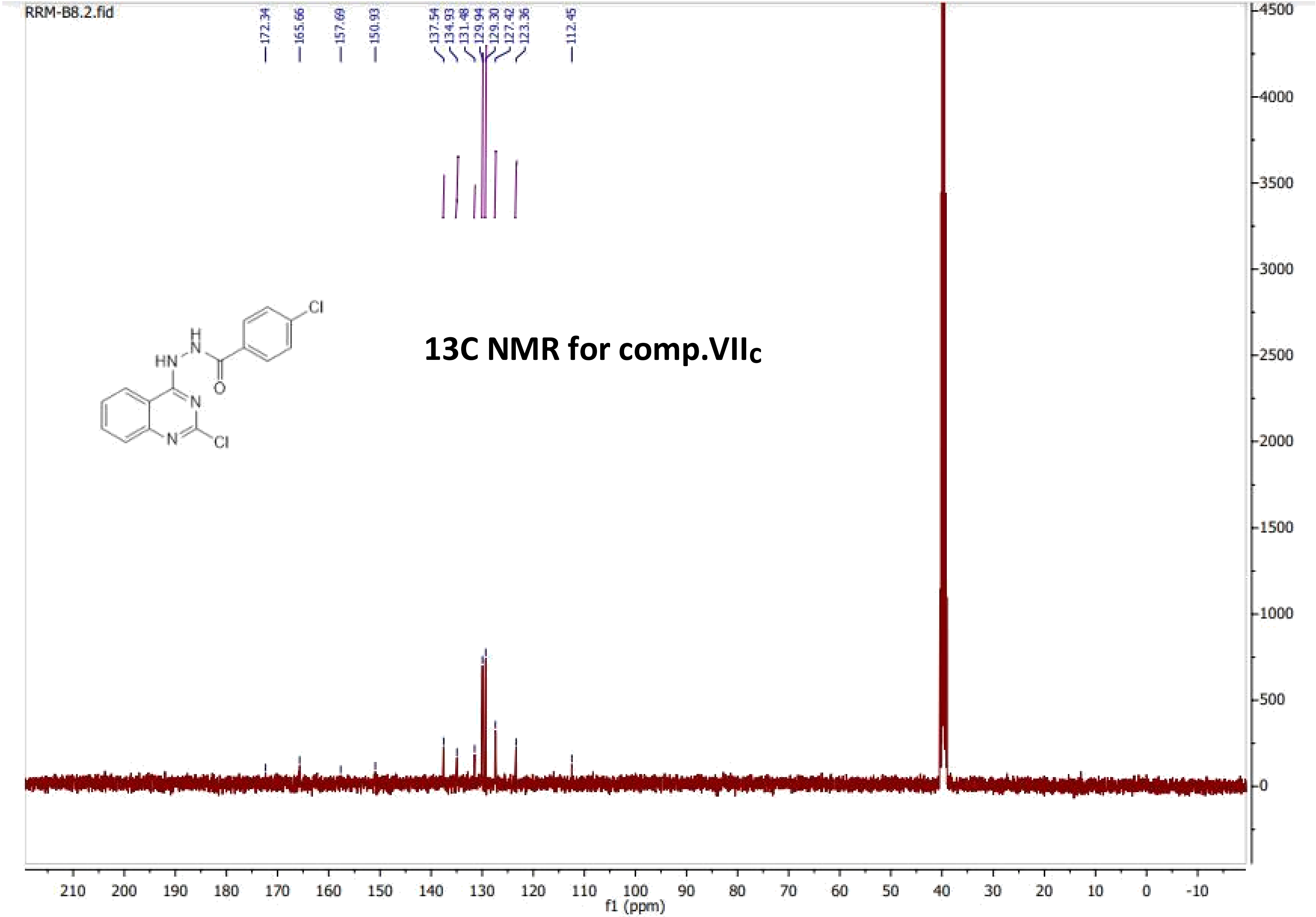

RRM-B8.2.fid
172.34
165.66
157.69
150.93
137.54
134.93
131.48
129.94
129.30
127.42
123.36
112.45
13C NMR for comp.VIIc
HN
N
H
O
Cl
N
N
Cl
f1 (ppm)

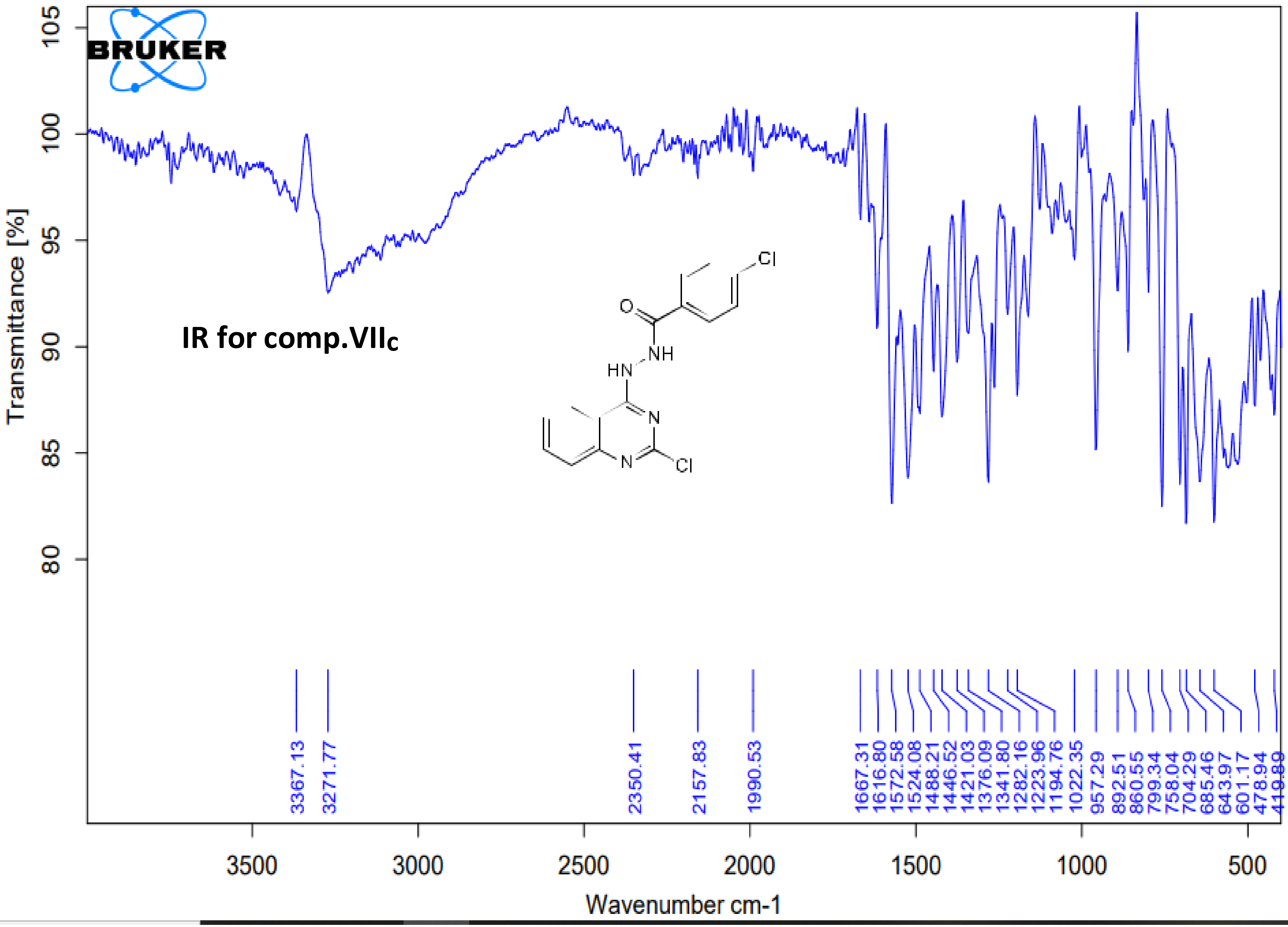
BRUKER
IR for comp.VIIc
Transmittance [%]
105
100
95
90
85
80
O
NH
HN
N
N
Cl
Cl
3367.13
3271.77
2350.41
2157.83
1990.53
1667.31
1616.80
1572.58
1524.08
1488.21
1446.52
1421.03
1376.09
1341.80
1282.16
1223.96
1194.76
1022.35
957.29
892.51
860.55
799.34
758.04
704.29
685.46
643.97
601.17
478.94
419.89
3500
3000
2500
2000
1500
1000
500
Wavenumber cm-1

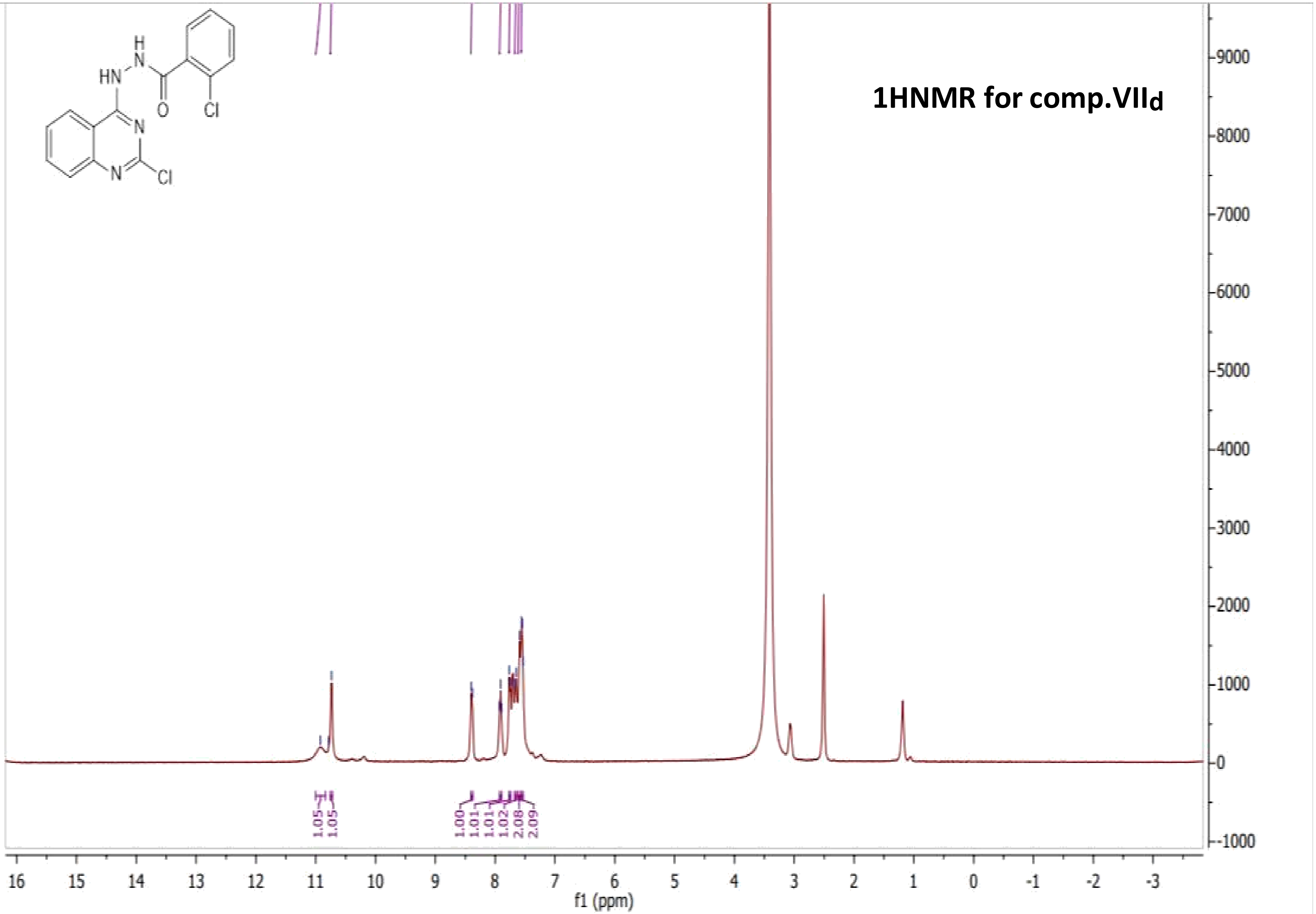

1HNMR for comp.VIId
HN
H
N
O
Cl
N
N
Cl
9000
8000
7000
6000
5000
4000
3000
2000
1000
0
-1000
1.05
1.05
1.00
1.01
1.01
1.02
2.08
2.09
16
15
14
13
12
11
10
9
8
7
6
5
4
3
2
1
0
-1
-2
-3
f1 (ppm)

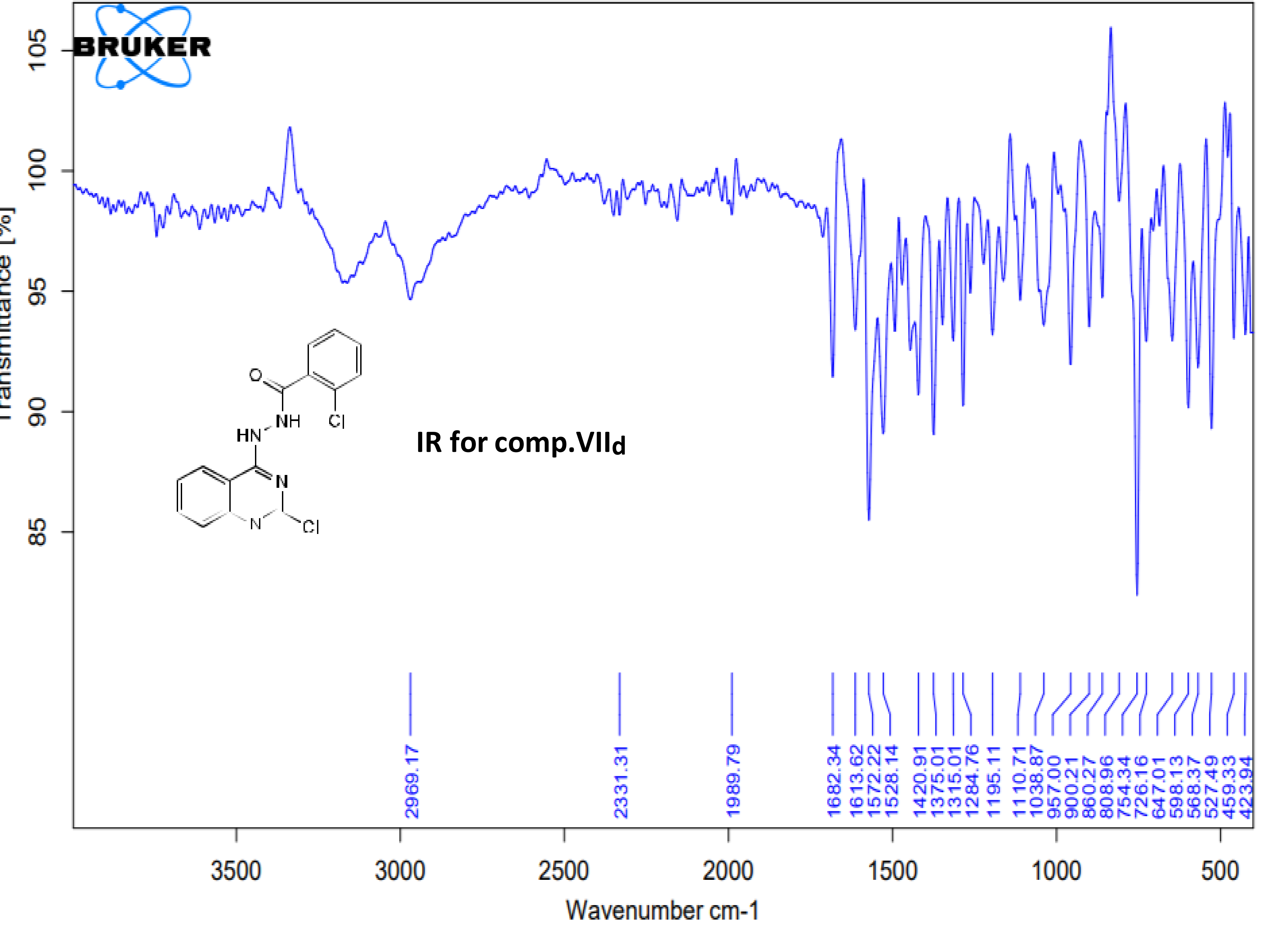

BRUKER
IR for comp.VIId
Transmittance [%]
105
100
95
90
85
HN
NH
O
Cl
N
N
Cl
2969.17
2331.31
1989.79
1682.34
1613.62
1572.22
1528.14
1420.91
1375.01
1315.01
1284.76
1195.11
1110.71
1038.87
957.00
900.21
860.27
808.96
754.34
726.16
647.01
598.13
568.37
527.49
459.33
423.94
3500
3000
2500
2000
1500
1000
500
Wavenumber cm-1

1HNMR for comp .XI

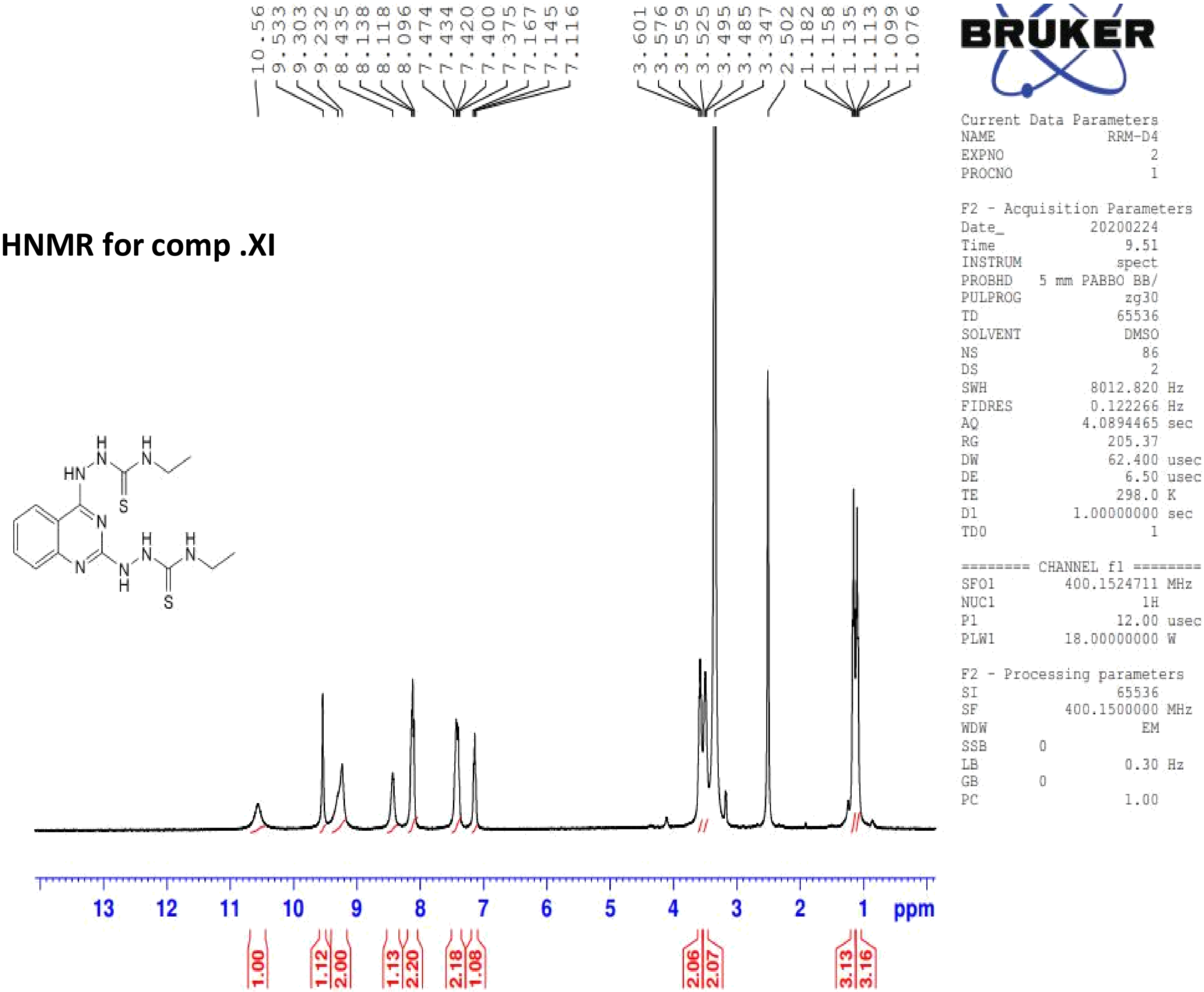

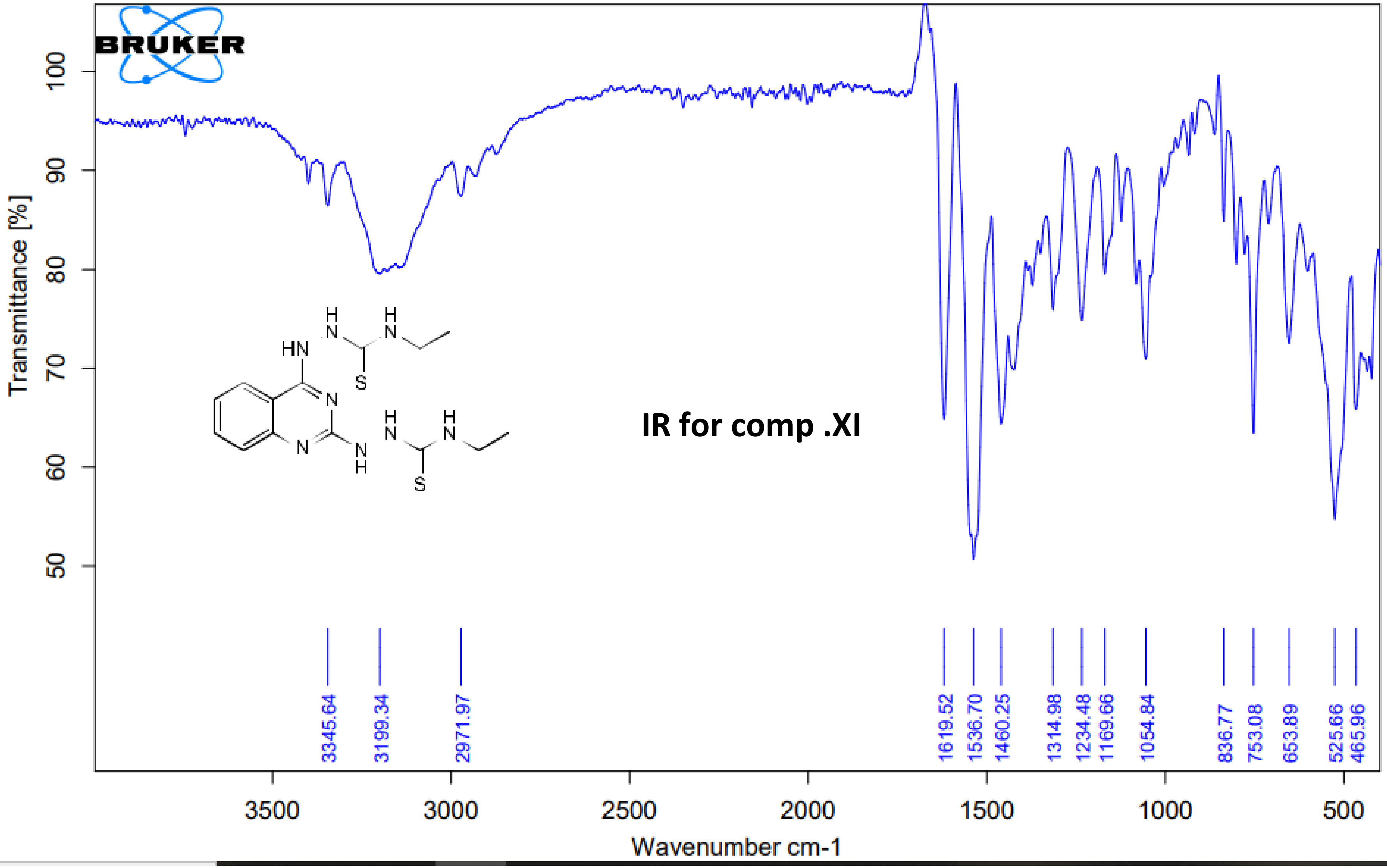
IR for comp .XI
Transmittance [%]
Wavenumber cm-1
100
90
80
70
60
50
3500
3000
2500
2000
1500
1000
500
3345.64
3199.34
2971.97
1619.52
1536.70
1460.25
1314.98
1234.48
1169.66
1054.84
836.77
753.08
653.89
525.66
465.96
HN
H
N
S
N
N
NH

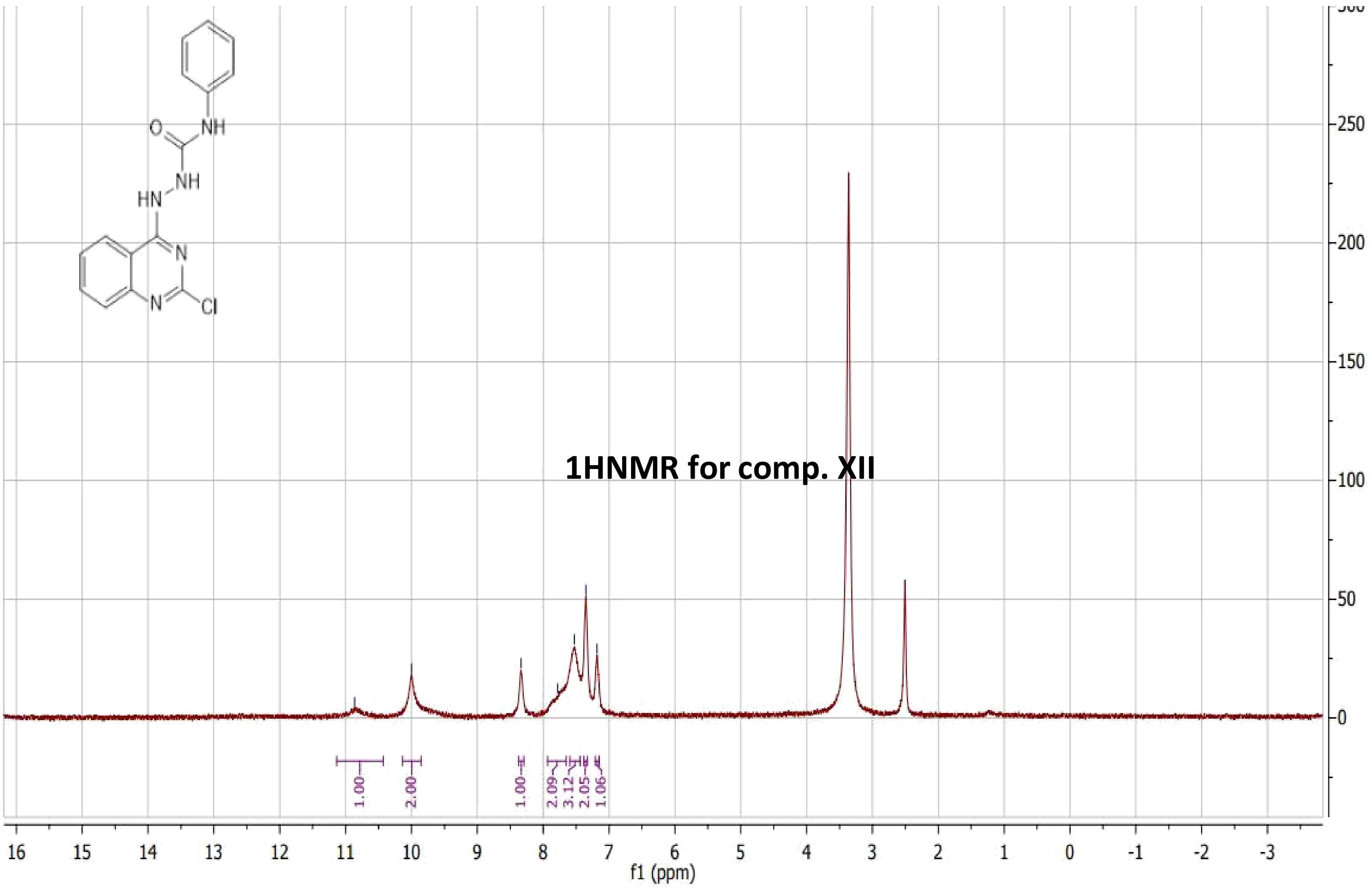

1HNMR for comp. XII
O
NH
HN
NH
N
N
Cl
1.00
2.00
1.00
2.09
3.12
2.05
1.06
f1 (ppm)

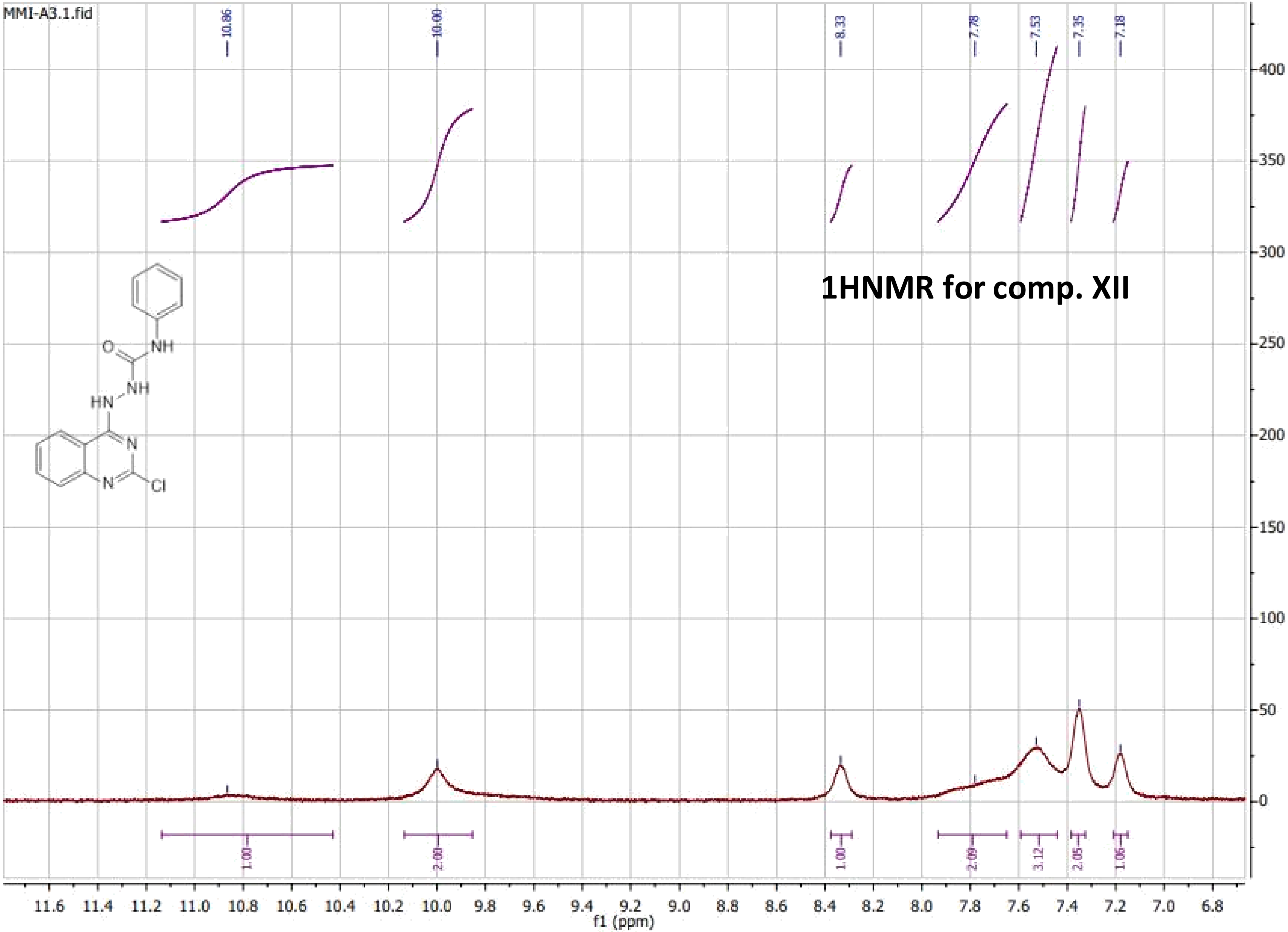
MMI-A3.1.fid
10.86
10.00
8.33
7.78
7.53
7.35
7.18
1HNMR for comp. XII
O
NH
NH
HN
N
N
Cl
1.00
2.00
1.00
2.09
3.12
2.05
1.06
f1 (ppm)

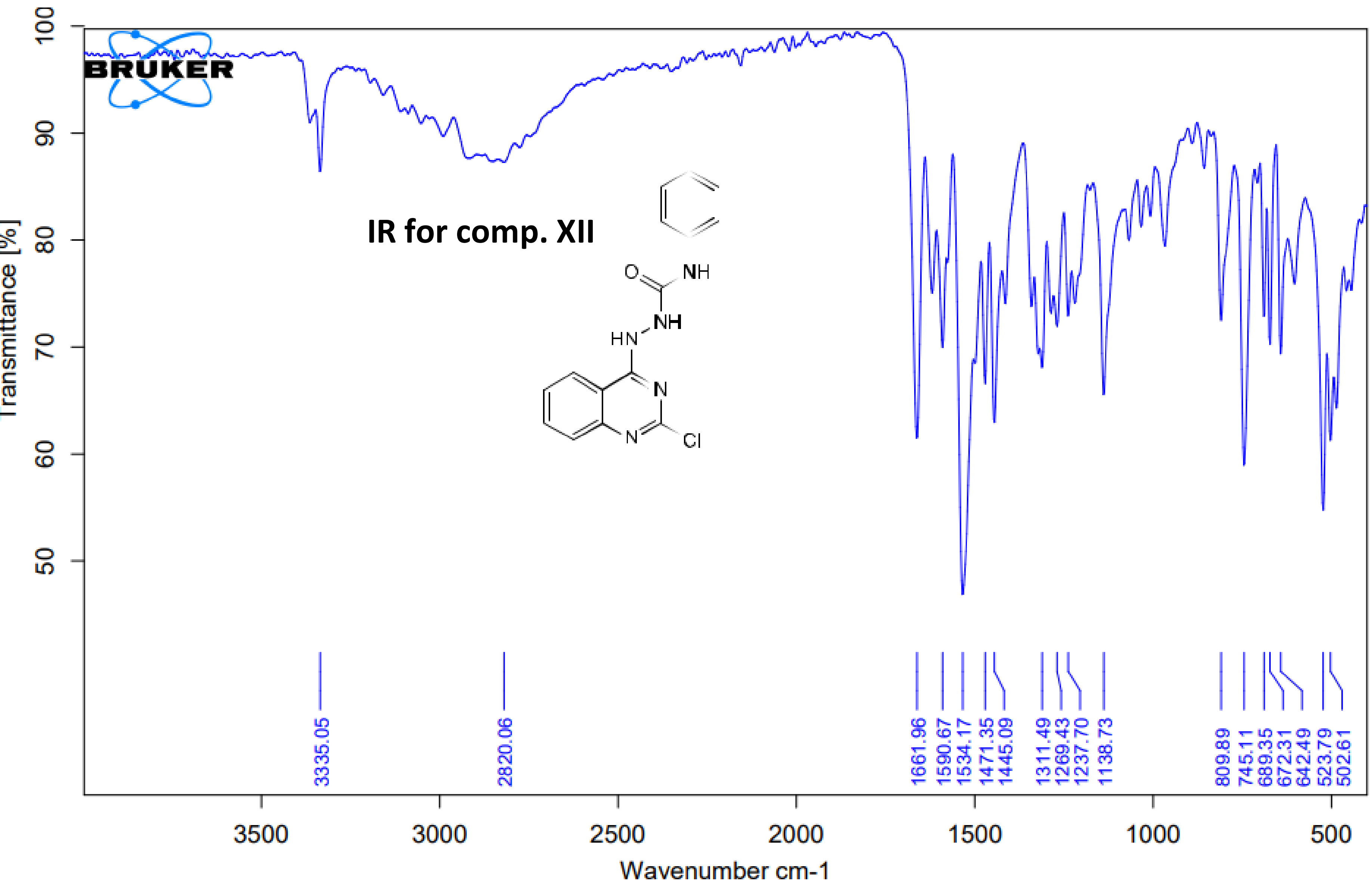

IR for comp. XII
BRUKER
Transmittance [%]
Wavenumber cm-1
100
90
80
70
60
50
3500
3000
2500
2000
1500
1000
500
3335.05
2820.06
1661.96
1590.67
1534.17
1471.35
1445.09
1311.49
1269.43
1237.70
1138.73
809.89
745.11
689.35
672.31
642.49
523.79
502.61
O
NH
NH
HN
N
N
Cl

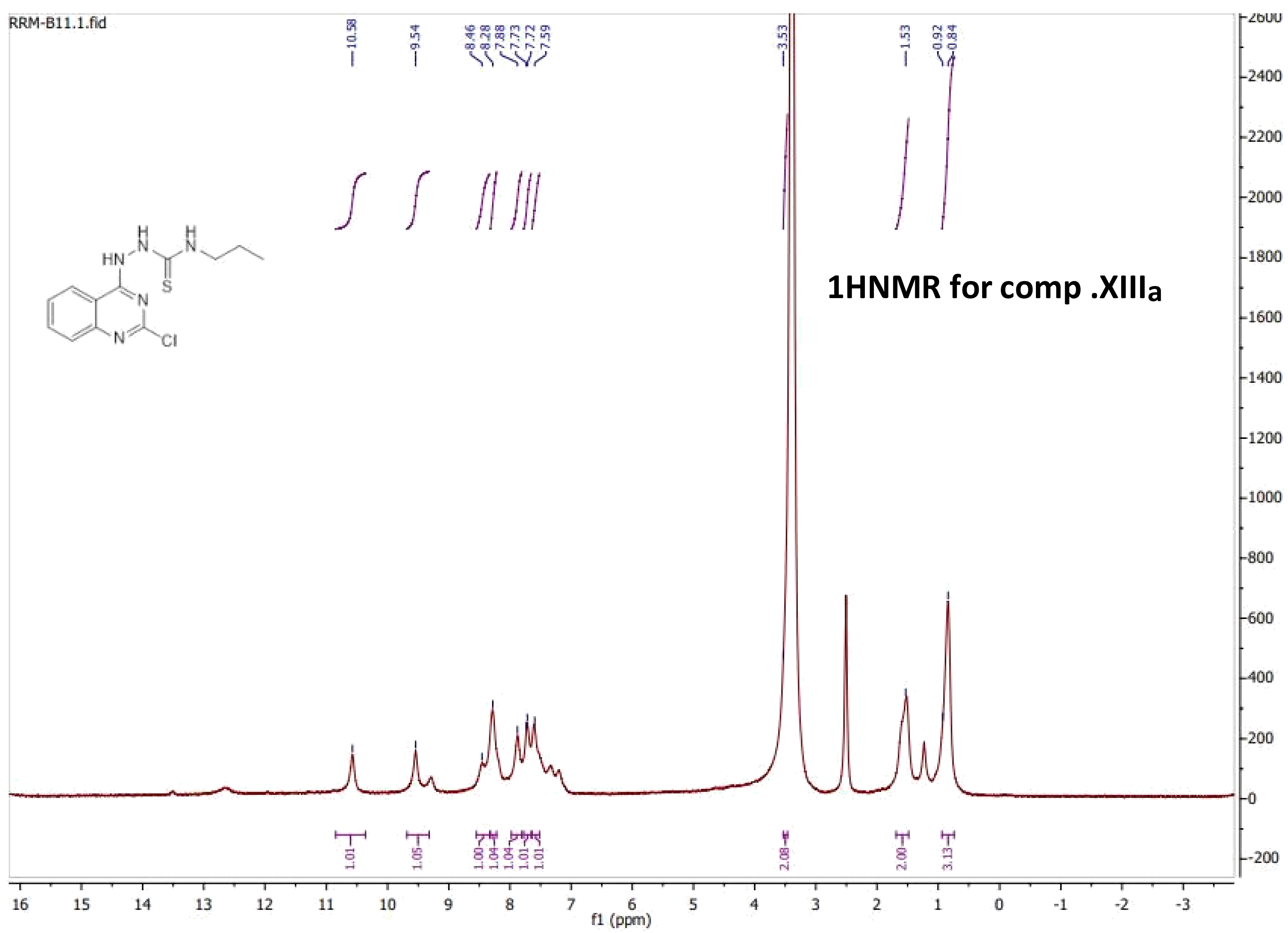

RRM-B11.1.fid
10.58
9.54
8.46
8.28
7.88
7.73
7.72
7.59
3.53
1.53
0.92
0.84
1HNMR for comp .XIIIa
HN
N
H
N
H
S
N
N
Cl
1.01
1.05
1.00
1.04
1.04
1.01
1.01
2.08
2.00
3.13
f1 (ppm)

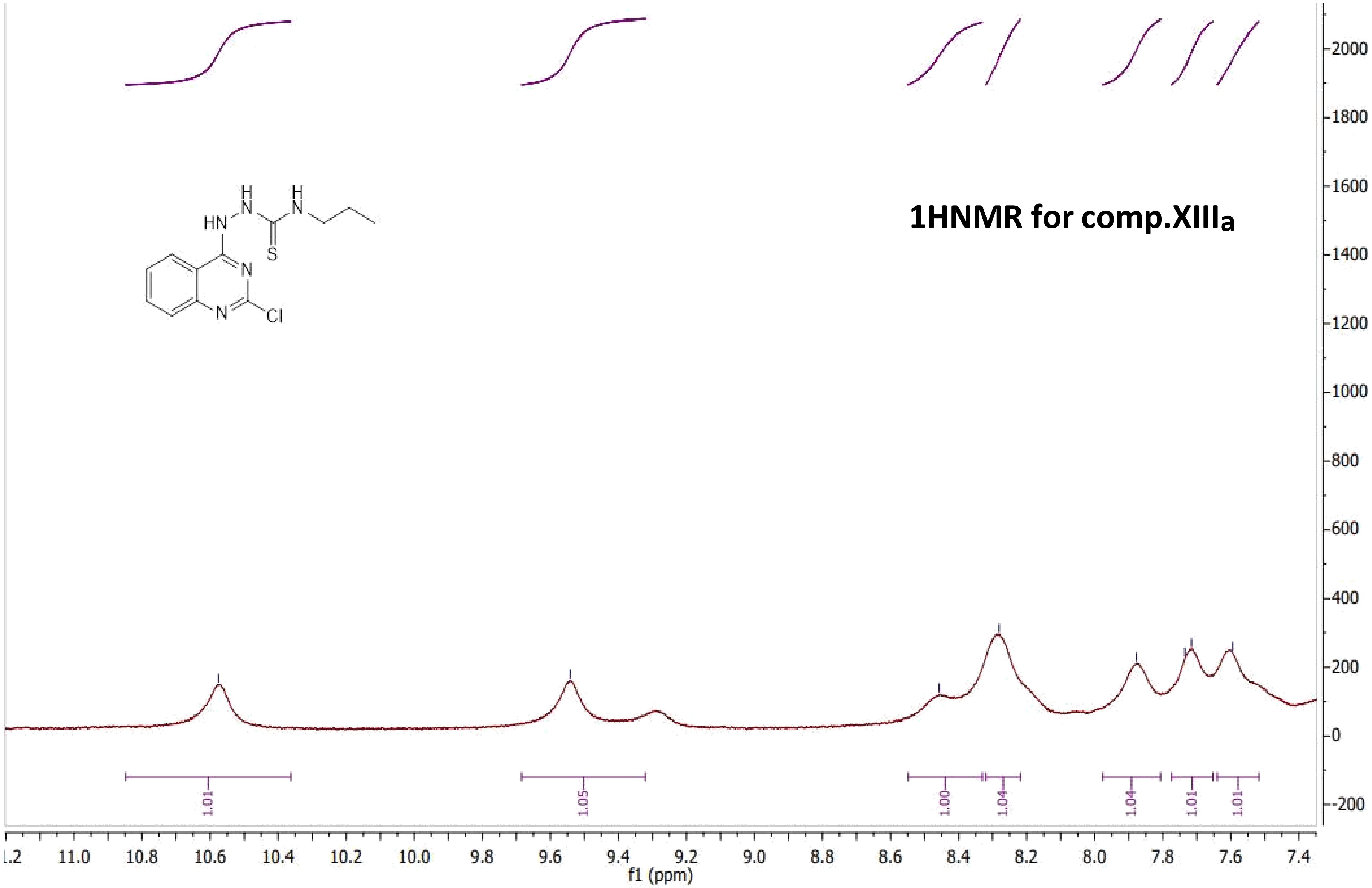
1HNMR for comp.XIII$_a$
HN
H
N
H
N
S
N
N
Cl
1.01
1.05
1.00
1.04
1.04
1.01
1.01
f1 (ppm)
11.0
10.8
10.6
10.4
10.2
10.0
9.8
9.6
9.4
9.2
9.0
8.8
8.6
8.4
8.2
8.0
7.8
7.6
7.4
2000
1800
1600
1400
1200
1000
800
600
400
200
0
-200

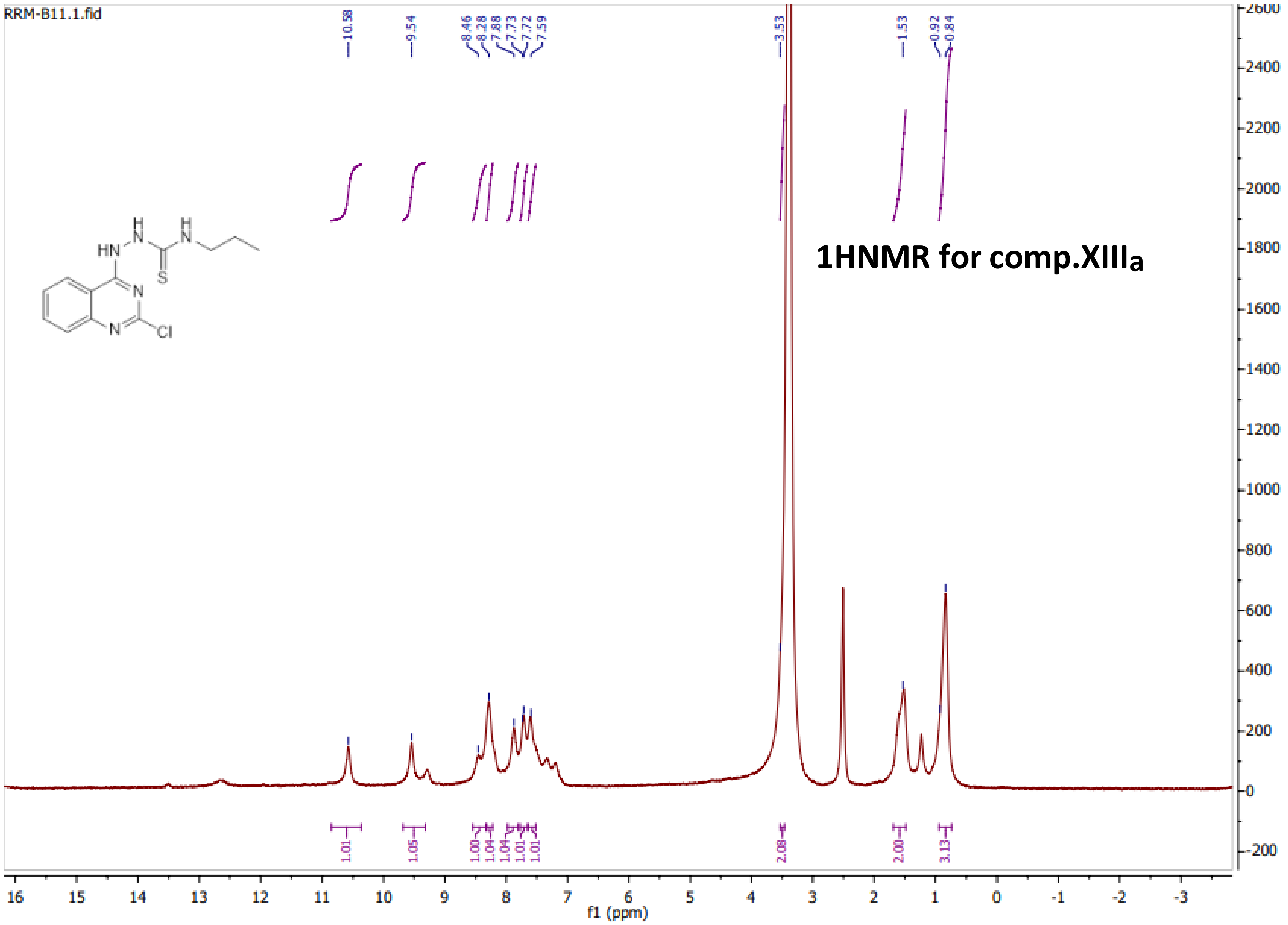

RRM-B11.1.fid
10.58
9.54
8.46
8.28
7.88
7.73
7.72
7.59
3.53
1.53
0.92
0.84
1HNMR for comp.XIIIa
HN
H
N
H
N
S
N
N
Cl
1.01
1.05
1.00
1.04
1.04
1.01
1.01
2.08
2.00
3.13
f1 (ppm)

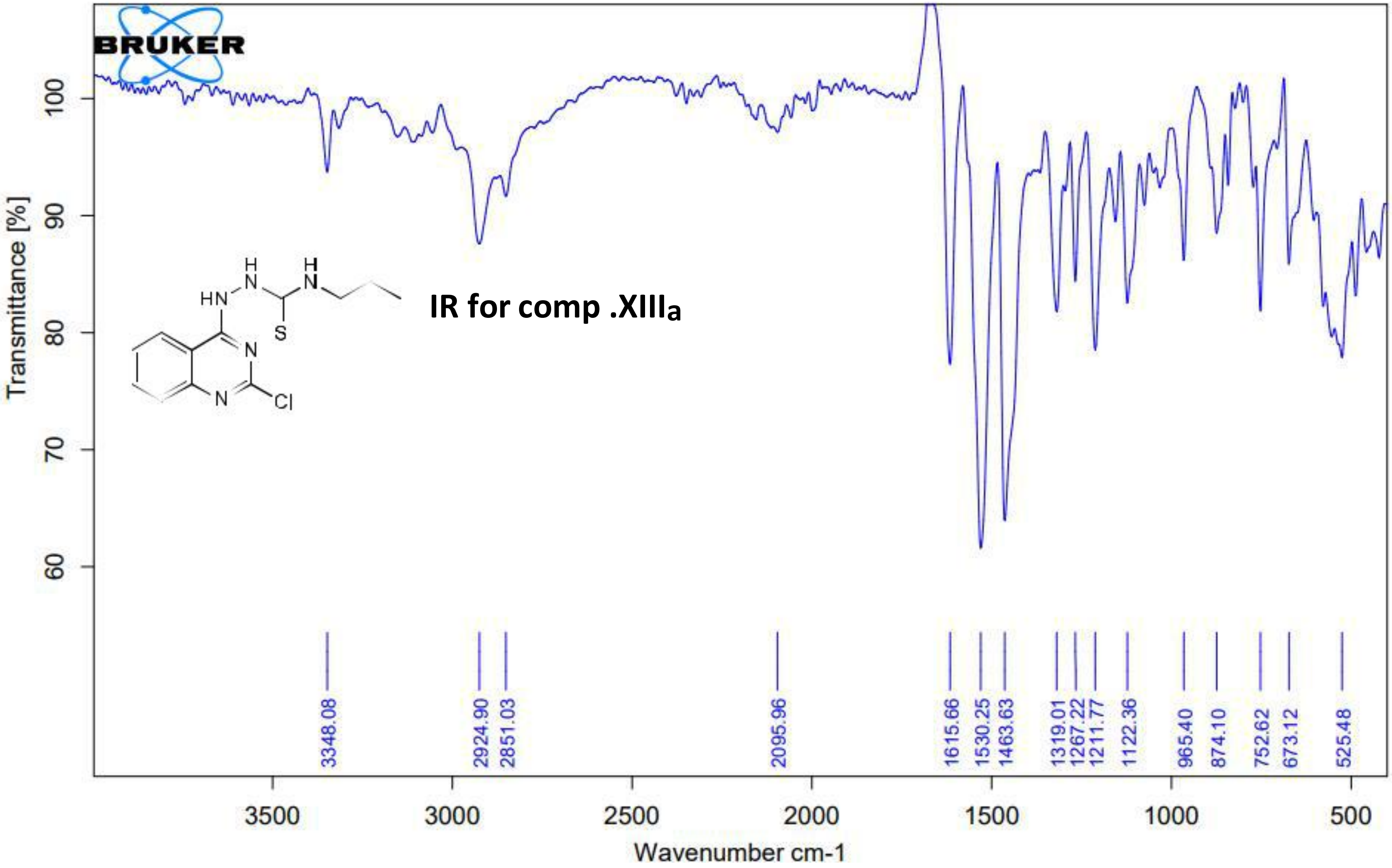
BRUKER
IR for comp .XIIIa
Transmittance [%]
100
90
80
70
60
3348.08
2924.90
2851.03
2095.96
1615.66
1530.25
1463.63
1319.01
1267.22
1211.77
1122.36
965.40
874.10
752.62
673.12
525.48
3500
3000
2500
2000
1500
1000
500
Wavenumber cm-1
HN
H
N
H
N
S
N
N
Cl

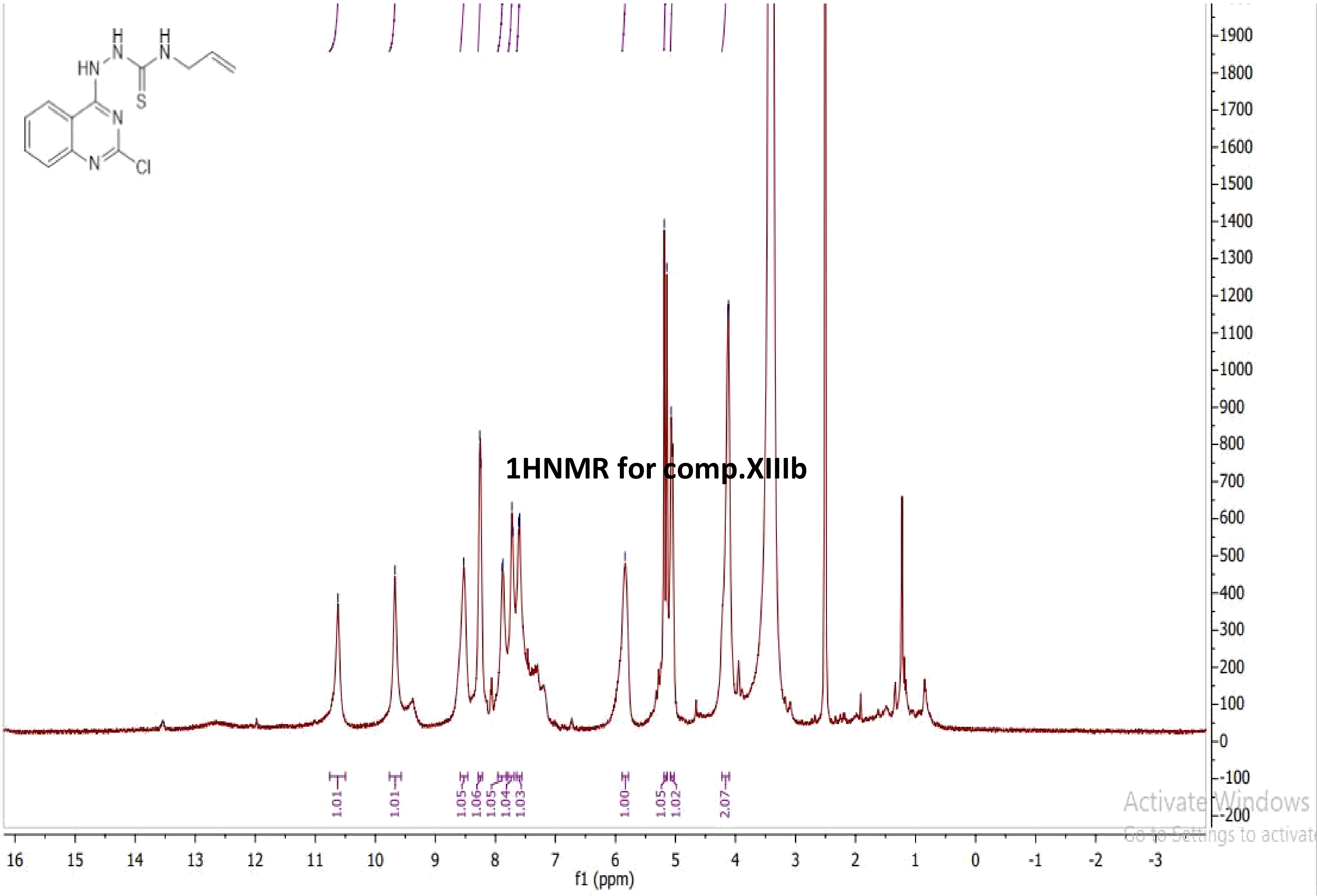

1HNMR for comp.XIIIb

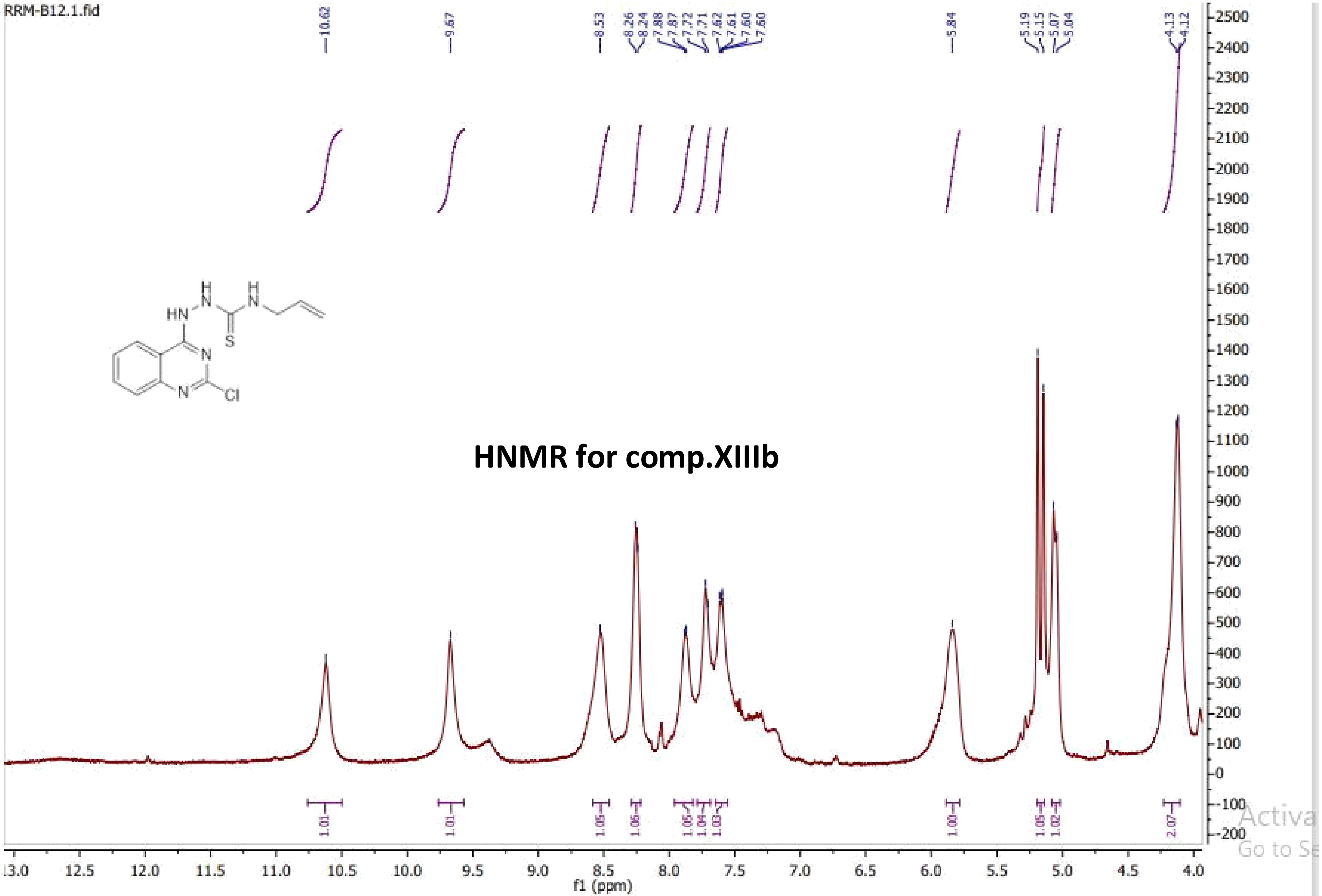

HNMR for comp.XIIIb

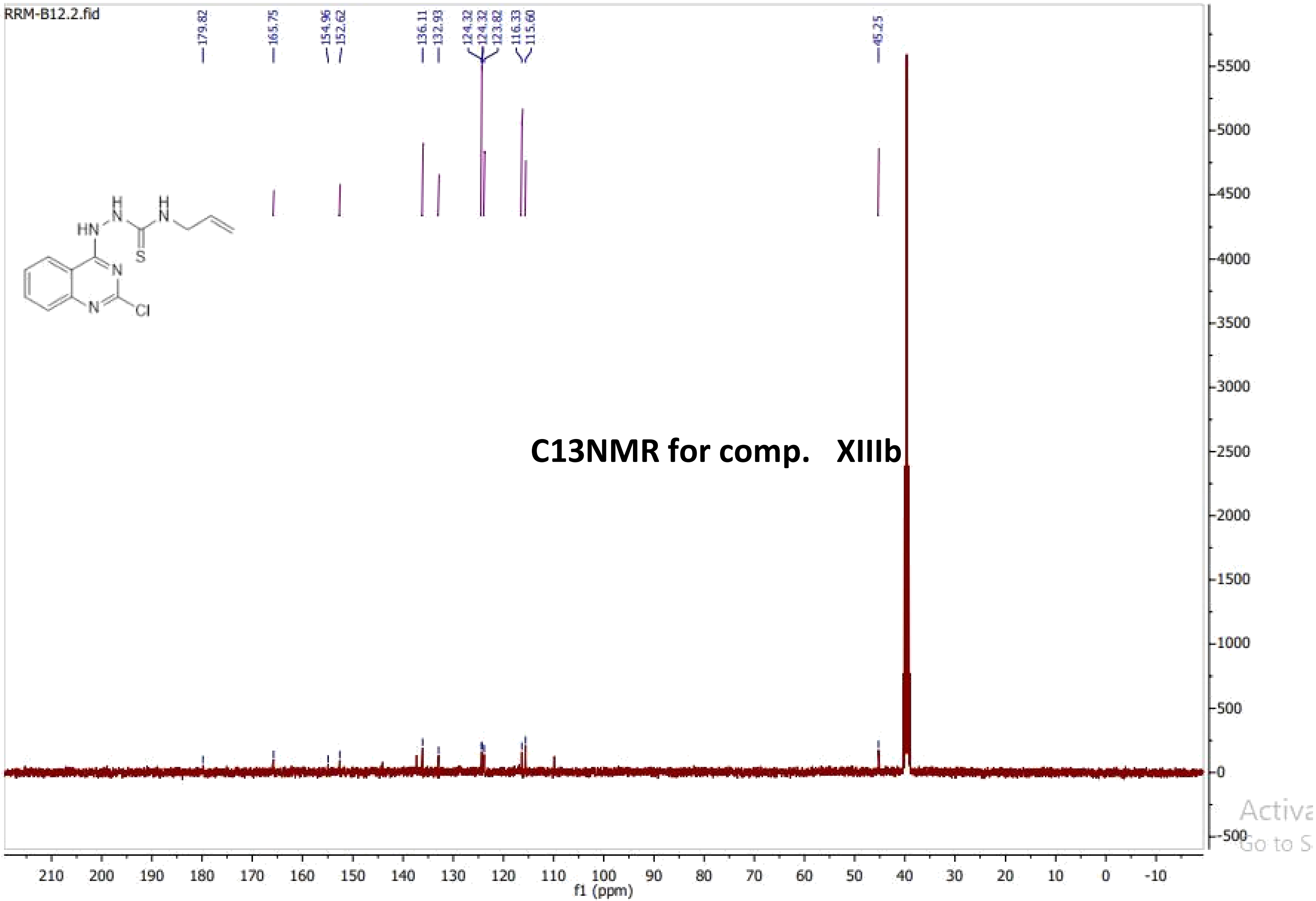
RRM-B12.2.fid
179.82
165.75
154.96
152.62
136.11
132.93
124.32
124.32
123.82
116.33
115.60
45.25
HN
H
N
H
N
S
N
N
Cl
C13NMR for comp. XIIIb
f1 (ppm)
210 200 190 180 170 160 150 140 130 120 110 100 90 80 70 60 50 40 30 20 10 0 -10
5500 5000 4500 4000 3500 3000 2500 2000 1500 1000 500 0 -500

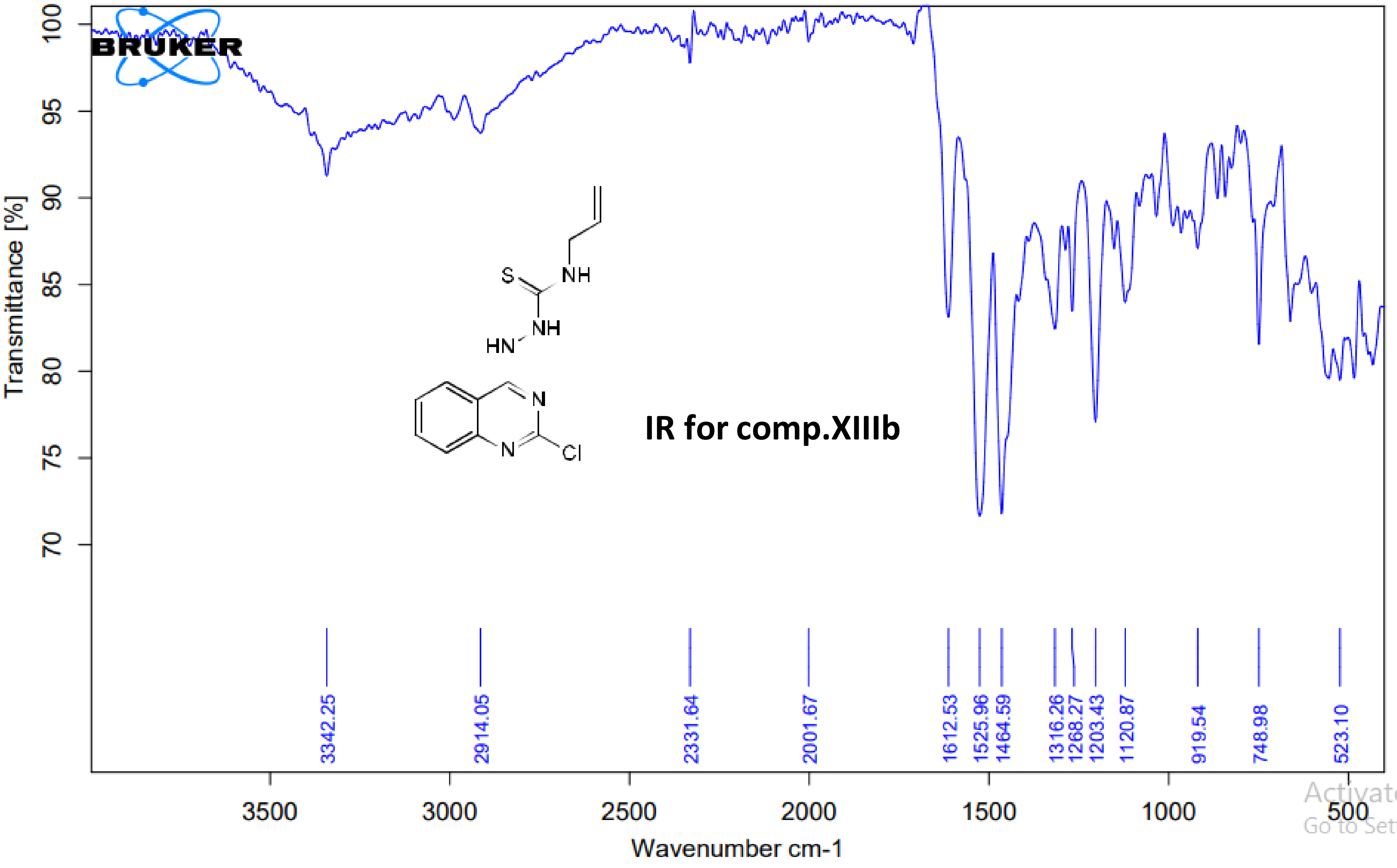

Activate Windo
Go to Settings to ac

1HNMR for comp. XIII$_d$

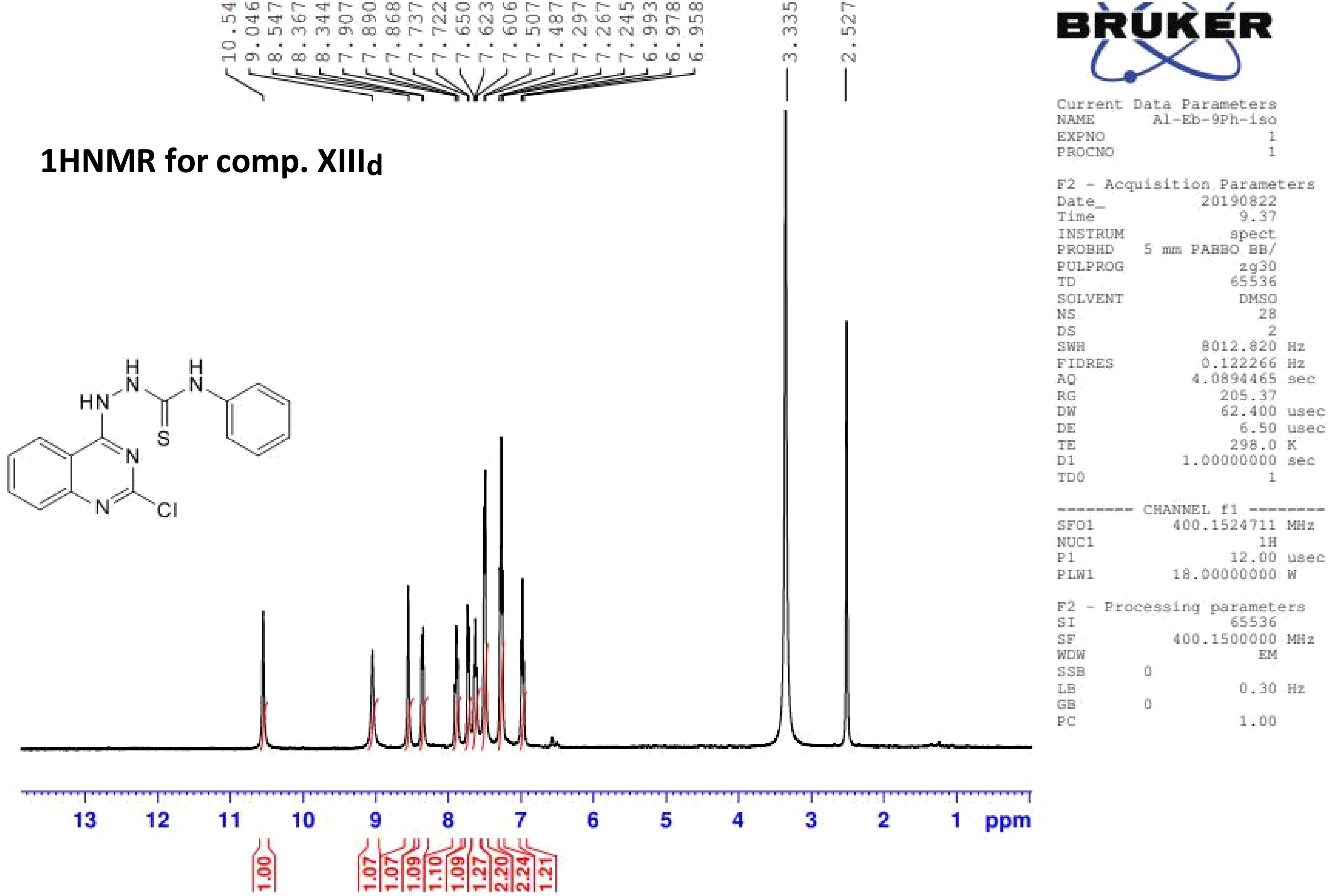

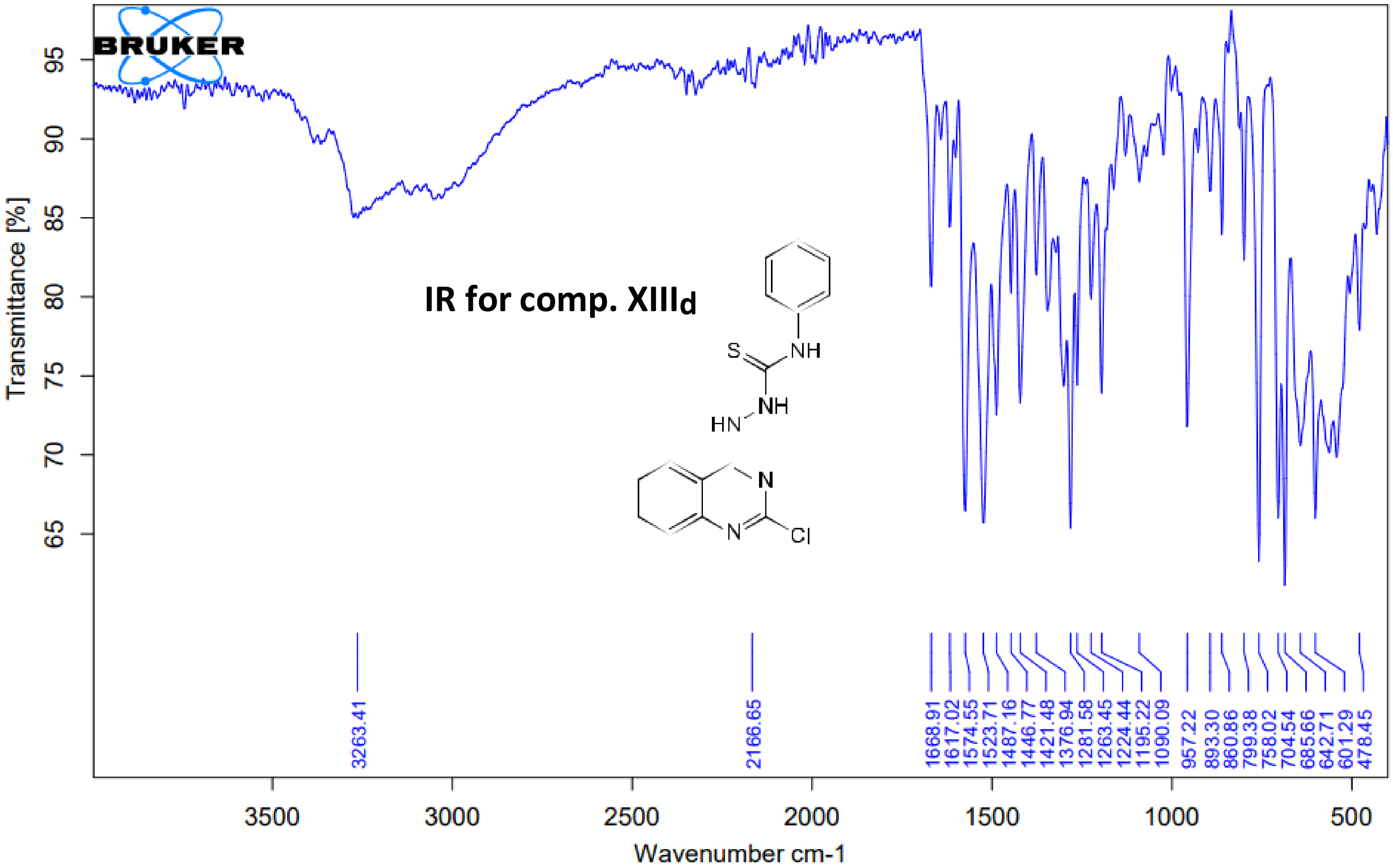
IR for comp. XIIId
S
NH
NH
HN
N
N
Cl
Transmittance [%]
95
90
85
80
75
70
65
3263.41
2166.65
1668.91
1617.02
1574.55
1523.71
1487.16
1446.77
1421.48
1376.94
1281.58
1263.45
1224.44
1195.22
1090.09
957.22
893.30
860.86
799.38
758.02
704.54
685.66
642.71
601.29
478.45
3500
3000
2500
2000
1500
1000
500
Wavenumber cm-1

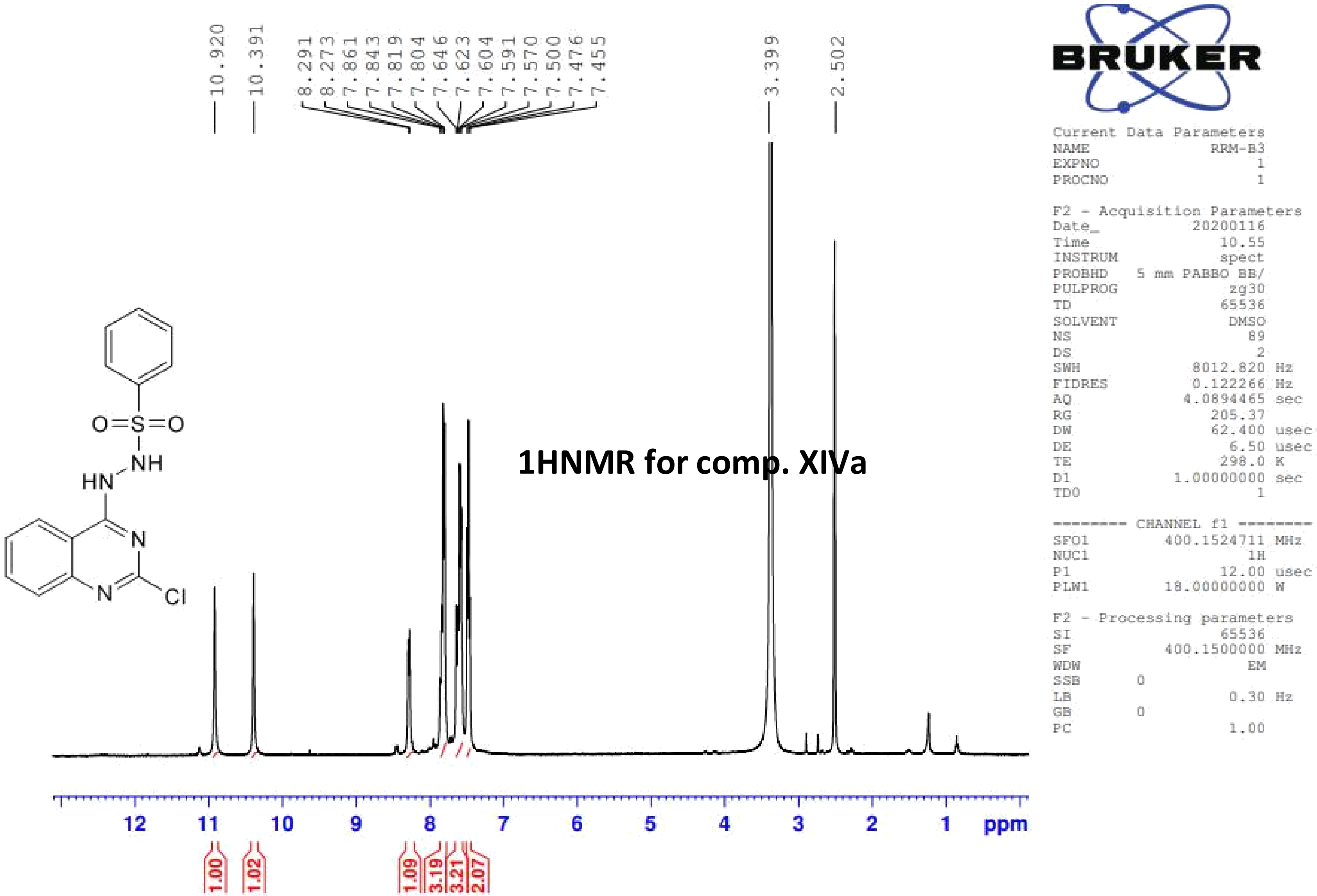

1HNMR for comp. XIVa

Activate Wi
Go to Settings t

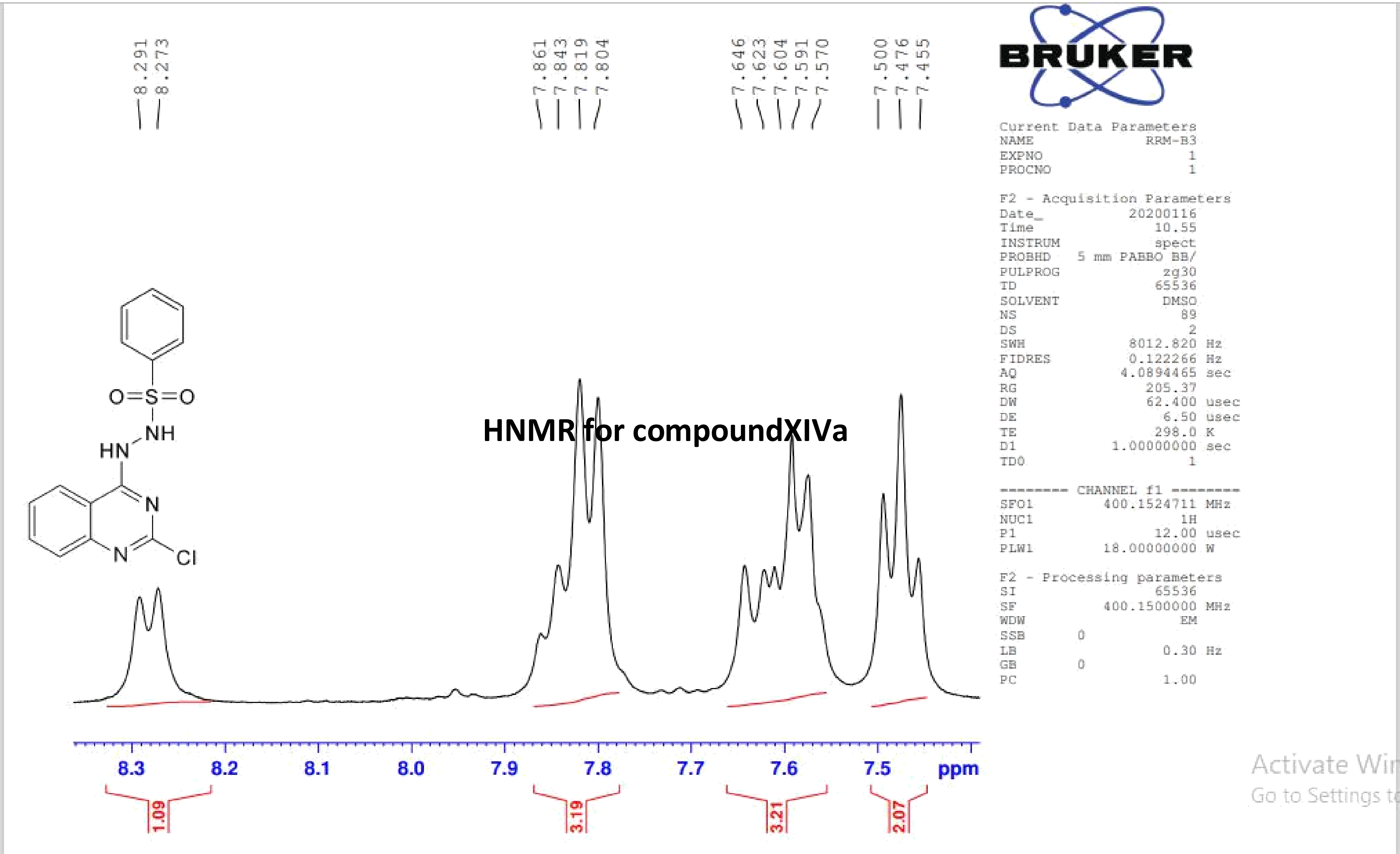

HNMR for compoundXIVa

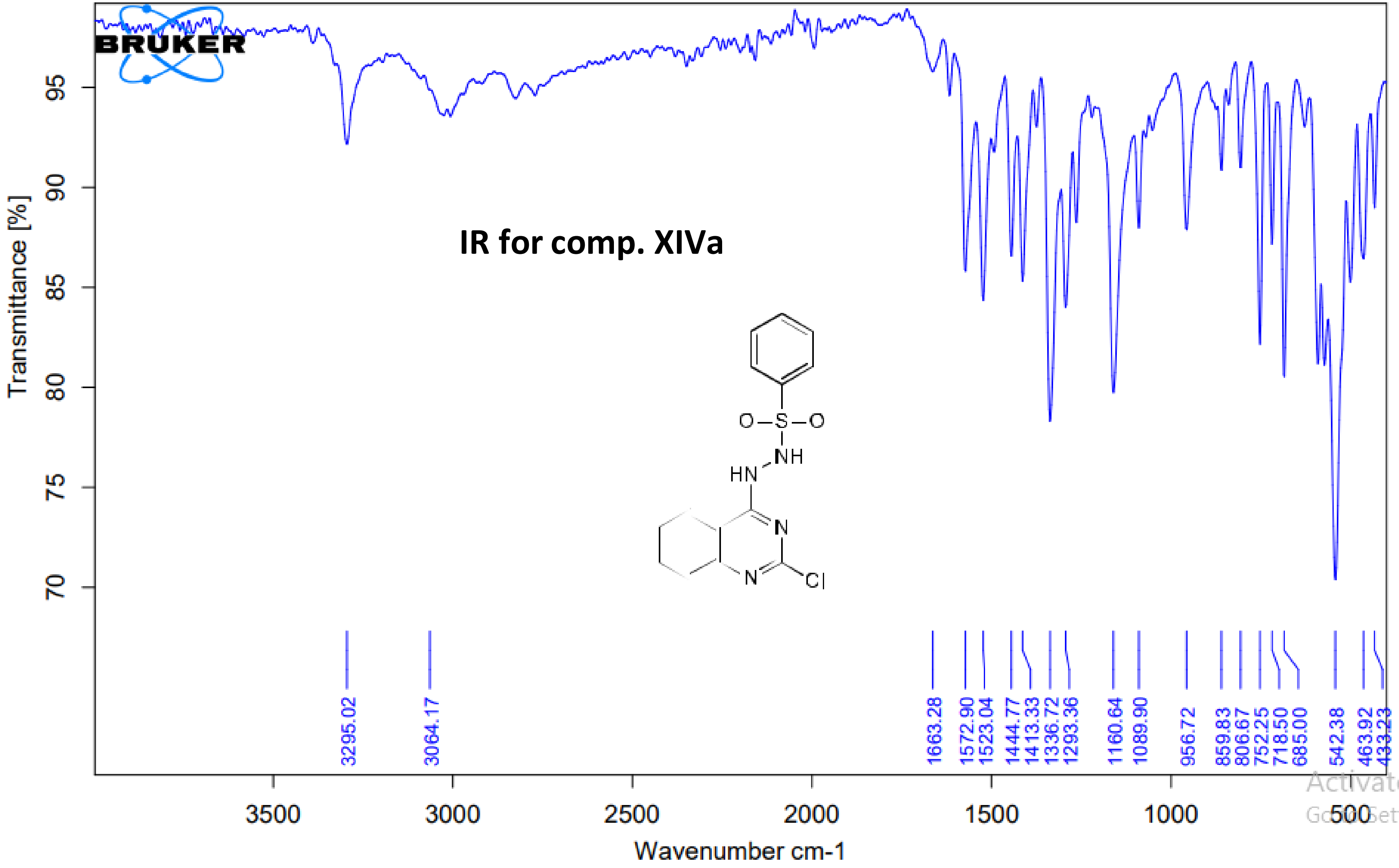

Activate Windo
Go to Settings to ac

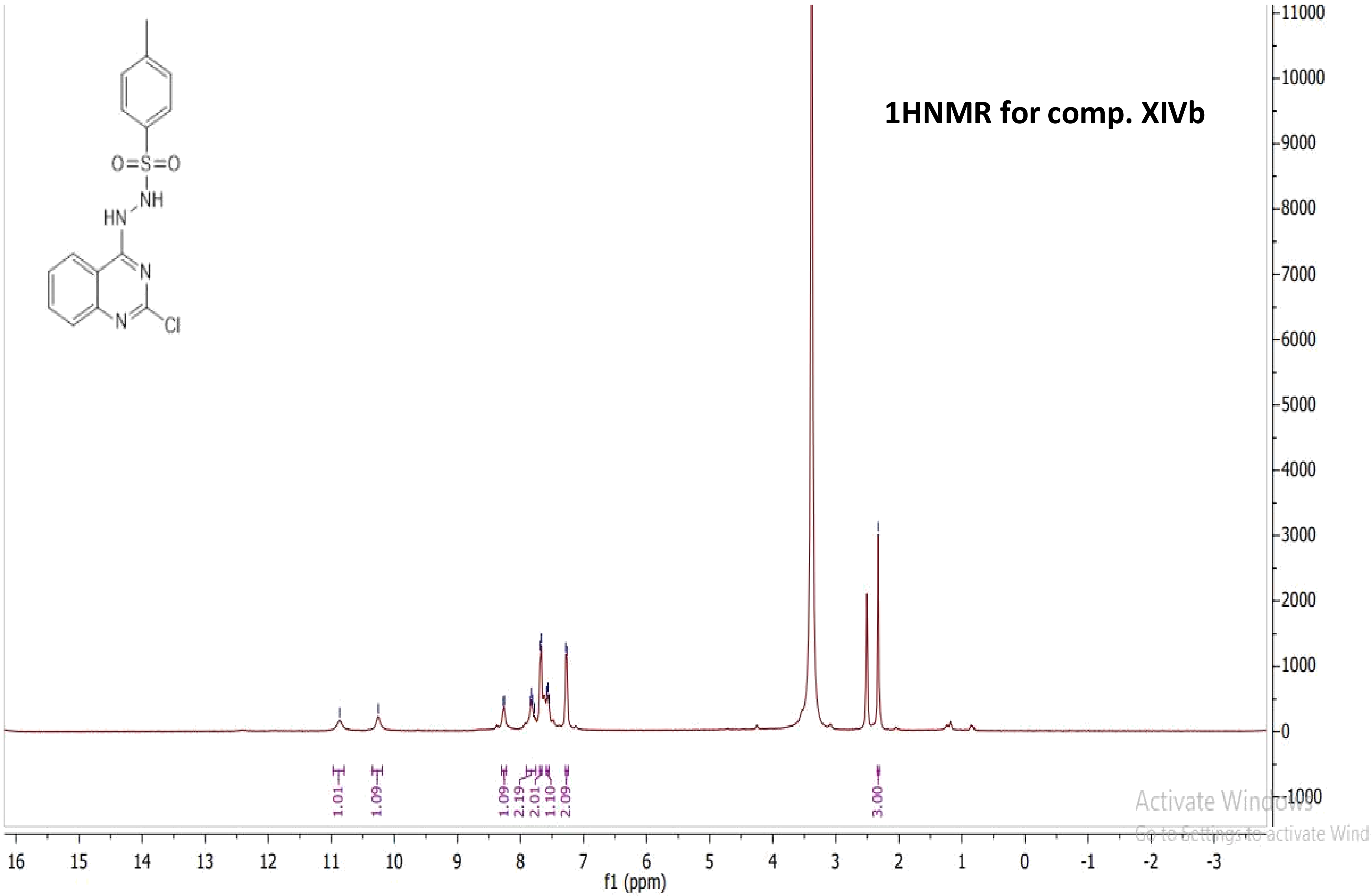
1HNMR for comp. XIVb
O=S=O
NH
HN
N
N
Cl
1.01
1.09
1.09
2.19
2.01
1.10
2.09
3.00
f1 (ppm)
16 15 14 13 12 11 10 9 8 7 6 5 4 3 2 1 0 -1 -2 -3
11000 10000 9000 8000 7000 6000 5000 4000 3000 2000 1000 0 -1000

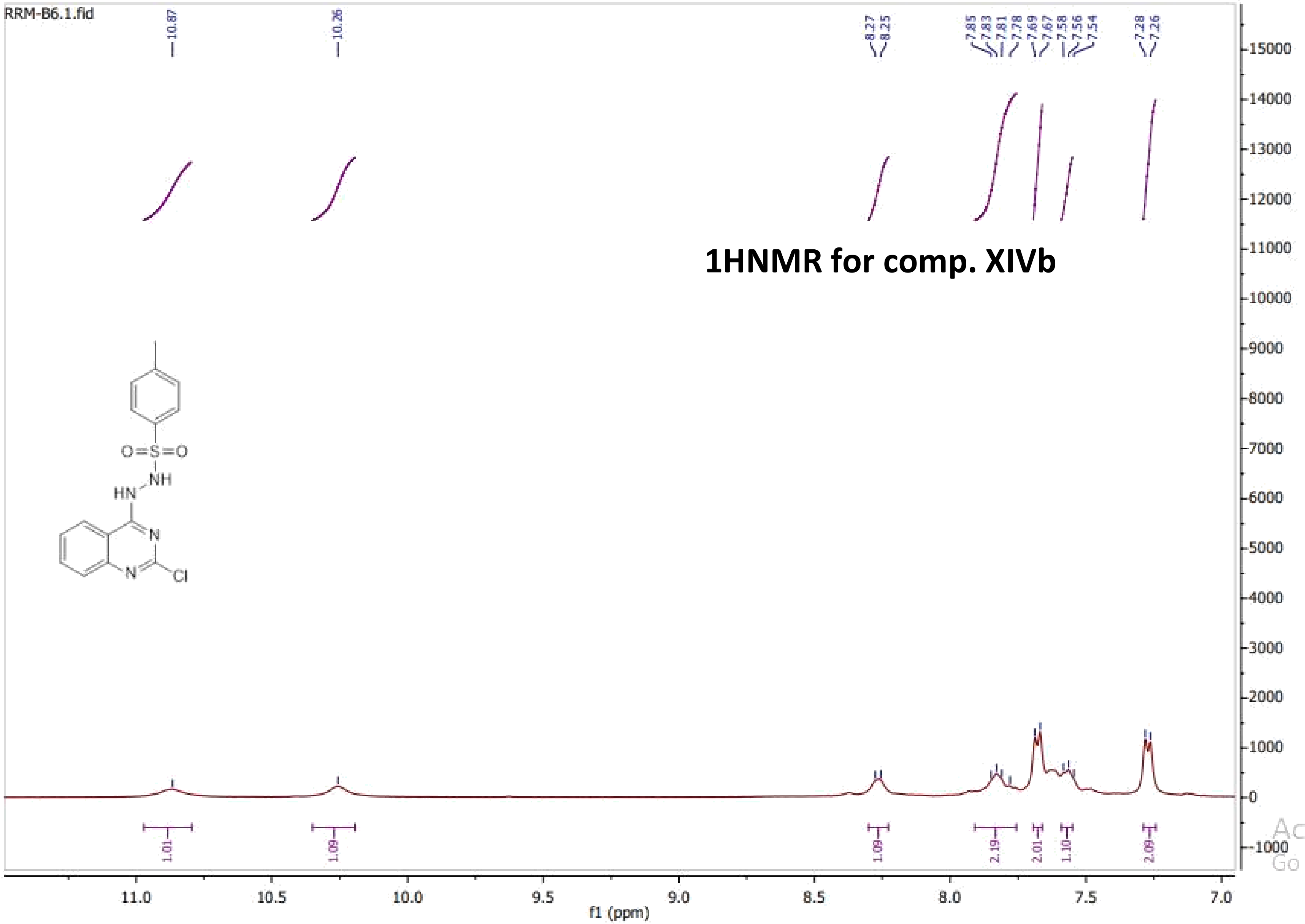
RRM-B6.1.fid
1HNMR for comp. XIVb
10.87
10.26
8.27
8.25
7.85
7.83
7.81
7.78
7.69
7.67
7.58
7.56
7.54
7.28
7.26
1.01
1.09
1.09
2.19
2.01
1.10
2.09
f1 (ppm)
O=S=O
NH
HN
N
N
Cl

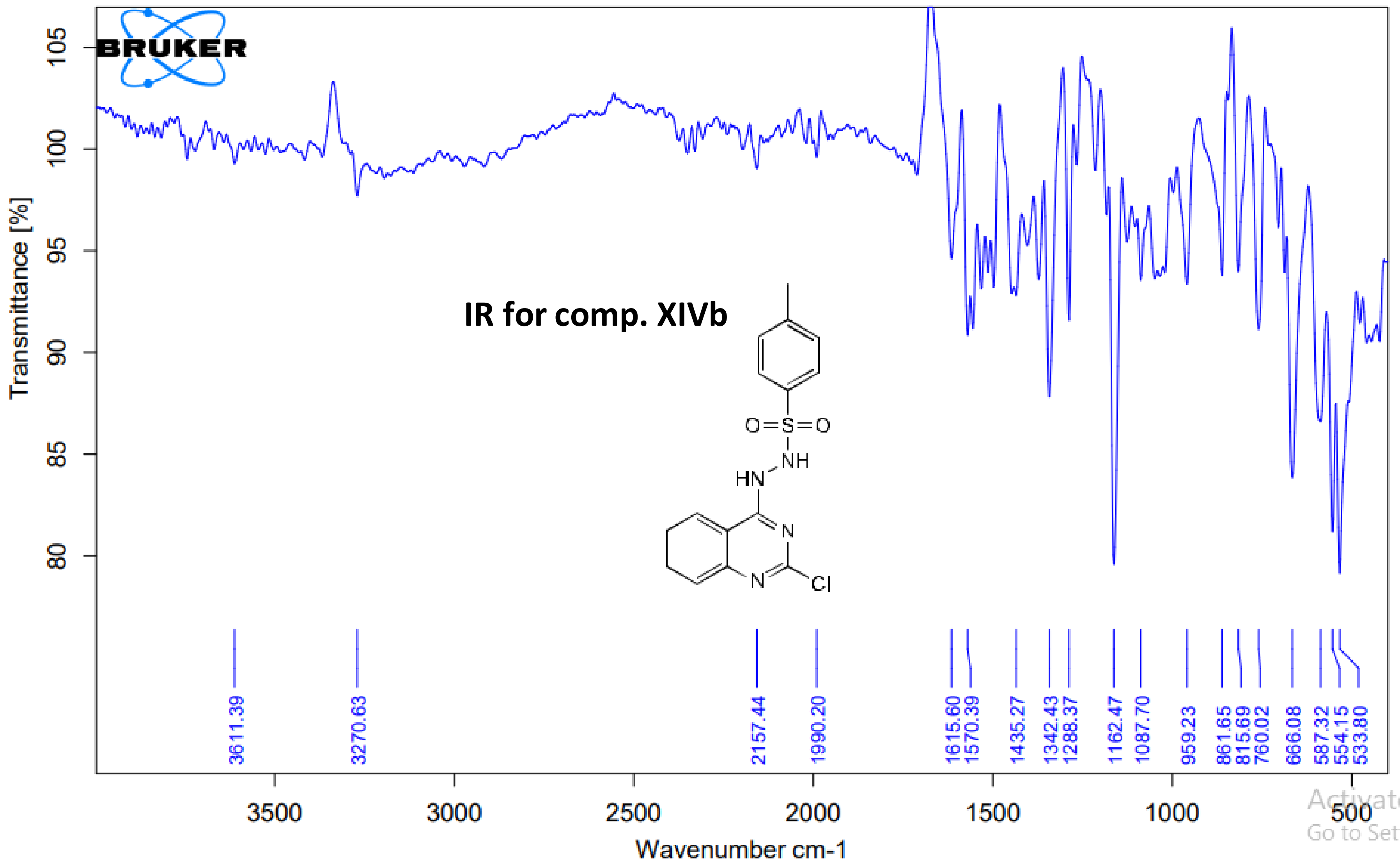

Activate Windo
Go to Settings to ac

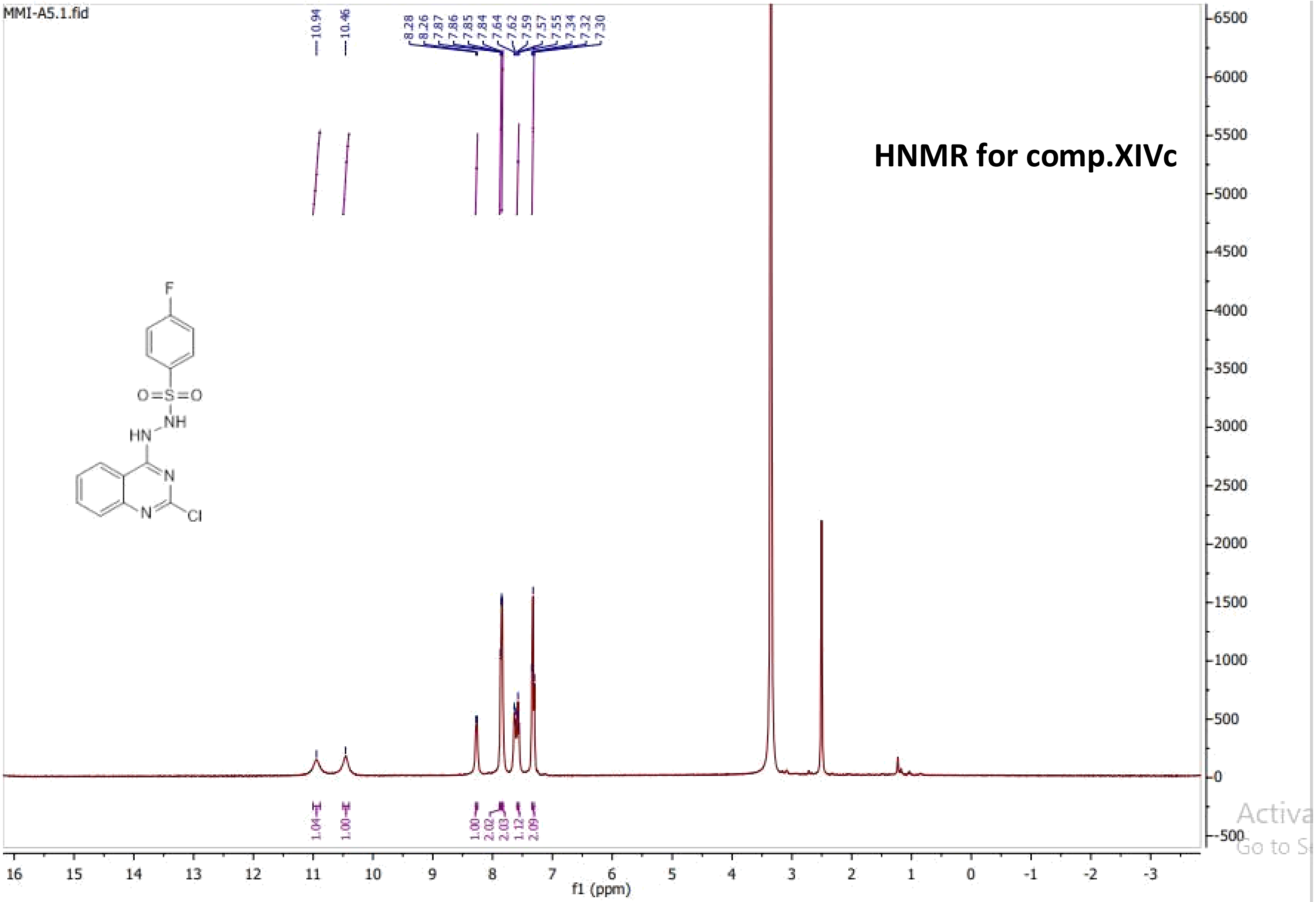

HNMR for comp.XIVc

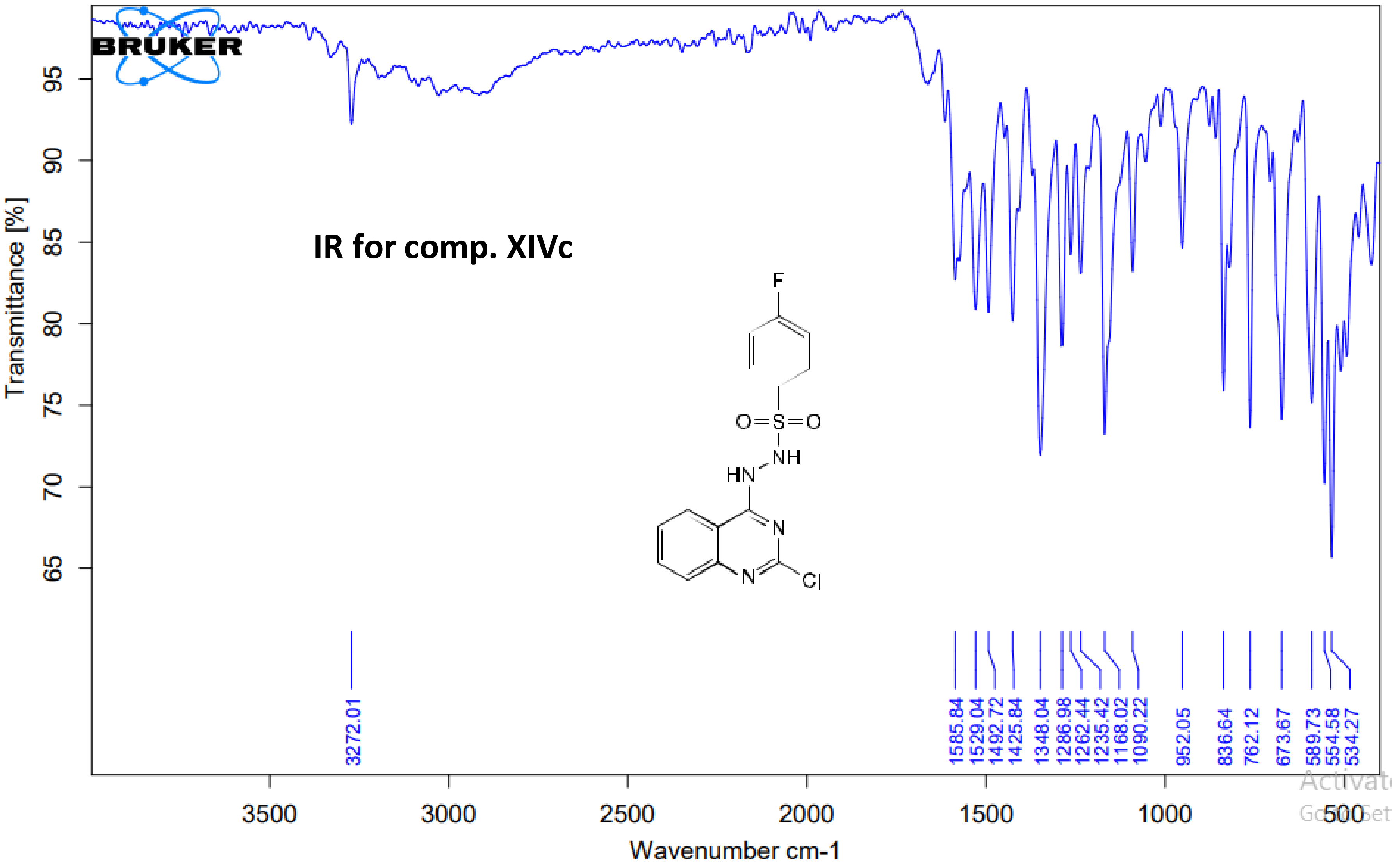
BRUKER
IR for comp. XIVc
Transmittance [%]
95
90
85
80
75
70
65
F
O=S=O
NH
HN
N
N
Cl
3272.01
1585.84
1529.04
1492.72
1425.84
1348.04
1286.98
1262.44
1235.42
1168.02
1090.22
952.05
836.64
762.12
673.67
589.73
554.58
534.27
3500
3000
2500
2000
1500
1000
500
Wavenumber cm-1

1HNMR for comp. XVI

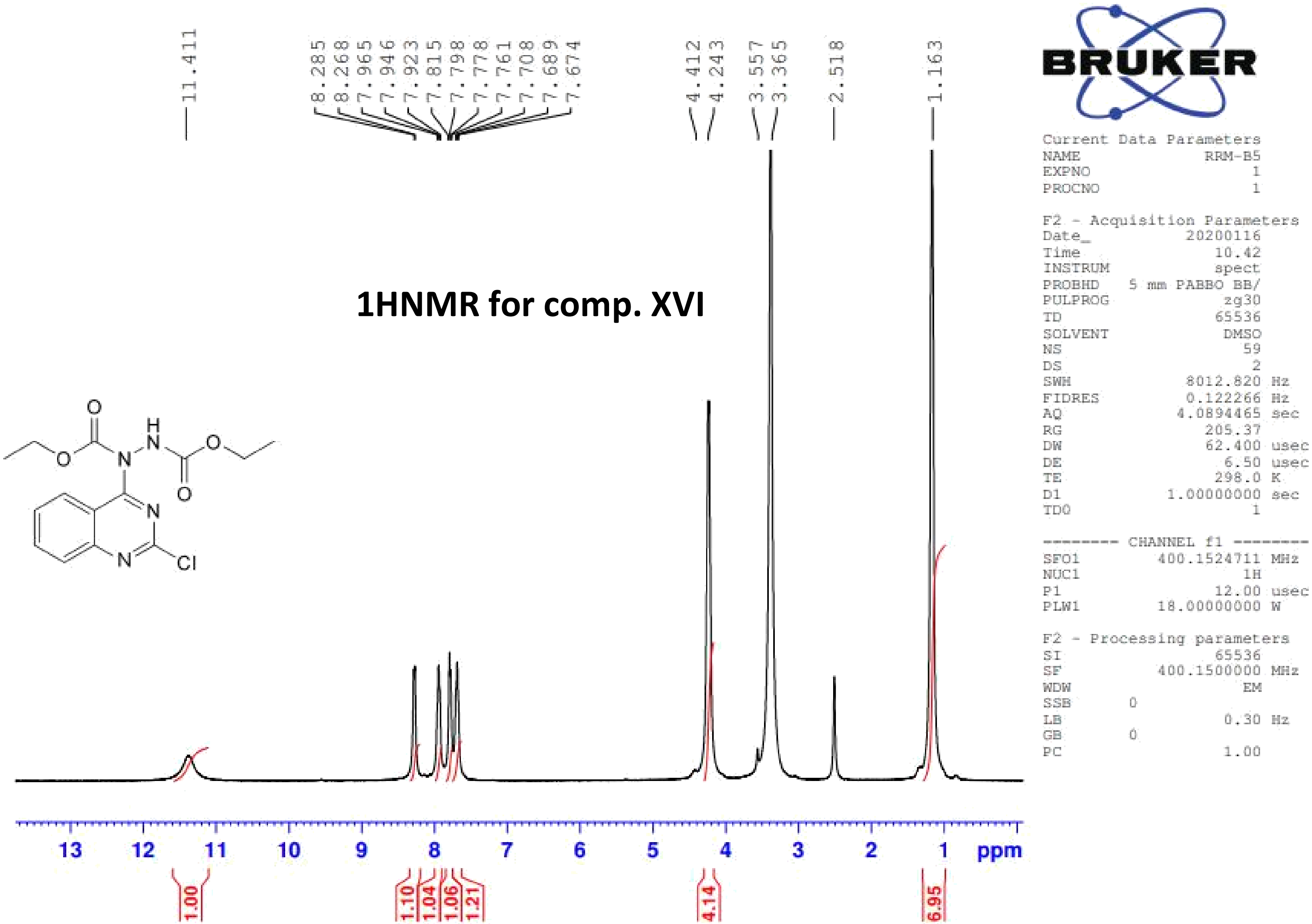

Activate Wir
Go to Settings t

1HNMR for comp.XVI

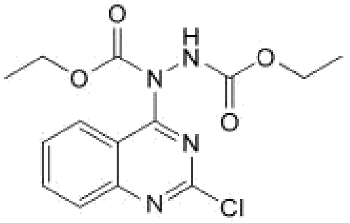

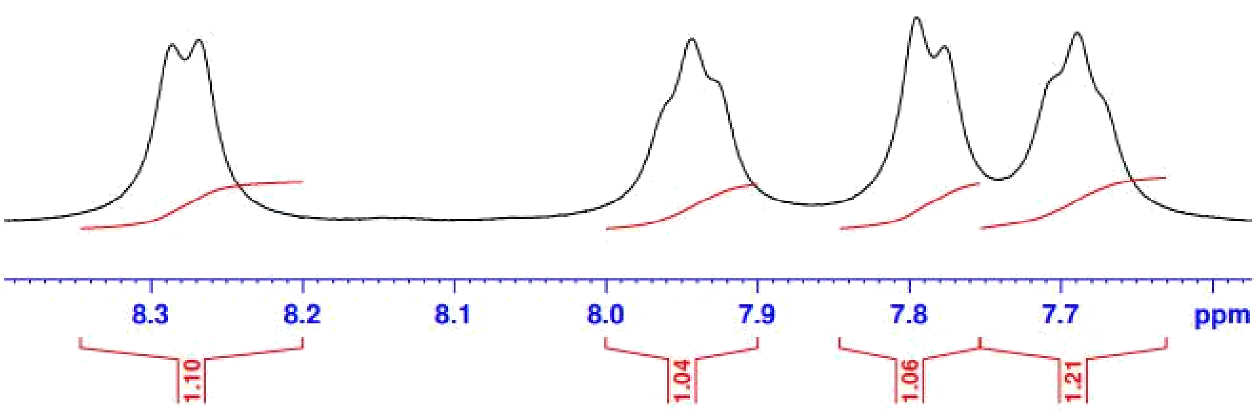

BRUKER

```
Current Data Parameters
NAME                RRM-B5
EXPNO                    1
PROCNO                   1

F2 - Acquisition Parameters
Date_             20200116
Time                 10.42
INSTRUM              spect
PROBHD    5 mm PABBO BB/
PULPROG               zg30
TD                   65536
SOLVENT               DMSO
NS                      59
DS                       2
SWH              8012.820 Hz
FIDRES           0.122266 Hz
AQ              4.0894465 sec
RG                  205.37
DW                  62.400 usec
DE                    6.50 usec
TE                   298.0 K
D1             1.00000000 sec
TD0                      1

======== CHANNEL f1 ========
SFO1          400.1524711 MHz
NUC1                    1H
P1                   12.00 usec
PLW1           18.00000000 W

F2 - Processing parameters
SI                   65536
SF            400.1500000 MHz
WDW                     EM
SSB       0
LB                    0.30 Hz
GB        0
PC                    1.00
```

Activate Wi
Go to Settings t

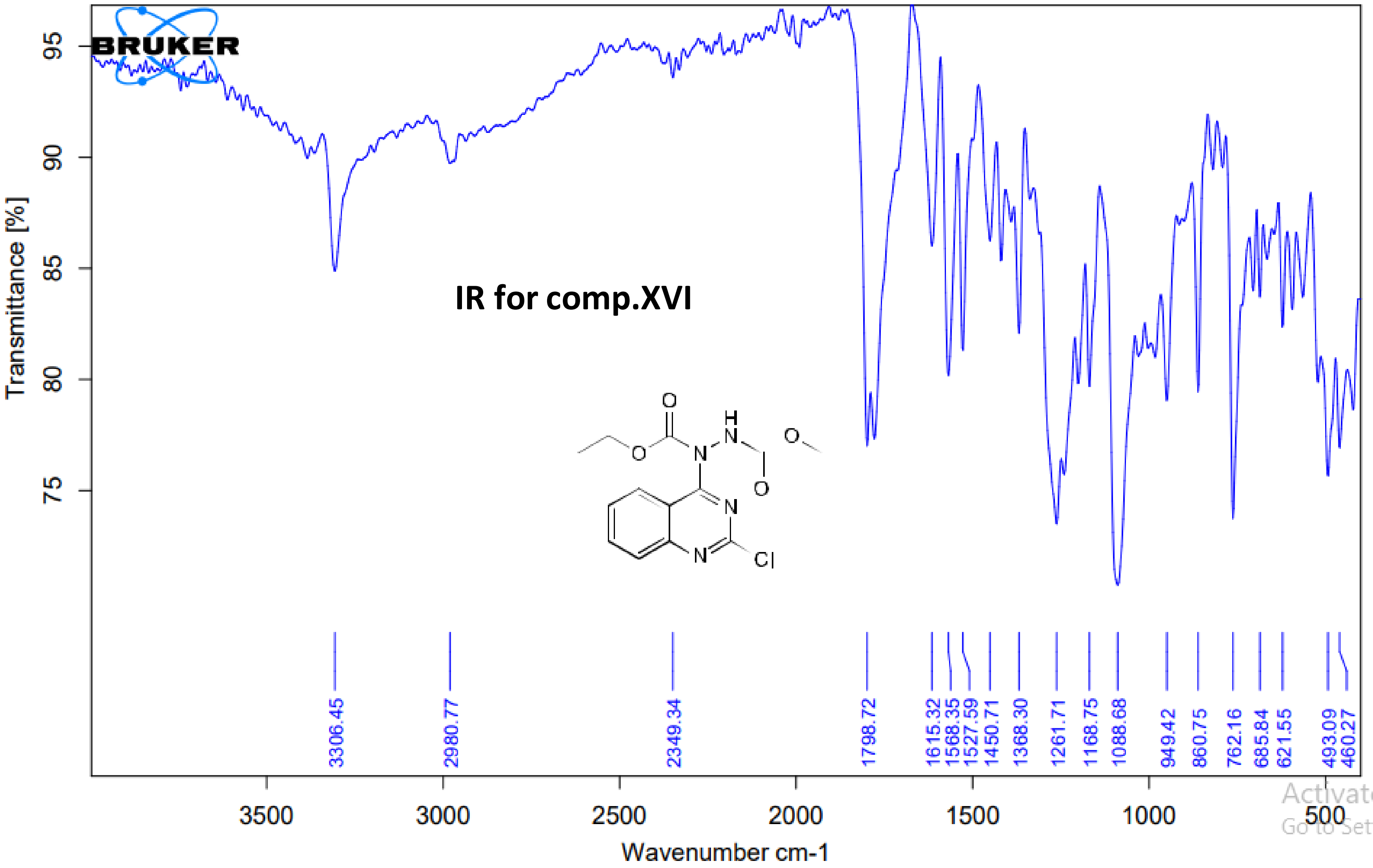

Activate Windo
Go to Settings to ac

1HNMR for comp. XIX

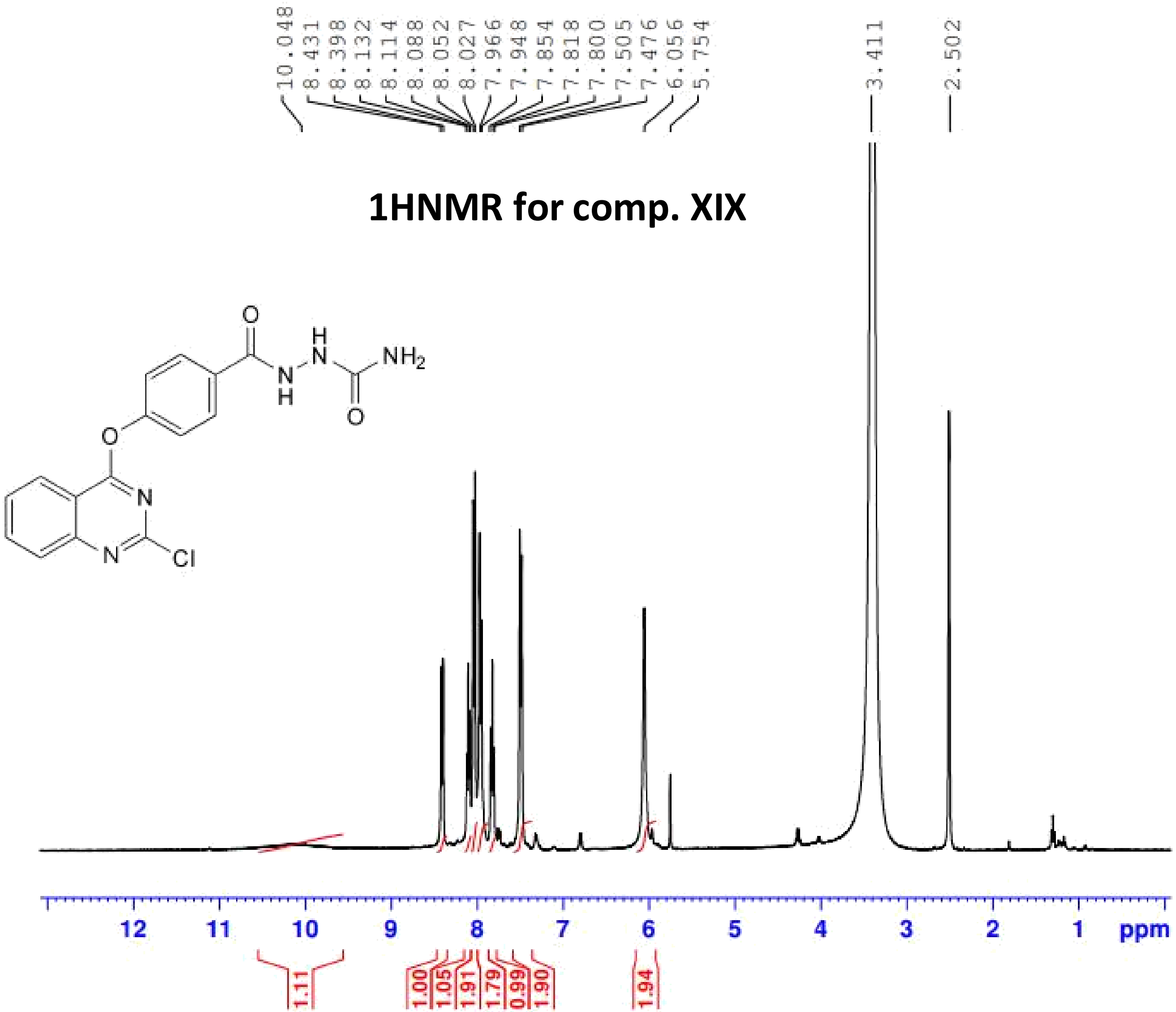

BRUKER

Current Data Parameters
NAME RRM-B1
EXPNO 1
PROCNO 1

F2 - Acquisition Parameters
Date_ 20200116
Time 12.08
INSTRUM spect
PROBHD 5 mm PABBO BB/
PULPROG zg30
TD 65536
SOLVENT DMSO
NS 105
DS 2
SWH 8012.820 Hz
FIDRES 0.122266 Hz
AQ 4.0894465 sec
RG 205.37
DW 62.400 usec
DE 6.50 usec
TE 298.0 K
D1 1.00000000 sec
TD0 1

======== CHANNEL f1 ========
SFO1 400.1524711 MHz
NUC1 1H
P1 12.00 usec
PLW1 18.00000000 W

F2 - Processing parameters
SI 65536
SF 400.1500000 MHz
WDW EM
SSB 0
LB 0.30 Hz
GB 0
PC 1.00

Activate Wi
Go to Settings t

1HNMR for comp. XIX

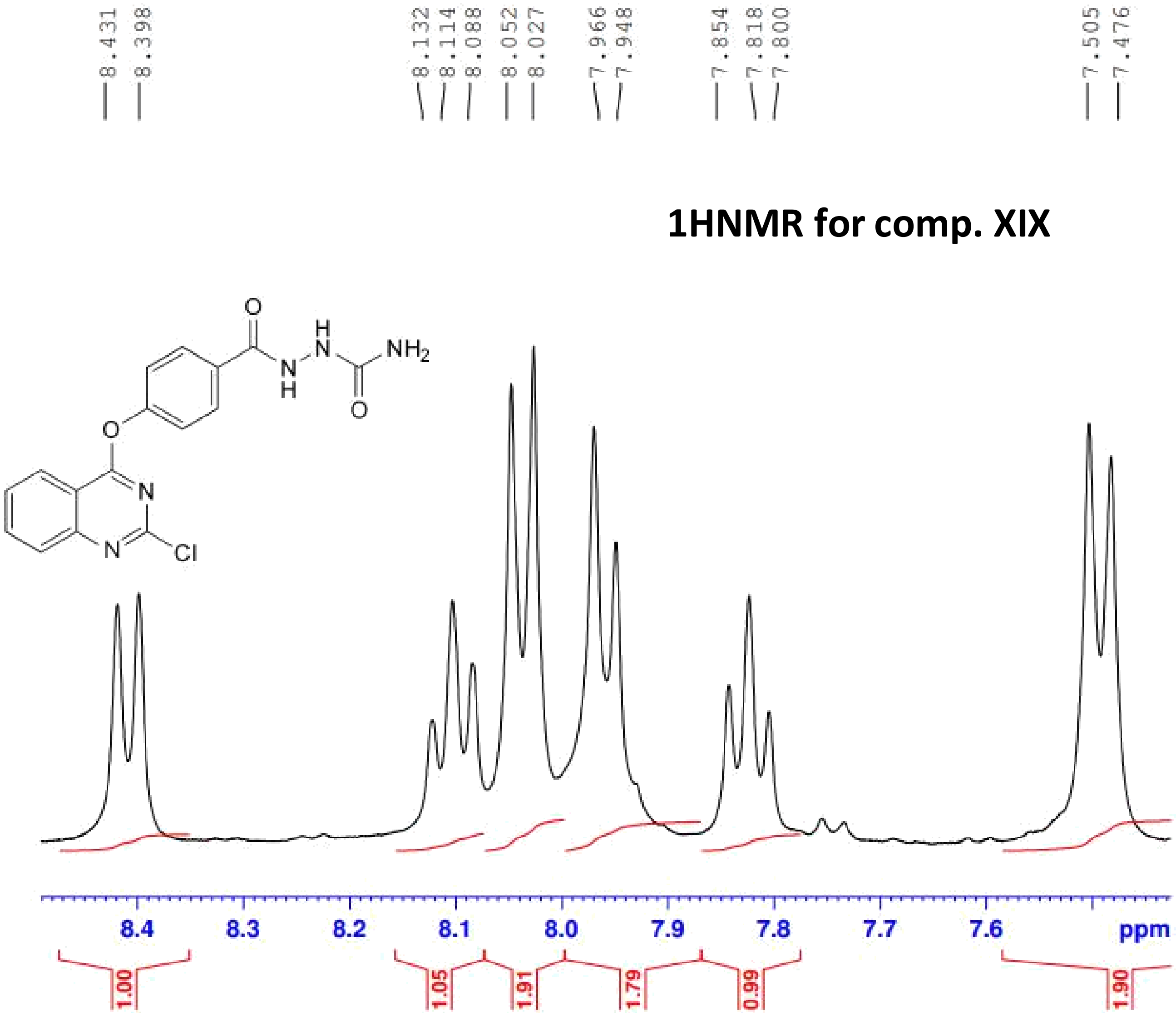

Current Data Parameters
NAME RRM-B1
EXPNO 1
PROCNO 1

F2 - Acquisition Parameters
Date_ 20200116
Time 12.08
INSTRUM spect
PROBHD 5 mm PABBO BB/
PULPROG zg30
TD 65536
SOLVENT DMSO
NS 105
DS 2
SWH 8012.820 Hz
FIDRES 0.122266 Hz
AQ 4.0894465 sec
RG 205.37
DW 62.400 usec
DE 6.50 usec
TE 298.0 K
D1 1.00000000 sec
TD0 1

-------- CHANNEL f1 --------
SFO1 400.1524711 MHz
NUC1 1H
P1 12.00 usec
PLW1 18.00000000 W

F2 - Processing parameters
SI 65536
SF 400.1500000 MHz
WDW EM
SSB 0
LB 0.30 Hz
GB 0
PC 1.00

Activate Wi
Go to Settings t

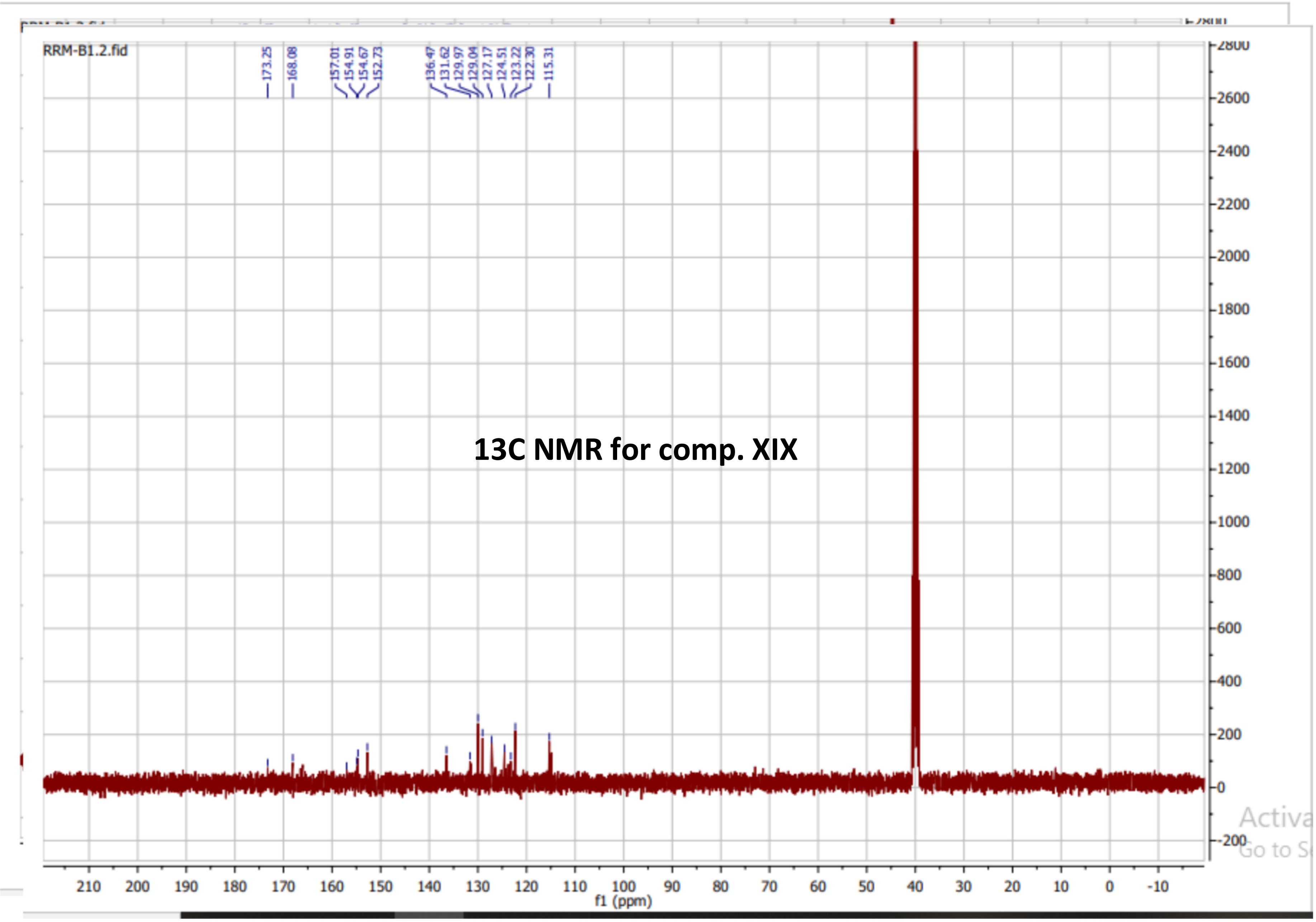
RRM-B1.2.fid
173.25
168.08
157.01
154.91
154.67
152.73
136.47
131.62
129.97
129.04
127.17
124.51
123.22
122.30
115.31
13C NMR for comp. XIX
f1 (ppm)

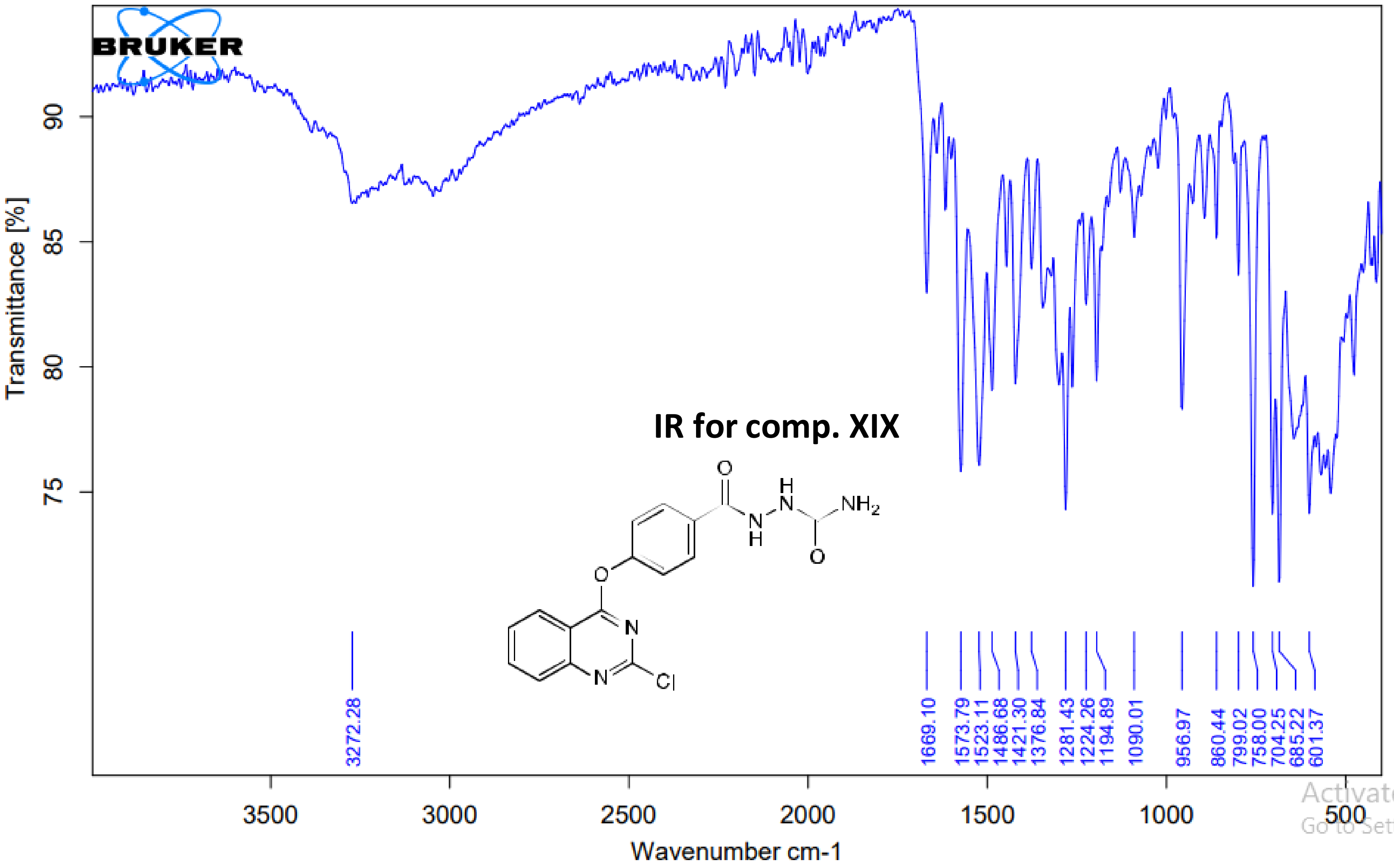

Activate Windo
Go to Settings to ac

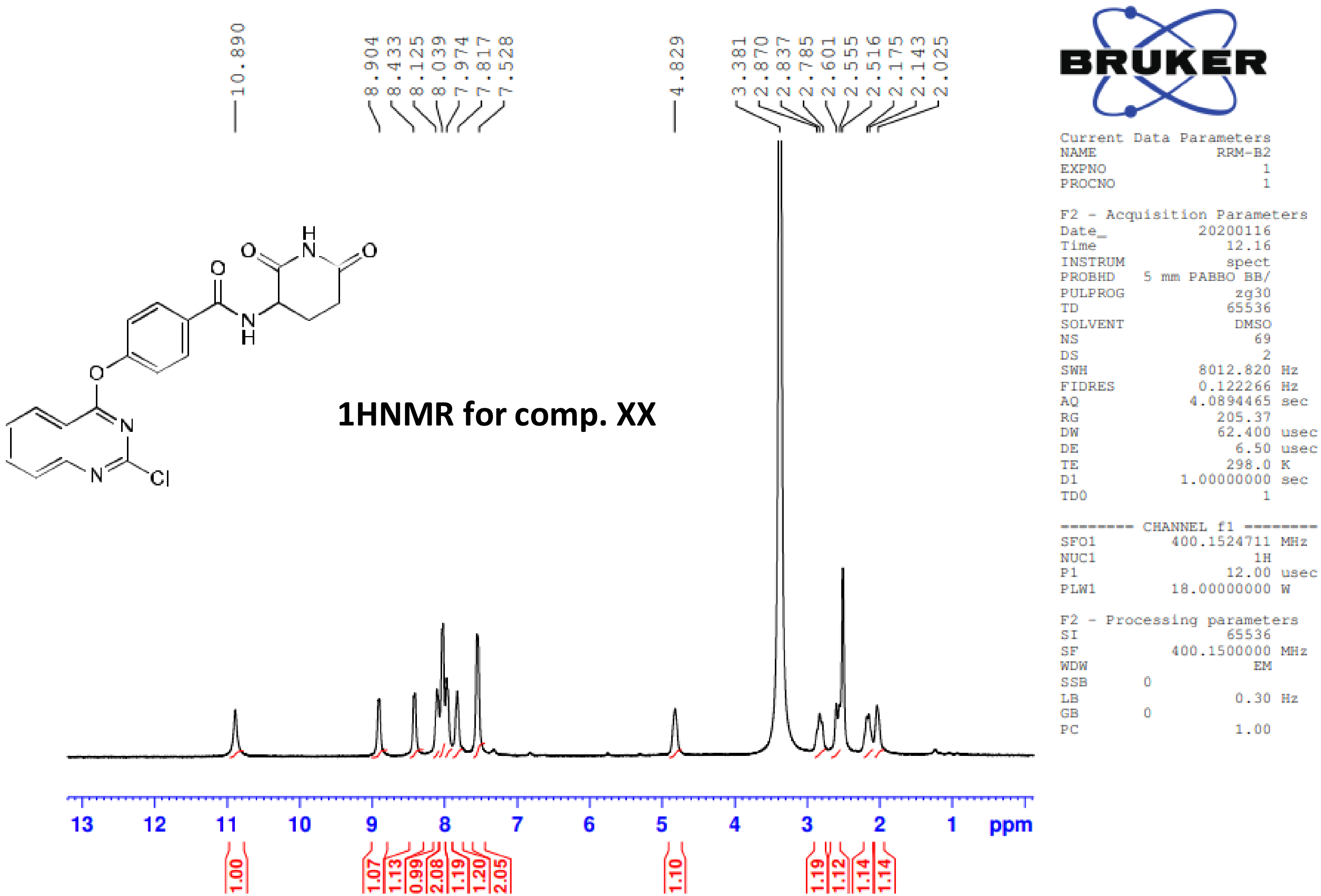

Activate Wi
Go to Settings t

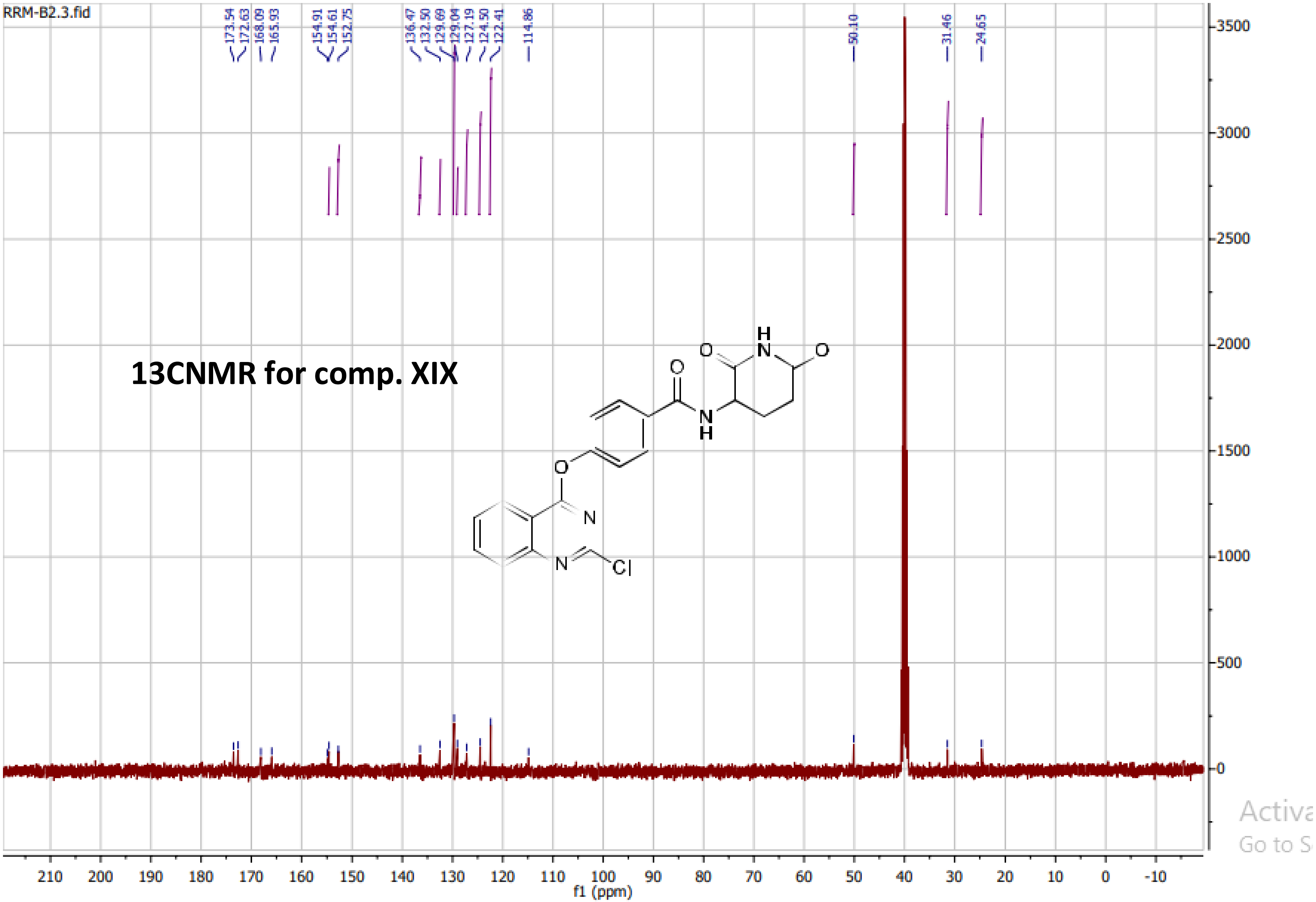

Activa
Go to S

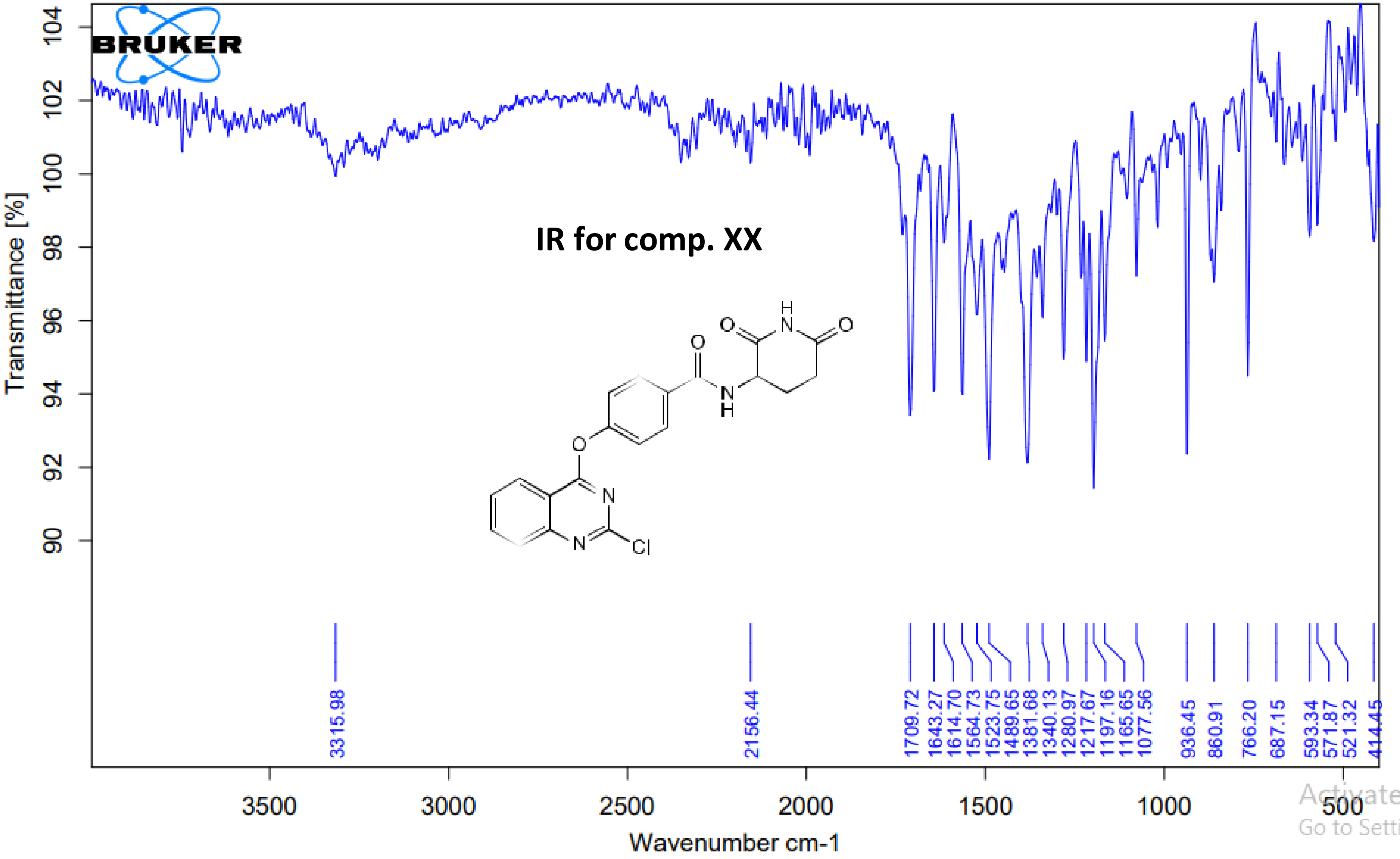
BRUKER
IR for comp. XX
Transmittance [%]
104
102
100
98
96
94
92
90
3315.98
2156.44
1709.72
1643.27
1614.70
1564.73
1523.75
1489.65
1381.68
1340.13
1280.97
1217.67
1197.16
1165.65
1077.56
936.45
860.91
766.20
687.15
593.34
571.87
521.32
414.45
3500
3000
2500
2000
1500
1000
500
Wavenumber cm-1
O
N
H
Cl
